# Wind speed and power forecasting using Bayesian optimized machine learning models in Gabal Al-Zayt, Egypt

**DOI:** 10.1038/s41598-025-13140-x

**Published:** 2025-08-05

**Authors:** Nehal Elshaboury, Haytham Elmousalami

**Affiliations:** 1 Construction and Project Management Research Institute, Housing and Building National Research Center, Giza, Egypt; 2 Department of Infrastructure Engineering, Faculty of Engineering and Information Technology, The University of Melbourne, Melbourne, VIC 3010 Australia; 3 Projects Section Head and Project Management Professional (PMP) at General Petroleum Company, Nasr City, Egypt

**Keywords:** Ensemble machine learning, Bayesian optimization, Wind speed prediction, Wind power prediction, Developing countries, Environmental sciences, Energy science and technology

## Abstract

Accurate wind speed and power forecasts are essential for applications involving renewable wind energy. Ten machine learning techniques, including single and ensemble models, are compared, and evaluated in this study over a range of time scales. The outcomes of the wind speed prediction (WSP) model are used as inputs for the wind power prediction (WPP) model in a wind speed and power integration prediction system. The accuracy of various machine learning models is compared using several evaluation metrics, such as Pearson’s correlation coefficient (R), explained variance (EV), mean absolute percentage error (MAPE), mean square error (MSE), and concordance correlation coefficient (CCC). For WSP, the light gradient boosting machine (LGBM), extreme gradient boosting, and bagged decision tree (BDT) algorithms accurately predict wind speed across different time scales, with MAPE, MSE, EV, R, and CCC values ranging from 2.641 to 12.274%, 0.044 to 0.953, 0.888 to 0.994, 0.943 to 0.997, and 0.939 to 0.997, respectively. For WPP, the LGBM and BDT algorithms demonstrate strong predictive performance across different time scales, with MAPE, MSE, EV, R, and CCC values ranging from 0.277 to 186.710%, 0.927 to 9444.576, 0.970 to 1.000, 0.985 to 1.000, and 0.985 to 1.000, respectively.

**Supplementary Information:**

The online version contains supplementary material available at 10.1038/s41598-025-13140-x.

## Introduction

To address the challenges associated with wind energy variability, advanced forecasting techniques and data-driven strategies are crucial for maximizing its efficiency and reliability. The unpredictable nature of wind stems from complex meteorological and geographical factors, making it difficult to ensure a stable and consistent power supply. This variability poses integration challenges for energy grids, necessitating robust predictive models that can enhance wind energy forecasting accuracy and facilitate better grid management^1,2^.

The vast amounts of data collected from wind power plants, including meteorological parameters such as temperature, humidity, air pressure, wind direction, and wind speed, along with turbine performance metrics, provide a foundation for optimizing operations. Machine learning (ML) and artificial intelligence techniques have emerged as powerful tools to analyze and interpret these datasets, enabling more precise wind speed and power output predictions. These technologies can improve turbine efficiency, enhance maintenance strategies through predictive analytics, and support real-time decision-making for energy dispatch^3,4^.

Moreover, integrating artificial intelligence-driven forecasting models with energy storage systems and smart grid technologies can mitigate wind power fluctuations, improving grid stability and reliability. By leveraging these advancements, wind energy can play a pivotal role in reducing carbon emissions, supporting the transition to sustainable energy systems, and advancing global efforts toward a low-carbon future^4,5^.

### Research problems

Despite advancements in forecasting methods, challenges specific to wind energy forecasting persist, distinguishing it from other time-series prediction tasks^5,6^. The high variability and stochastic nature of wind, influenced by complex meteorological phenomena, make accurate predictions difficult. Additionally, the spatial and temporal dependencies in wind patterns require sophisticated modeling approaches to capture these dynamics effectively^7,8^. The need for real-time data processing and integrating various data sources further complicates the forecasting process. These challenges necessitate specialized models capable of addressing the unique characteristics of wind data^9,10^.

### Research objectives

This paper aims to address these challenges by conducting a comprehensive comparison of single and ensemble ML models for wind speed prediction (WSP) and wind power prediction (WPP) across various time horizons. Previous studies have often overlooked tree-based models in this context. The novelty of this research lies in developing an integrated system based on Bayesian-optimized ML algorithms for WSP and WPP, ensuring optimal precision and computational efficiency. This optimization algorithm determines the best hyperparameter configurations for ML models. Additionally, the paper highlights the strengths and weaknesses of various ML algorithms, enabling the selection of optimal models with efficient computational time and memory usage^11,12^.

By addressing the specific challenges inherent in wind energy forecasting, this research contributes to the advancement of more accurate and reliable forecasting models, thereby supporting the integration and operational efficiency of wind energy in power systems. Therefore, the paper’s key contribution lies in the development of an integrated system utilizing Bayesian-optimized machine learning algorithms to enhance the accuracy of wind speed and power forecasting across various time horizons. This approach improved prediction models that can be instrumental in optimizing wind farm operations and ensuring grid stability.

## Literature review

The significance of quantitative forecasting techniques for precisely estimating wind power and speed has grown during the last 20 years. The use of ML and deep learning (DL) for wind energy forecasting has been examined in several review studies^11,12^. The unpredictable aspect of wind energy has been addressed by a variety of statistical learning techniques^13^. For WSP and WPP, ML models and artificial intelligence approaches including multiple linear regression (MLR), multilayer perceptron (MLP) networks, and support vector machines (SVM) have been extensively employed^14^.

For short-term WPP, backpropagation training techniques have been used with artificial neural networks (ANNs)^15^. For probabilistic wind power prediction, ANN frameworks have incorporated the radial basis function (RBF) network^16^. For 30-min-ahead WPP, a hybridization of SVM and wavelet methods has been suggested, with wind speed and temperature being recognized as important predictors^17^. It has been demonstrated that combining supervised and unsupervised methods improves the root mean squared error by 37%^18^. In India and Turkey, the primary predictors utilized in ANN-based models for wind energy prediction are average wind speed, relative humidity, and generating hours.^19,20^.

A hybrid clustering technique in conjunction with a wavelet-based neural network has been used to create a one-hour short-term WPP model. Furthermore, a five-minute extremely short-term probabilistic WPP model was created using recorded wind directions and speeds from a Dual-Doppler radar system, and it performed better than deterministic radar-based forecasts^21,22^. In order to improve overall performance, a gravitational search method was used with the least squares SVM to create a 10-min very short-term WPP model^23^. A better model for RBF neural networks model was also introduced, utilizing parameter initialization techniques to enhance accuracy and convergence^24^.

DL is essential for increasing the precision of large data analysis for wind farms. Singular spectrum analysis has been used to reduce noise using a hybrid deep learning model called a convolutional SVM, which combines convolutional neural networks (CNN) and SVM. Furthermore, trend information has been extracted from the original wind speed data using empirical mode decomposition^25,26^. Recurrent neural networks (RNN) and deep learning were used to create a short-term spatiotemporal wind speed forecasting technique^27^. Additionally, a DL-based WSP model that combines an enhanced long short-term memory (LSTM) network with wavelet transform has shown comparatively good prediction accuracy^28^. For WSP, a CNN-LSTM hybrid architecture has also been presented, which successfully captures spatial and temporal correlations. This architecture has performed better than current techniques for short-term forecasts.

Three new hybrid models based on RNN gated recurrent unit neural networks, and SVM were developed because of a comparative study on short-term wind speed forecasting using ANN. Additionally, a new prediction framework was put forward. Furthermore, the literature has evaluated decomposition-based hybrid wind energy forecasting models in detail, emphasizing their use in wind energy forecasting^29^. These equations are based on radiative and chemical processes, hydrodynamic models, and thermodynamic theory. The primary meteorological characteristics that NWP forecasts are temperature, humidity, wind direction, and speed. For wind power prediction, NWP can be modified and combined with machine learning methods^30^. Nonetheless, several scientific and technological obstacles still stand in the way of NWP’s future advancement, including:

1. Two of the main scientific issues facing contemporary NWP are uncertainty formulation and physical process parameterization.
2. One of the biggest obstacles to real-time forecasting applications is still the processing power needed for high-resolution NWP models.
3. High-resolution meteorological observations and sophisticated data assimilation techniques are required since the quality and frequency of input data have a significant impact on the accuracy of NWP-based wind forecasts.

Table 1^31,32^ illustrates the four categories into which wind forecasting techniques can be divided based on the time scale. Different temporal scopes can classify WSP and WPP^33^. Preload sharing, load tracking, and turbine control all depend on short-term forecasts^34^. Daily forecasts are useful for players in the power market, while weekly-ahead forecasts are necessary for wind farm maintenance activities. In short-term markets, intra-day forecasts are employed to keep supply and demand in balance in real time^35,36^. High-precision wind power forecasting is also a crucial operational challenge because numerous wind farms are connected to electrical networks.

**Table 1:** Wind speed and power prediction time scale.

Time horizon	Range	Applications
Very short-term	Few seconds to 30 min ahead	Electricity market management and trading Regulation actions planning and balancing Virtual power plants
Short-term	30 min to 6 h ahead	Load decreasing and increasing actions Load dispatch management Intraday trading
Medium-term	6 h to one day ahead	Operational security precautions Generators operating management Day-ahead trading
Long-term	One day to one week or more ahead	Maintenance planning and scheduling Reserve management

High-precision wind speed and power forecasting are essential for effective energy management. Despite the significant advancements in ML and DL techniques for WSP and WPP, several research gaps remain unaddressed:

- Previous studies have not provided a thorough comparison of different optimized ML algorithms at varying forecasting time scales, particularly in terms of prediction accuracy and computational efficiency.
- While Bayesian optimization has been successfully applied for optimizing hyperparameters in different domains, its application in wind power prediction remains underexplored. Bayesian optimization can enhance the predictive performance of wind forecasting models by systematically tuning hyperparameters to maximize accuracy and minimize computation time.
- The majority of existing studies focus on regions with established wind energy infrastructure, such as Europe and North America, while limited research has been conducted on high-potential wind locations such as Gabal Al-Zayt in Egypt. Given the unique meteorological characteristics of this region, more research is needed to improve parameterization techniques and validate ML-based forecasting methods.
- Developing NWP models with ML integration requires significant computational power due to the complex nonlinear differential equations and the need to process large datasets. The scalability of ML models to handle diverse and large-scale meteorological data remains a challenge.

By addressing these research gaps, this study can enhance the reliability and applicability of ML-based wind power forecasting methods, contributing to the development of more efficient and resilient renewable energy systems.

## Machine learning models

### Single machine learning

Models that use one statistical learning method for a particular task are known as single machine learning models^37,38^. Multiple linear regression is a machine learning technique that uses Eq. 1 to use the gathered data for regression and classification applications.

1 $$Y = B_{0} + B_{1} X_{1} + B_{2} X_{2} + \ldots B_{n} X_{n}$$

where Xi is the independent variable, Bi is the variable coefficient, Y is the dependent variable, and B0 is a constant. The provided data is linearly fitted by the regression technique with a low prediction error^39,40^. SVM is a supervised learning technique that may be applied to issues involving regression and classification^41,42^. It maximizes the hyperplane distance and margin. The hyperplane distance based on the two class borders can be optimized using Eq. 2^43,44^.

2 $$Linear\;SVM = \left\{ {\frac{{\begin{array}{*{20}c} {W.X_{i} + b \ge 1,} & {if} & {y_{i} \ge 0} \\ \end{array} }}{{\begin{array}{*{20}c} {W.X_{i} + b < - 1, } & {if} & {y_{i} < 0} \\ \end{array} }}} \right.$$

### Ensemble machine learning

Several algorithms are combined in ensemble machine-learning techniques to improve overall performance and accuracy. The outcomes of many base learners are combined in the ensemble model^45,46^. Processing noisy data, complex data structures, and high dimensional data are the main benefits of ensemble machine learning. Ensemble machine learning, however, raises the total computational complexity^47,48^. Here are synopses of a few well-known ensemble models:

- ET, also known as an extra tree, builds a forest of decision trees using random subsets of features and training data. As such, it can achieve high accuracy, reduce overfitting, and handle noisy data. However, the main drawback of ET is that it creates many random trees, which increases its computational cost when compared to single decision trees. When compared to individual decision trees, ET models are typically harder to understand.
- Random forest (RF) combines the predictions of multiple decision trees through majority voting or averaging to manage noisy data, improve accuracy, and lessen overfitting. It can be computationally intensive, especially when dealing with a large number of trees. It might not perform as well as some other methods on high-dimensional data^49^. Its medium complexity strikes a good balance between precision and intricacy.
- Decision tree (DT) is a tree-based model that recursively splits the data into subsets based on the most important feature at each node of the tree. It is a transparent model that can work with both categorical and numerical data. This algorithm is robust to outliers, but it is sensitive to small variations in the training data. Its accuracy can vary; it is sensitive to the quality and quantity of data. Moreover, it may be vulnerable to overfitting when it is overly complex. When decision trees are shallow, they are simple, but as they go deeper, they can get more complicated.
- Bootstrapping aggregating or bagging is a technique used by bagged decision trees (BDT) to reduce the variation and overfitting of individual decision trees^50^. Despite this, it might not offer the same degree of bias reduction as boosting techniques. Compared to RF or ET, BDT models may be easier to understand because each tree is constructed separately. BDT can be computationally intensive, particularly if a lot of decision trees need to be created. It has medium complexity, falling between individual decision trees and sophisticated ensemble methods.
- GB was originally developed as a classification algorithm that combines weak learners and forms strong learners through an iterative optimization algorithm^51^. Later, Friedman extended the boosting algorithm to regression problems. This algorithm aims to minimize the loss function. It initializes the model through the first guess, and then fits a new decision tree into the existing model to update the residual difference until reaching the specified maximum number of iterations.
- Light gradient boosting machine (LGBM) is a relatively new GB decision tree algorithm commonly used in data mining, including regression, classification, and ranking. This algorithm contains two unique techniques, which are exclusive feature bundling and gradient-based one-sided. Therefore, compared to the traditional GB decision tree-based model, LGBM can realize the vertical growth of the tree, which proves its ability to efficiently process large-scale data.
- Extreme gradient boosting (XGBoost) uses parallel computation to process noisy and high-dimensional data and regularization terms to reduce overfitting^52,53^. In comparison to simpler models, it may require more hyperparameter tuning and is computationally intensive, especially when dealing with multiple trees^54,55^.
- Adaptive boosting algorithm (AdaBoost) excels at developing a powerful model by combining multiple weak learners (often decision trees). It adaptively combines multiple weak learners to increase accuracy while preserving some interpretability, giving it a medium level of complexity^56^. It works well with various data types and is less likely to overfit. Nevertheless, its performance could be impacted by noisy data and outliers because of its sensitivity. Additionally, training could take longer than with individual weak learners.

### Bayesian hyperparameter optimization

ML algorithms usually use multiple hyperparameters to optimize performance and increase prediction accuracy during model training. Bayesian optimization finds the ideal hyperparameters iteratively using a probabilistic model^57^. An acquisition function that balances exploration and exploitation is used to select the subsequent set of hyperparameters, and the target function for a new set of hyperparameters is predicted using the probabilistic model.

When compared to grid search and random search, Bayesian optimization offers several benefits^58^: (1) it directs the search toward the best hyperparameters using the results of earlier evaluations, (2) it can manage objective functions that are noisy and non-convex, which are typical in ML problems, and (3) it may be used to continuous and discrete hyperparameters.

This paragraph introduces the various stages involved in Bayesian hyperparameter optimization^58–60^: Setting minimum and maximum values for each hyperparameter that need to be optimized is the initial step in defining the search space for those hyperparameters. In Online Appendix A, descriptions of the hyperparameters for the ML models are provided. The hyperparameters are then used to build an objective function, which yields coefficient of determination (*R*^*2*^) to be maximized. The third step involves selecting a common option, such as a Gaussian process model. A few randomly chosen hyperparameter combinations are then tested using the objective function to initialize the surrogate model, and these first assessments are employed to train the surrogate model. The following step is to choose the subsequent hyperparameters to assess in light of the surrogate model. The predicted improvement in the objective function based on the existing surrogate model is measured using an acquisition function. The chosen hyperparameters are then used to assess the objective function. The anticipated improvement (EI) function, which has become a popular option for the acquisition function, is defined as per Eq. 3:

3 $$EI\left( x \right) = \left\{ {\begin{array}{*{20}c} {\left( {(E\left[ {f_{min} } \right] - f\left( x \right)} \right)P\left( {f\left( x \right) \le f_{min} } \right)} & { if\; \sigma \left( x \right) > 0} \\ 0 & {otherwise} \\ \end{array} } \right.$$

Based on the normal distribution’s cumulative distribution function, the probability $P\left( {f\left( x \right) \le f_{min} } \right)$ is calculated. Updating the surrogate model and choosing and assessing new hyperparameters are continued until a stopping criterion is satisfied. At that point, the algorithm concludes, and the best hyperparameters found during this iterative process are reported as the final result.

### Performance evaluation measures

Predictive ML algorithms can be evaluated and tested using mean absolute percentage error (MAPE), mean square error (MSE), explained variance (EV), Pearson’s correlation (R), and concordance correlation coefficient (CCC) as shown in Eqs. 4–8. MAPE provides a relative measure of accuracy by calculating the average percentage difference between the actual and forecasted values. This metric is a percentage error, and it ranges from ∞ (totally incorrect) to 0% (perfect accuracy). The MSE metric measures the variation between the predicted and observed values, highlighting more significant errors. Higher values of the MSE indicate higher prediction error and vice versa. EV calculates the percentage of the actual values’ variation that the model can account for. This measure ranges from 0 (no variance explained by the model) to 1 (all variation explained by the model). The linear relationship between the actual and predicted outputs is evaluated using Pearson’s correlation. Perfect negative linear relationships are represented by R = − 1; perfect positive linear relationships are represented by R = 1, with 0 denoting no linear relationship. CCC accounts for bias and correlation when assessing the degree of agreement between actual and predicted data. This statistic has a range of 0 (no agreement) to 1 (perfect agreement), where − 1 represents total disagreement.

4 $$MAPE = \frac{1}{n}\mathop \sum \limits_{i = 1}^{n} \left| {\frac{{y_{i} - \hat{y}_{i} }}{{y_{i} }}} \right| \times 100$$

5 $$MSE = \frac{1}{n}\mathop \sum \limits_{i = 1}^{n} \left( {y_{i} - \hat{y}_{i} } \right)^{2}$$

6 $$EV = 1 - \frac{{Var\left( {y_{i} - \hat{y}_{i} } \right)}}{{Var\left( {y_{i} } \right)}}$$

7 $$R = \frac{{\mathop \sum \nolimits_{i = 1}^{n} \left( {y_{i} - \overline{y}} \right)\left( {\hat{y}_{i} - \overline{{\hat{y}}} } \right)}}{{\sqrt {\mathop \sum \nolimits_{i = 1}^{n} \left( {y_{i} - \overline{y}} \right)^{2} \left( {\hat{y}_{i} - \overline{{\hat{y}}} } \right)^{2} } }}$$

8 $$CCC = \frac{{2 \times R \times \sigma_{y} \times \sigma_{{\hat{y}}} }}{{\sigma_{y}^{2} + \sigma_{{\hat{y}}}^{2} + \left( {\mu_{y} - \mu_{{\hat{y}}} } \right)^{2} }}$$

$R$ is the Pearson correlation coefficient, ${\sigma }_{y}$ and ${\sigma }_{\widehat{y}}$ are the standard deviations of the actual and predicted outputs, and ${\mu }_{y}$ and ${\mu }_{\widehat{y}}$ are the means of the actual and predicted outputs.

## Research design and methodology

Developing accurate wind energy forecasting models is essential for optimizing wind power generation and ensuring grid stability. Given the inherent variability of wind, a systematic approach is required to enhance prediction accuracy and reliability. This process involves multiple critical steps:

- Data collection: The initial phase involves gathering time-series data on various meteorological parameters at the Gabal El-Zayt wind farm. The selection of relevant input features is crucial, as it significantly impacts the performance of ML algorithms.
- Data preprocessing: Before training ML models, the collected data undergo preprocessing to enhance quality and suitability. This includes removing outliers and normalizing or transforming data to ensure consistency. In this paper, data normalization was performed to scale the features within the range of [− 1.0 to 1.0], facilitating improved model performance.
- Model development: The next step involves selecting and training ML algorithms using the preprocessed dataset. Both single models, such as MLR and SVM, and ensemble learning models, including RF, ET, DT, BDT, GB, LGBM, XGBoost, and AdaBoost, were utilized. Bayesian optimization was employed to fine-tune hyperparameters, aiming to enhance prediction accuracy and computational efficiency.
- Prediction horizons: The developed models were designed to predict wind energy at various lead times: 10 min (10 M), 30 min (30 M), 6 h (6 H), 24 h (24 H), and 36 h (36 H). This multi-horizon forecasting approach addresses different operational and planning needs within wind energy systems.
- Model evaluation and validation: The final step involves assessing the performance of each ML algorithm to identify the most suitable model for each prediction horizon. Evaluation metrics such as MAPE, MSE, EV, R, and CCC were employed to compare model predictions against actual observations. This rigorous evaluation ensures the selection of optimal algorithms tailored to specific forecasting requirements.

**Fig. 1:**
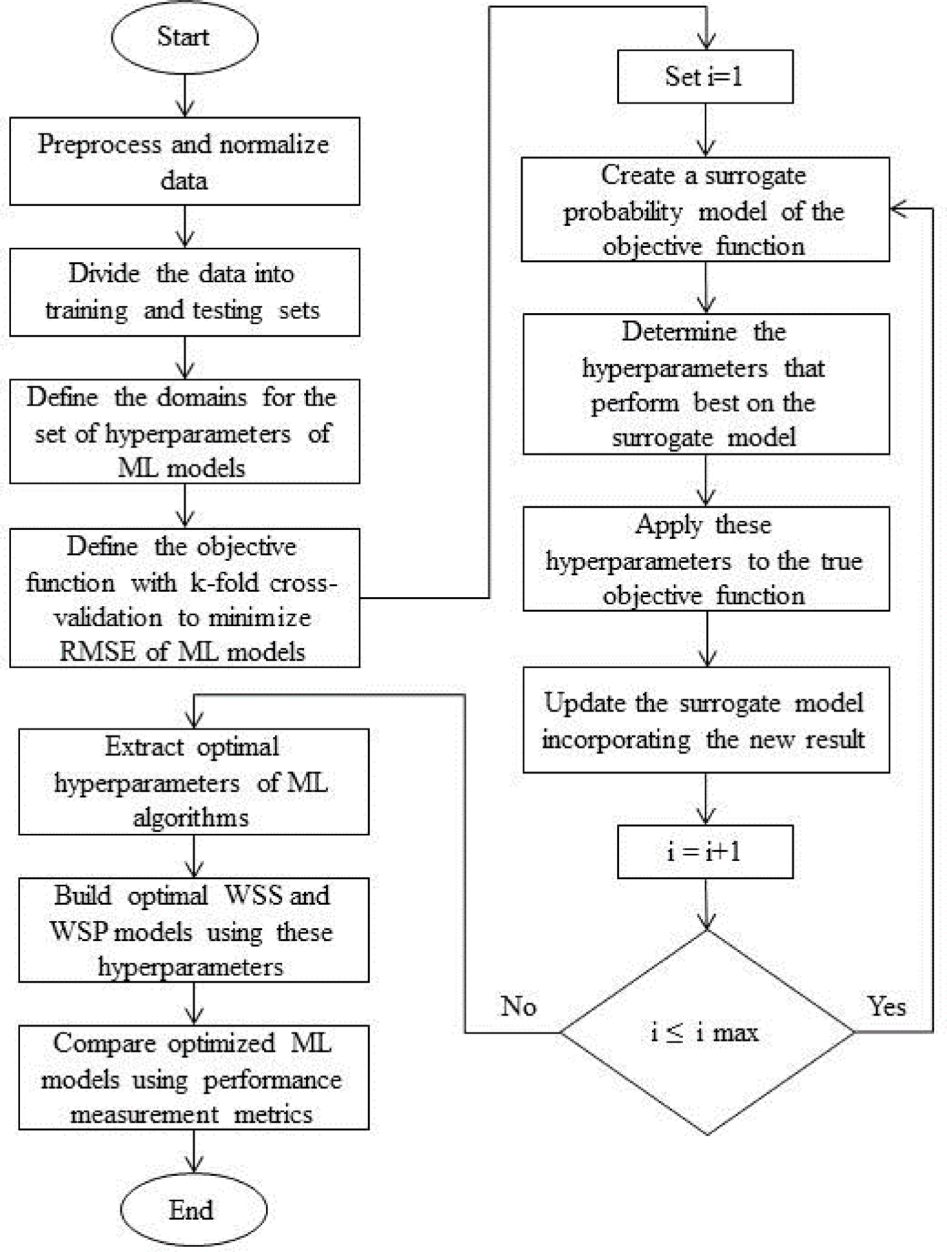
Development of wind speed and power prediction models.

By meticulously following these steps, this paper aims to develop robust ML-based prediction models that enhance the reliability and efficiency of wind speed and power forecasting, thereby supporting the integration of wind energy into the power grid as in Fig. 1.

## Case study and data collection

To achieve meaningful accuracy, both the quantity and quality of data collection are essential^61^. For the generated models, the 200 MW onshore Gabal El-Zayt wind farm has been chosen as the case study. Launched in 2015, the Gabal El-Zayt wind farm in Egypt’s Red Sea governorate was officially opened in 2018. It was the biggest wind farm in Africa and the Middle East when it opened^62^. These factors were used as input data for a WSP model. It is worth mentioning that WPP uses all seven predictors mentioned earlier, plus the wind speed, making a total of eight predictors.

**Fig. 2:**
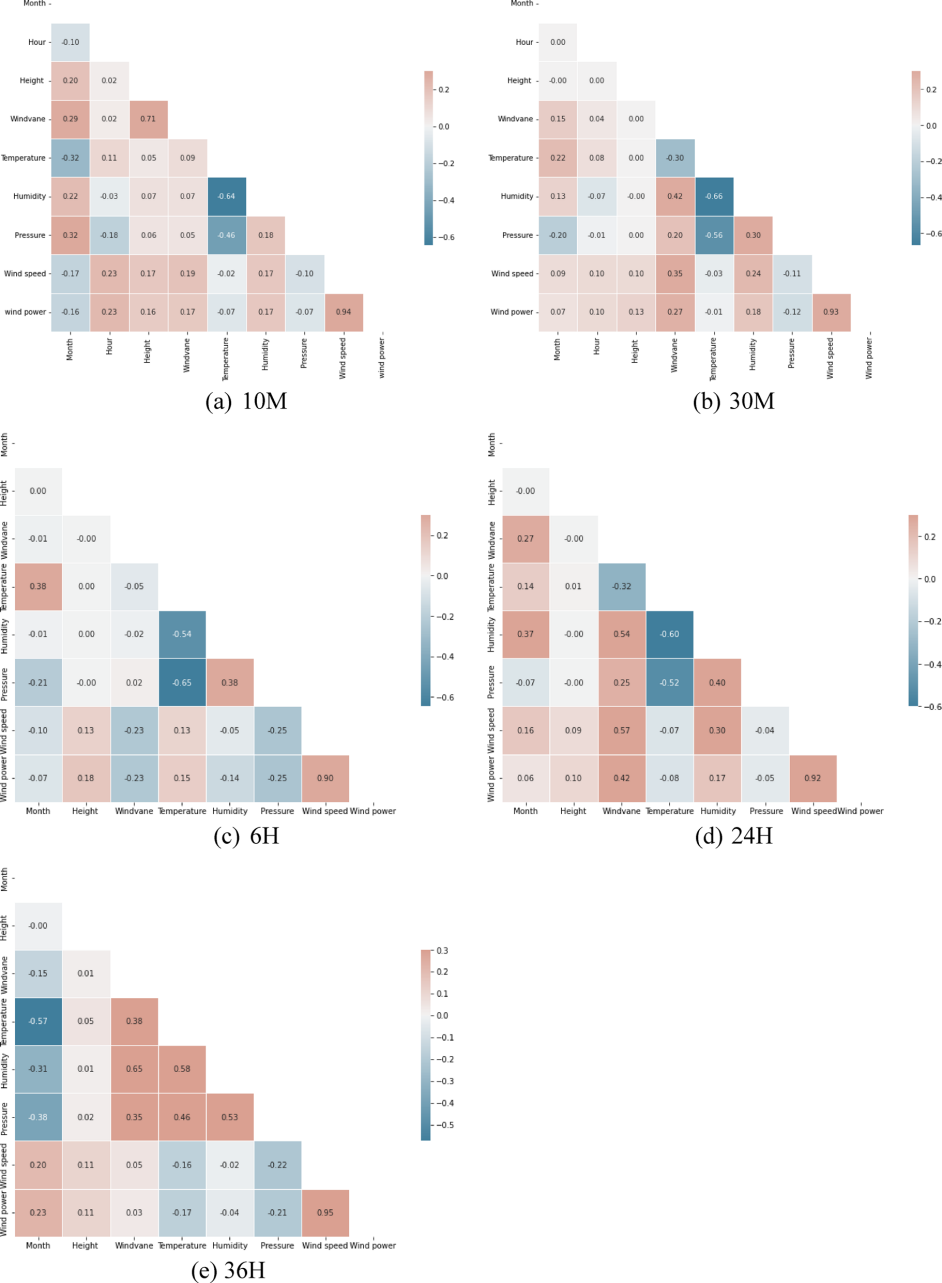
Correlation matrices for 30 min, 6 h, 24 h, and 36 h.

A heat map depicting the correlation between the input factors is shown in Fig. 2, which provides important information about how these factors relate to wind speed and power at different time intervals (i.e., 10 M, 30 M, 6 H, 24 H, and 36 H). The degree of correlation between the respective factors is shown by the value and color of each cell. A positive correlation coefficient denotes a favorable relationship between the factors, whereas a negative correlation value suggests an unfavorable relationship. Analysis of the correlation map reveals that there are several strong correlations between the factors in this dataset. For example, there is a strong positive correlation between the windvane and height (R = 0.71) in the 10 M ahead forecast. Besides, there is a moderate positive correlation of 0.57 between the wind speed and windvane in the 24 H ahead forecast. On the other hand, there are also several weak correlations between the variables. For example, the wind speed and pressure have a weak negative correlation (10 M: R = − 0.10; 30 M: R = − 0.11; 6 H: R = − 0.25; 24 H: R = − 0.04; and 36 H: R = − 0.22). This underscores the intricate natures of wind speed and power, which are influenced by many factors rather than solely dependent on a single factor.

## Results and discussion

### Results and analysis of WSP models

The effectiveness of ML models for WSP and WPP is evaluated over a range of time horizons (10 M, 30 M, 6 H, 24 H, and 36 H). In this study, a comprehensive dataset is initially formulated and divided into two parts: 80% for training and 20% for testing. This division allows for training the models on a substantial portion of the data while still being rigorously tested on an independent dataset to ensure their ge lizability. Bayesian optimization is used for hyperparameter tuning in an iterative and data-driven way based on fivefold cross-validation to further improve the prediction accuracy of ML models. By continually assessing each model’s performance on the testing dataset, this approach not only helps to determine the best set of parameters for each model but also reduces the risk of overfitting. Tables 2 and 3 list the ideal hyperparameters that yield the best results for each ML model. These hyperparameters, which have a substantial impact on the models’ predictive ability, include fit_intercept, kernel, n_estimators, max_depth, and many more. Adjusting these hyperparameters allows for utilizing the entire capability of each model to produce higher performance in wind speed and power predictions at various time intervals.

**Table 2:** Optimal parameters of the Bayesian optimized ML models for predicting wind speed.

Model	Parameters	10 M	30 M	6 H	24 H	36 H
MLR	fit_intercept	True	True	True	True	True
	copy_X	True	True	False	True	False
	Positive	False	False	False	False	False
SVM	Kernel	rbf	rbf	rbf	rbf	rbf
	Gamma	scale	auto	scale	scale	scale
	C	1	1	1	1	1
	Epsilon	0.01	0.1	0.1	1	0.1
ET	n_estimators	400	100	50	100	300
	Criterion	squared_error	friedman_mse	poisson	friedman_mse	friedman_mse
	max_depth	10	None	None	10	None
	min_samples_split	3	4	3	3	3
	min_samples_leaf	3	3	2	2	2
	min_weight_fraction_leaf	0	0	0	0.1	0.1
	max_features	log2	log2	sqrt	auto	auto
	max_leaf_nodes	100	25	50	50	25
RF	n_estimators	100	200	100	500	300
	Criterion	friedman_mse	friedman_mse	squared_error	friedman_mse	absolute_error
	max_depth	5	10	5	None	10
	min_samples_split	2	4	3	4	3
	min_samples_leaf	2	4	2	2	3
	min_weight_fraction_leaf	0	0.2	0.1	0	0.1
	max_features	auto	log2	log2	sqrt	sqrt
	max_leaf_nodes	25	75	None	None	50
DT	Criterion	friedman_mse	friedman_mse	friedman_mse	squared_error	friedman_mse
	Splitter	Best	Best	Best	Best	Best
	max_depth	5	5	5	None	10
	min_samples_split	4	3	3	4	4
	min_samples_leaf	4	2	3	1	2
	min_weight_fraction_leaf	0.1	0.1	0.1	0.1	0
	max_features	sqrt	auto	auto	sqrt	log2
	max_leaf_nodes	100	25	None	25	50
BDT	n_estimators	50	300	50	100	400
	max_samples	1	1	1	1	0.75
	max_features	0.75	1	1	1	0.75
GB	Loss	huber	huber	huber	absolute_error	huber
	learning_rate	0.1	0.1	0.1	1	0.1
	n_estimators	500	200	100	400	100
	Subsample	0.75	0.5	1	0.75	0.75
	Criterion	friedman_mse	squared_error	squared_error	friedman_mse	friedman_mse
	min_samples_split	2	2	3	3	4
	min_samples_leaf	4	3	3	3	1
	min_weight_fraction_leaf	0.1	0.1	0.2	0.2	0.1
	max_depth	10	1	None	10	None
	max_features	log2	log2	auto	sqrt	auto
	max_leaf_nodes	100	25	None	None	None
LGBM	boosting_type	gbdt	dart	gbdt	gbdt	gbdt
	num_leaves	50	40	50	20	50
	max_depth	10	None	None	5	10
	learning_rate	0.092	0.259	0.015	0.5	0.111
	min_child_samples	10	5	10	10	20
	bagging_freq	2	4	3	3	2
	bagging_fraction	0.8	0.6	0.6	0.6	0.7
XGBoost	learning_rate	0.351	0.136	0.511	0.191	0.523
	max_depth	10	10	5	5	5
	reg_lambda	3.335	1.523	3.854	2.369	9.848
	n_estimators	300	300	300	300	400
	min_child_weight	7	2	1	7	4
	Subsample	0.501	0.672	0.737	0.622	0.824
	colsample_bytree	0.773	0.879	0.671	0.658	0.948
AdaBoost	n_estimators	100	200	50	200	100
	learning_rate	1	1	1	1	0.1
	Loss	Linear	Square	Linear	Linear	Exponential

**Table 3:** Optimal parameters of the Bayesian optimized ML models for predicting wind power.

Model	Parameters	10 M	30 M	6 H	24 H	36 H
MLR	fit_intercept	True	True	True	True	True
	copy_X	False	True	False	True	True
	Positive	False	False	False	False	False
SVM	Kernel	rbf	rbf	rbf	Linear	Linear
	Gamma	scale	scale	scale	scale	auto
	C	1	1	1	1	1
	Epsilon	1	0.01	0.1	0.1	1
ET	n_estimators	400	100	300	100	500
	Criterion	squared_error	friedman_mse	poisson	friedman_mse	squared_error
	max_depth	None	5	5	5	10
	min_samples_split	3	3	3	3	3
	min_samples_leaf	4	2	3	2	2
	min_weight_fraction_leaf	0	0	0	0	0.1
	max_features	log2	auto	sqrt	auto	auto
	max_leaf_nodes	50	25	100	None	100
RF	n_estimators	100	300	300	200	100
	Criterion	squared_error	absolute_error	poisson	squared_error	friedman_mse
	max_depth	None	10	None	5	5
	min_samples_split	2	4	2	2	3
	min_samples_leaf	3	3	3	4	3
	min_weight_fraction_leaf	0	0.2	0.1	0	0.1
	max_features	auto	auto	sqrt	sqrt	auto
	max_leaf_nodes	75	50	75	75	25
DT	Criterion	absolute_error	poisson	poisson	absolute_error	friedman_mse
	Splitter	Best	Best	Best	Random	Best
	max_depth	5	10	5	5	10
	min_samples_split	3	3	4	3	4
	min_samples_leaf	1	1	4	2	4
	min_weight_fraction_leaf	0	0.1	0.1	0	0
	max_features	auto	auto	sqrt	auto	log2
	max_leaf_nodes	75	25	75	25	100
BDT	n_estimators	500	300	200	200	400
	max_samples	0.5	0.75	0.75	0.75	1
	max_features	1	1	1	1	1
GB	Loss	squared_error	quantile	huber	huber	absolute_error
	learning_rate	1	0.1	1	0.1	0.1
	n_estimators	100	400	400	300	200
	Subsample	1	0.5	0.5	0.75	0.75
	Criterion	friedman_mse	friedman_mse	friedman_mse	friedman_mse	friedman_mse
	min_samples_split	3	4	3	4	3
	min_samples_leaf	3	3	1	4	2
	min_weight_fraction_leaf	0.1	0.1	0.1	0	0.1
	max_depth	5	1	10	None	1
	max_features	auto	auto	log2	log2	auto
	max_leaf_nodes	75	50	50	50	25
LGBM	boosting_type	rf	dart	rf	gbdt	gbdt
	num_leaves	40	40	40	50	40
	max_depth	5	5	5	10	None
	learning_rate	0.009	0.377	0.027	0.111	0.259
	min_child_samples	20	10	5	5	20
	bagging_freq	4	1	2	3	2
	bagging_fraction	0.9	0.9	0.9	0.7	0.9
XGBoost	learning_rate	0.197	0.068	0.475	0.075	0.077
	max_depth	1	5	5	5	5
	reg_lambda	3.645	6.601	8.807	9.777	9.474
	n_estimators	400	300	200	500	400
	min_child_weight	8	7	7	3	2
	Subsample	0.649	0.968	0.988	0.879	0.566
	colsample_bytree	0.703	0.755	0.719	0.581	0.907
AdaBoost	n_estimators	100	300	500	400	400
	learning_rate	0.1	1	1	1	0.1
	Loss	Exponential	Square	Linear	Square	Square

Table 4 offers a thorough assessment of the effectiveness of several ML models in forecasting VSTWSP (10 M). This evaluation aids in understanding if a model performs well in one metric at the expense of another, which is crucial information for model selection. The MAPE for the MLR model is 46.006%, the MSE is modest at 7.081, the EV is quite low at 0.172, the R is moderate at 0.416, and the CCC is low at 0.304. With a lower MAPE of 36.321%, a lower MSE of 4.318, a noticeably higher EV of 0.498, a higher R of 0.717, and a significantly higher CCC of 0.624, SVM exhibits improved prediction accuracy. However, the ET model has a higher MSE of 8.236 and a comparatively larger MAPE of 52.223%. It reports worse predictive accuracy as evidenced by a lower EV of 0.033, R of 0.395, and CCC of 0.034. With a MAPE of 46.899% and a comparable MSE of 6.450, RF offers performance equivalent to MLR. Besides, it has a modest predictive ability with an EV, R, and CCC of 0.242, 0.574, and 0.295 respectively. With a MAPE of 46.474%, an MSE of 6.982, an EV of 0.179, an R of 0.423, and a CCC of 0.310, DT exhibits outcomes similar to RF. BDT stands out, showing great prediction accuracy with a strikingly low MAPE of 12.741%, a very low MSE of 0.978, a high EV of 0.886, an extraordinarily high CCC of 0.936, and a high R of 0.942. GB has strong predictive performance with a MAPE of 28.380%, MSE of 2.800, EV of 0.671, R of 0.819, and CCC of 0.800. LGBM shows the best predictive ability with the lowest MAPE and MSE values of 12.274% and 0.953 as well as exceptionally high EV of 0.888, R of 0.943, and CCC of 0.939. XGBoost performs quite well in terms of prediction with an EV of 0.867, a MAPE of 14.709%, an MSE of 1.136, an R of 0.931, and a CCC of 0.927. AdaBoost has an EV of 0.480, a MAPE of 34.965%, an MSE of 4.400, an R of 0.699, and a CCC of 0.618, showing a decent but not exceptional level of prediction accuracy. In conclusion, the LGBM, BDT, and XGBoost stand out as good performers, whereas the ET, MLR, and DT models exhibit somewhat lower prediction accuracy.

**Table 4:** Results of ML models for VSTWSP (10 M).

VSTWSP model	MAPE (%)	MSE	EV	R	CCC
MLR	46.006	7.081	0.172	0.416	0.304
SVM	36.321	4.318	0.498	0.717	0.624
ET	52.223	8.236	0.033	0.395	0.034
RF	46.899	6.450	0.242	0.574	0.295
DT	46.474	6.982	0.179	0.423	0.310
BDT	12.741	0.978	0.886	0.942	0.936
GB	28.380	2.800	0.671	0.819	0.800
LGBM	**12.274**	**0.953**	**0.888**	**0.943**	**0.939**
XGBoost	14.709	1.136	0.867	0.931	0.927
AdaBoost	34.965	4.400	0.480	0.699	0.618

Optimal values are in bold.

**Fig. 3:**
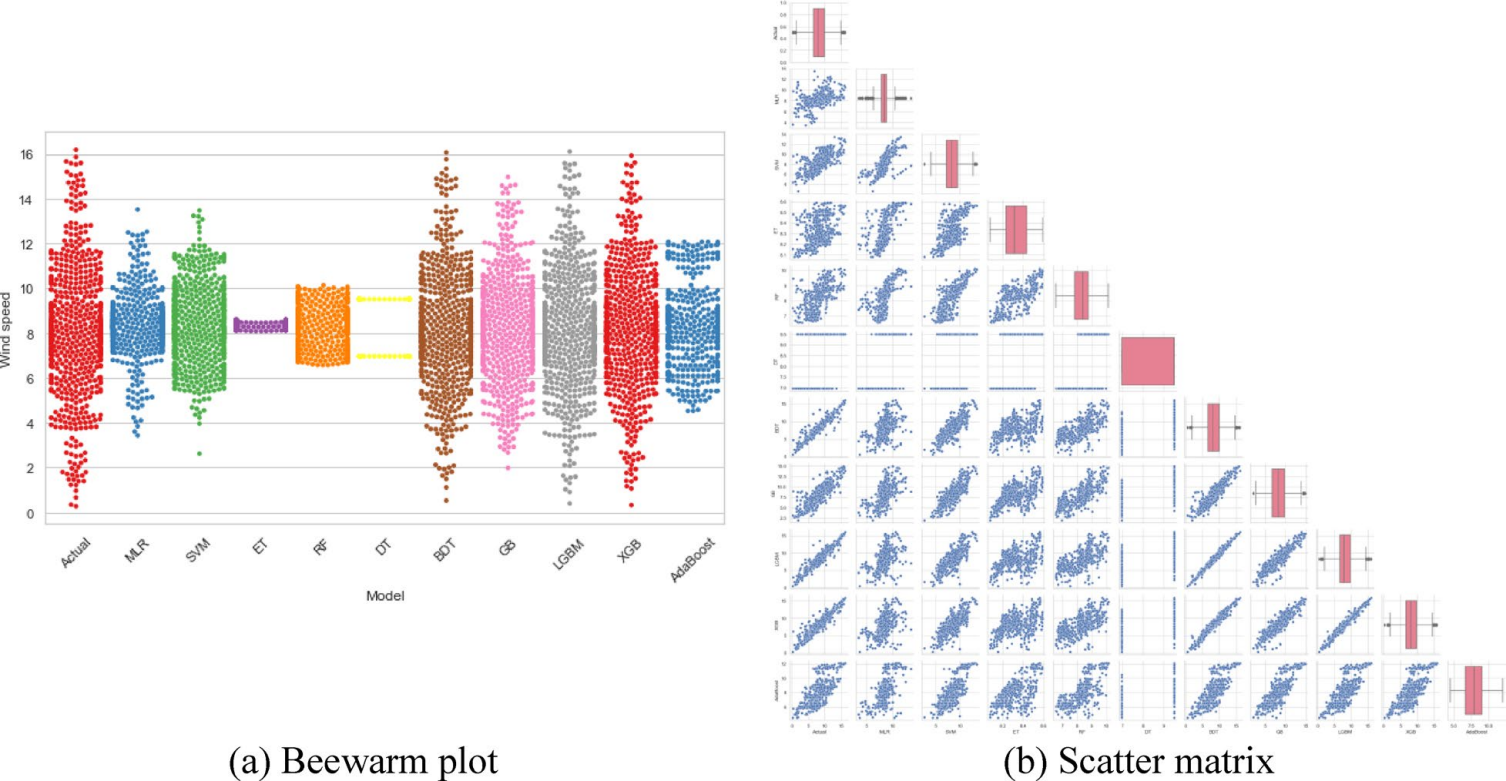
Beeswarm plot and scatter matrix of ML models for VSTWSP (10 M).

To facilitate a more comprehensive understanding of the findings, multiple graphs are plotted to showcase different perspectives on the effectiveness of VSTWSP models. These visual representations provide useful information for selecting the best model based on its unique features and performance requirements. A visual illustration of the effectiveness of multiple ML models for the 10-min VSTWSP is provided in Fig. 3.

The beeswarm plot provides a detailed distribution of each model’s performance along the y-axis, visually representing the spread and dispersion of predictive accuracy. Models that closely mimic the actual data distribution indicate stronger performance, while greater deviations suggest lower accuracy. The scatter matrix, on the other hand, enables a deeper examination of the relationships and trade-offs among the developed models. By analyzing agreement levels and potential biases, this visualization offers valuable insights into model correlations. Additionally, the diagonal boxplots in the scatter matrix help identify variations in central tendencies and outliers, offering a more nuanced perspective on model performance.

The analysis of Fig. 3 reveals that LGBM, BDT, and XGBoost continually stand out as the top performers, as evidenced by the same clusters in the beeswarm plot and noteworthy correlation patterns in the scatter matrix. In contrast, the beeswarm plot’s deviation from the actual model and the scatter matrix’s poorer correlations show that the ET and DT models have considerably lower predictive accuracy.

Online Appendix B presents the line plot, scatter plot with error histogram, residual plot, and quantile–quantile (Q–Q) plot of Bayesian optimized models. The line plot depicts the agreement level between actual and predicted outputs. Predicted vs actual values are also displayed as a scatter plot with an error histogram, which illustrates the distribution of prediction errors. This plot aids in evaluating the correlation between predicted and actual results as well as the dispersion of forecast errors. The difference between the actual and predicted values, or residuals, is shown against a reference line in the residual plot. This plot supports the evaluation of the linearity and homoscedasticity (constant variance) of errors. The quantiles of the actual and anticipated value distributions are compared using a Q–Q plot, which is employed to evaluate the residuals’ normality.

It is clear from examining these plots that actual values and the predictions of the LGBM model are closely aligned. Additionally, the points in this model are more closely centered around the diagonal line, suggesting few outliers and accurate predictions. More concentrated error distributions are observable around zero in the error histogram, but there is a rightward-pointing error tail. The residuals are closely dispersed around the reference line in the residual plot, while the reference line and the points in the Q–Q plot are in alignment. In many statistical methods, it is suggested that the residuals conform to a normal distribution, and a robust alignment in the Q–Q plot suggests that they adhere to this characteristic. Overall, the figures show that the LGBM model is a good predictor of the VSTWSP outcomes. The remaining models can be interpreted similarly.

By investigating scatter plots and residual plots, BDT and XGBoost provide closely clustered points and homoscedastic residuals, which demonstrate accurate forecasts and reliable performance. In the Q–Q plot, these models show high alignment with the reference line, indicating that the residuals of these models have a normal distribution. Furthermore, there is a quite good alignment between actual and forecasted values for GB, AdaBoost, and SVM. Their residuals have wider distributions but are generally homoscedastic and normal. In contrast, more dispersion from actual values is shown in ET, RF, DT, and MLR, indicating worse forecast accuracy. These models exhibit non-normal residual distributions and heteroscedastic residuals with variable error spreads, suggesting less consistent performance and possible problems with certain data points.

Table 5 depicts the ML results based on several prediction horizons (30 min, 6 h, 24 h, and 36 h) ahead of WSP. These outcomes demonstrate the models’ performance in terms of MAPE, MSE, EV, R, and CCC. For the 30-min prediction horizon, XGBoost has substantially reduced MAPE and MSE, enhancing its predictive accuracy in comparison to other models. Its EV value is much higher at a value of 0.988, indicating that it accounts for a greater share of the variance in the data. Additionally, CCC and R values of 0.994 show a high level of agreement and a strong linear relationship with the actual data. On the other hand, ET, RF, and DT models have larger MAPE and MSE, suggesting less accurate predictions, with their errors almost surpassing other models. Significantly outperforming these models are the BDT and LGBM models, with MAPE improvements varying from 85 to 91%. These models consistently show higher values for EV, R, and CCC, highlighting their propensity to capture a bigger proportion of the variation in the data, preserve robust linear relationships, and demonstrate a better degree of agreement with the actual data.

**Table 5:** ML results for 30 min, 6 h, 24 h, and 36 h ahead of WSP.

ML models	MAPE (%)	MSE	EV	R	CCC	MAPE (%)	MSE	EV	R	CCC
	30 M					6 H				
MLR	19.205	3.661	0.482	0.694	0.647	50.767	9.653	0.167	0.413	0.156
SVM	17.407	2.798	0.606	0.790	0.723	50.100	4.316	0.290	0.557	0.394
ET	30.738	6.515	0.084	0.453	0.093	55.657	5.434	0.107	0.465	0.123
RF	24.711	4.799	0.328	0.626	0.407	9.878	0.687	0.887	0.946	0.935
DT	24.969	4.553	0.357	0.598	0.517	55.422	5.467	0.102	0.330	0.155
BDT	2.667	0.118	0.983	0.992	0.991	**4.294**	**0.186**	**0.969**	**0.985**	**0.984**
GB	16.918	2.533	0.642	0.818	0.748	47.233	4.200	0.309	0.570	0.423
LGBM	3.851	0.201	0.976	0.989	0.985	24.322	1.593	0.739	0.914	0.808
XGBoost	**2.641**	**0.087**	**0.988**	**0.994**	**0.994**	4.581	0.222	0.964	0.982	0.981
AdaBoost	15.550	1.739	0.754	0.872	0.849	40.782	3.522	0.422	0.655	0.565

ML models	MAPE (%)	MSE	EV	R	CCC	MAPE (%)	MSE	EV	R	CCC
	24 H					36 H				
MLR	26.034	4.155	0.414	0.644	0.600	40.707	8.426	0.097	0.312	0.172
SVM	22.424	3.458	0.514	0.717	0.679	37.167	7.666	0.173	0.416	0.285
ET	32.316	4.880	0.319	0.657	0.380	28.422	3.591	0.594	0.851	0.672
RF	17.136	1.503	0.794	0.891	0.880	17.069	1.615	0.817	0.911	0.888
DT	25.193	3.497	0.515	0.718	0.684	38.357	5.764	0.348	0.591	0.531
BDT	3.311	0.069	0.990	0.996	0.995	7.268	0.270	0.970	0.986	0.984
GB	16.756	1.727	0.758	0.871	0.865	25.139	2.995	0.661	0.816	0.781
LGBM	4.414	0.118	0.983	0.992	0.991	11.504	0.673	0.924	0.962	0.959
XGBoost	**2.663**	**0.044**	**0.994**	**0.997**	**0.997**	**4.943**	**0.137**	**0.985**	**0.992**	**0.992**
AdaBoost	22.031	2.034	0.727	0.860	0.818	37.024	5.592	0.367	0.634	0.472

Optimal values are in bold.

Similar trends continue when the forecast horizon is increased to six hours. In comparison to the other models, BDT performs better with reduced MAPE and MSE and improved EV, R, and CCC metrics, demonstrating their reliable prediction accuracy. However, ET, MLR, and DT models continue to be less accurate, with their MAPE and MSE errors being around 12–13 and 29–52 times higher than those of the BDT model. Additionally, the EV, R, and CCC values of these models remain lower, suggesting less accuracy in identifying data patterns. Notably, XGBoost (MAPE = 4.581%, MSE = 0.222, EV = 0.964, R = 0.982, and CCC = 0.981) and RF (MAPE = 9.878%, MSE = 0.687, EV = 0.887, R = 0.946, and CCC = 0.935) models outperform the above-mentioned models. They consistently outperform these models in terms of prediction accuracy, which is supported by their satisfactory performance metrics.

The patterns in model performance persist for the 24-h and 36-h forecast horizons. In comparison to the other models, XGBoost continues to perform better with MAPE = 2.663%, MSE = 0.044, EV = 0.994, R = 0.997, and CCC = 0.997 for 24-h and MAPE = 4.943%, MSE = 0.137, EV = 0.985, R = 0.992, and CCC = 0.992 for 36-h ahead prediction of wind speed. These evaluation metrics demonstrate the robust outperformance of this model in identifying data patterns. On the other side, ET and MLR models are associated with higher values of MAPE and MSE and lower values of EV, R, and CCC metrics. At the same time, BDT and LGBM models keep improving significantly in comparison to ET and MLR models, with MAPE and MSE reduction ranging from 83 to 90% and 97 to 99%, respectively. These findings highlight the large improvements of XGBoost and BDT models in prediction accuracy across all time frames.

**Fig. 4:**
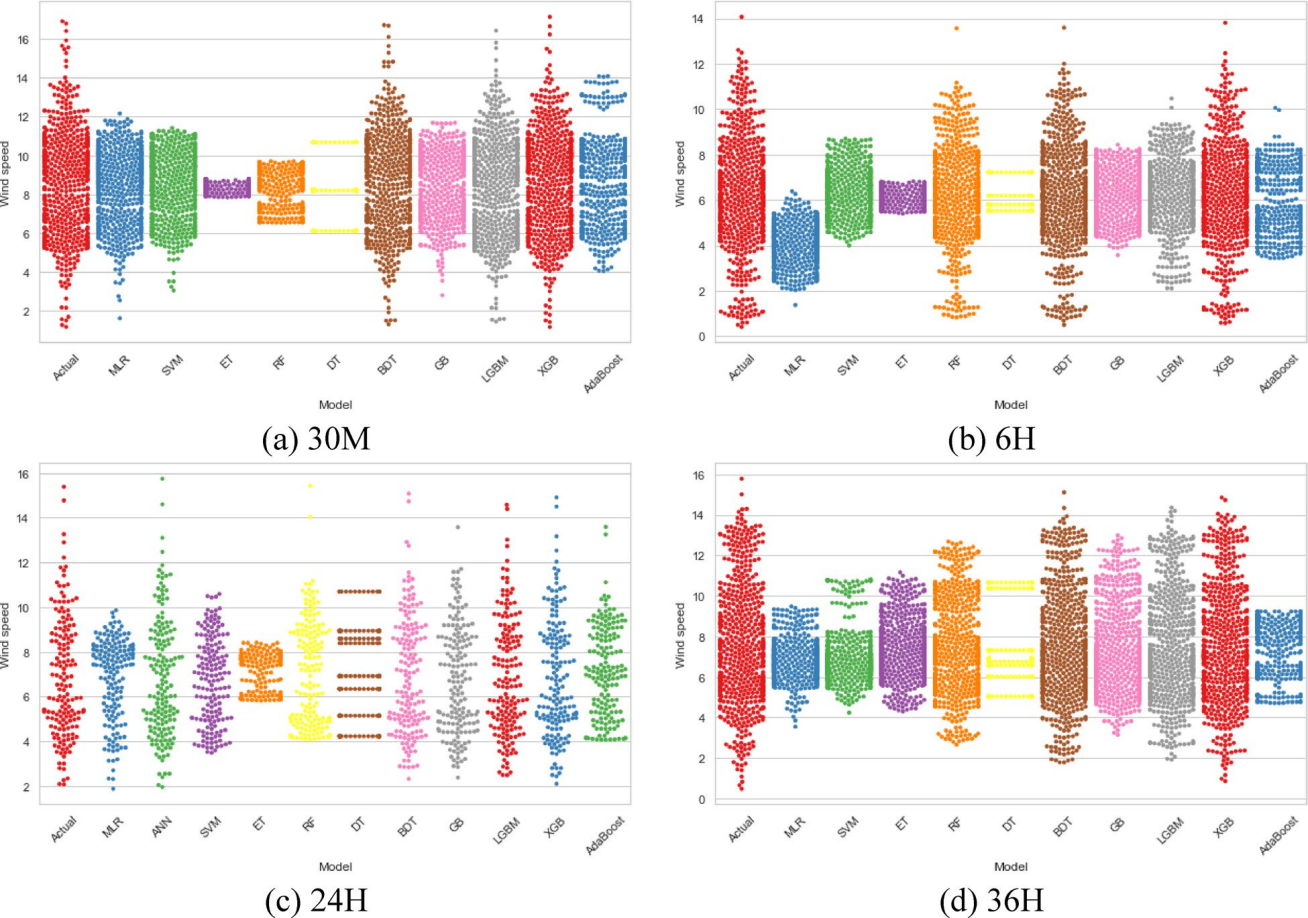
Beeswarm plot for 30 min, 6 h, 24 h, and 36 h ahead of WSP.

**Fig. 5:**
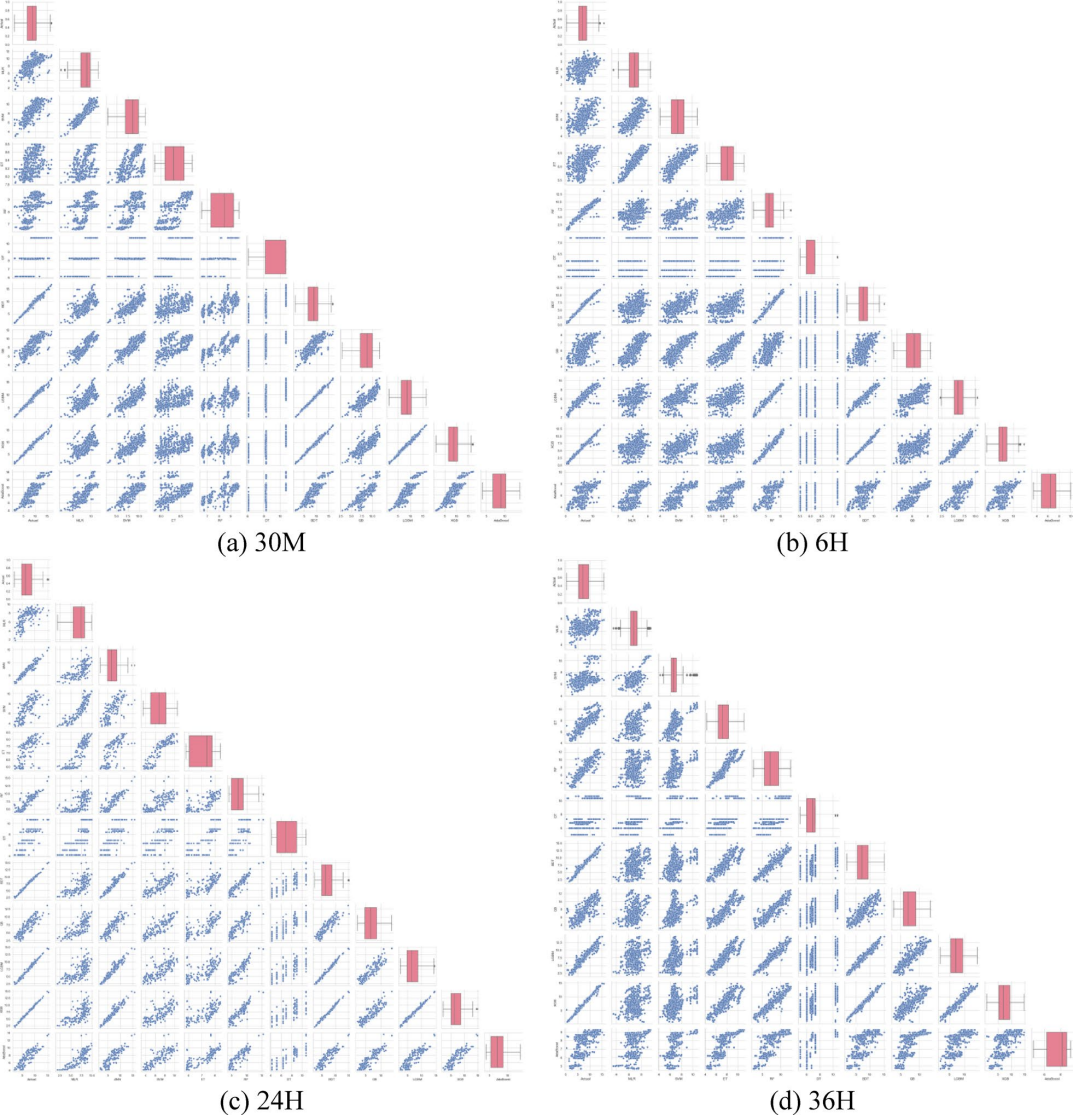
Scatter matrix for 30 min, 6 h, 24 h, and 36 h ahead of WSP.

For visual representations of the developed models, the beeswarm graphic and the scatter matrix are plotted in Figs. 4 and 5 to examine the clustering and alignment of points along the diagonal. These charts show how each model performs and how its relationships change for various prediction horizons. Figure 4 illustrates the beeswarm plot for 30 min, 6 h, 24 h, and 36 h ahead of WSP. The distribution of model predictions for the 30-min prediction horizon is shown in the first plot. It demonstrates that XGBoost performs better than other models by displaying how closely its predictions cluster with the actual values. The performance of the developed models is displayed in the second plot when the forecast horizon is extended to six hours. It visually illustrates how BDT has enhanced performance by making more accurate forecasts. A visual comparison of model performance across long prediction horizons is provided by the third and fourth plots. As seen by the near forecasts to the actual values, XGBoost is still the better option for these timeframes.

Scatter matrices are useful for evaluating the relationships between multiple variables. For the 30-min prediction horizon, this plot can be used to support the XGBoost’s strong linear relationship (R = 0.994) with the actual data. It is therefore expected to see tight clusters along the diagonal line of scatter plots for XGBoost, indicating strong correlations with the actual data. Similar to the previous scatter matrix, the second matrix can help visualize the relationships between predictions and actual values when the prediction horizon is extended to 6 h. It is aligned with the assertion that the BDT model outperforms others, as evidenced by a strong linear relationship for this model. Finally, the last two scatter matrices reveal strong linear relationships or high R values of 0.997 and 0.992 for predicting 24 h and 36 h ahead using the XGBoost model.

### Results and analysis of WPP models

A comprehensive overview of the findings from multiple ML models for VSTWPP (10 M) can be seen in Table 6. Notably, compared to MLR and SVM, the ET, RF, DT, BDT, GB, LGBM, and XGBoost models show reduced MAPE and MSE, suggesting greater prediction accuracy. These models exhibit high CCC with the actual data, preserve robust linear connections (R), and capture a sizeable percentage of the data variation. On the other hand, MLR and SVM models have larger MAPE (8135.878% and 6365.662%) and MSE (1579536.189 and 220440.296), suggesting less accurate predictions. MLR has a good degree of EV and R, but its CCC is relatively low (i.e., 0.262), indicating poor accuracy in identifying data patterns. With lower EV and R values than MLR, SVM still exhibits considerable prediction accuracy while decreasing MAPE and MSE.

**Table 6:** Results of ML models for VSTWPP (10 M).

VSTWPP model	MAPE (%)	MSE	EV	R	CCC
MLR	8135.878	1579536.189	0.873	0.935	0.262
SVM	6365.662	220440.296	0.341	0.925	0.351
ET	5051.204	69668.005	0.780	0.961	0.833
RF	1613.247	10508.145	0.966	0.983	0.982
DT	1409.690	12035.016	0.962	0.981	0.980
BDT	**12.253**	9695.981	0.969	0.984	0.984
GB	96.148	10016.631	0.968	0.984	0.984
LGBM	186.710	**9444.576**	**0.970**	**0.985**	**0.985**
XGBoost	128.248	9689.123	0.969	0.984	0.984
AdaBoost	1648.051	9816.666	0.968	0.984	0.984

Optimal values are in bold.

Online Appendix C provides the ML outcomes for WPP. The outcomes produced by different ML models when applied to VSTWPP (10 M) are visually represented by the beeswarm plot and scatter matrix in Fig. C1. These visual representations offer a comprehensive overview of the results produced by the developed models, supporting the performance evaluation metrics highlighted in the textual analysis (Table 6). Notably, output patterns for the LGBM, BDT, GB, and XGBoost models can be seen in the beeswarm plot, supporting the finding that these models have higher predictive accuracy. Furthermore, the scatter matrix illustrates that the points are closely clustered around the diagonal line, indicating the alignment of predictions with the actual values and low error values. Besides, the boxplots for these models are compact, suggesting a narrow spread of the residuals and affirming accurate predictions. In summary, the scatter plot with diagonal boxplot provides insights into the correlations between various outputs, reinforcing the idea that these models not only achieve reduced error metrics but also exhibit strong linear relationships (R) and a high degree of agreement (CCC) with the actual data.

The effectiveness of several WPP models is shown in Table 7 for prediction horizons of 30 min, 6 h, 24 h, and 36 h. This table offers insightful information about the models’ propensity for predicting wind power generation at various time periods. At the 30-min prediction horizon, BDT, LGBM, and XGBoost have the highest predictive accuracy followed by AdaBoost, whereas SVM, DT, ET, and MLR have lower predictive accuracy.

**Table 7:** ML results for 30 min, 6 h, 24 h, and 36 h ahead of WPP.

WPP model	MAPE (%)	MSE	EV	R	CCC	MAPE (%)	MSE	EV	R	CCC
	30 M					6 H				
MLR	5504.135	35569.572	0.845	0.920	0.914	3307.693	429898.749	0.826	0.912	0.356
SVM	8479.034	162353.023	0.301	0.770	0.333	1166.166	127138.609	0.283	0.856	0.279
ET	1379.787	50317.086	0.782	0.938	0.842	2831.659	120943.495	0.226	0.816	0.242
RF	68.354	12871.690	0.944	0.973	0.970	339.914	3922.046	0.975	0.992	0.986
DT	1402.914	83294.928	0.637	0.800	0.790	1749.889	49917.394	0.680	0.830	0.791
BDT	**0.401**	**0.927**	**1.000**	**1.000**	**1.000**	**0.277**	**17.439**	**1.000**	**1.000**	**1.000**
GB	140.231	14388.165	0.945	0.986	0.973	443.296	29532.064	0.812	0.903	0.889
LGBM	8.523	59.313	1.000	1.000	1.000	44.615	178.750	0.999	0.999	0.999
XGBoost	13.826	71.010	1.000	1.000	1.000	23.368	276.658	0.998	0.999	0.999
AdaBoost	866.649	293.159	0.999	0.999	0.999	686.042	823.725	0.995	0.997	0.997

WPP model	MAPE (%)	MSE	EV	R	CCC	MAPE (%)	MSE	EV	R	CCC
	24 H					36 H				
MLR	138.996	45039.082	0.874	0.935	0.933	2063.651	34837.503	0.909	0.954	0.953
SVM	190.587	341791.381	0.103	0.651	0.102	1074.062	357303.269	0.217	0.812	0.204
ET	106.907	105988.270	0.702	0.878	0.783	391.335	20149.401	0.948	0.992	0.968
RF	104.411	17545.705	0.951	0.985	0.971	352.596	30129.451	0.922	0.960	0.958
DT	56.879	29095.181	0.918	0.958	0.957	148.219	8725.455	0.977	0.989	0.989
BDT	**0.665**	363.314	0.999	0.999	0.999	**0.888**	**1.529**	**1.000**	**1.000**	**1.000**
GB	34.017	7848.280	0.978	0.991	0.988	123.055	435.061	0.999	0.999	0.999
LGBM	1.342	**323.531**	**0.999**	**1.000**	**1.000**	4.676	24.010	1.000	1.000	1.000
XGBoost	5.508	661.862	0.998	0.999	0.999	65.177	271.285	0.999	1.000	1.000
AdaBoost	29.609	1262.688	0.997	0.998	0.998	233.283	896.580	0.998	0.999	0.999

Optimal values are in bold.

At the 6-h prediction horizon, BDT yields a remarkably low MAPE of 0.277%, a low MSE of 17.439, and an outstanding EV, R, and CCC of 1.000, demonstrating its exceptional predictive accuracy. With minimal error metrics and outstanding performance metrics (EV, R, and CCC = 0.999), LGBM and XGBoost models also perform well. However, ET and MLR models yield fewer accurate forecasts as they are associated with lower EV, R, and CCC values and greater MAPE and MSE values.

At the 24-h prediction horizon, LGBM has remarkable predictive accuracy, with a minimal MAPE and MSE of 1.342% and 323.531. It reaches perfect values for EV, R, and CCC, demonstrating an excellent capacity to capture data variance, maintain stable linear relationships, and attain total agreement with the actual data. GB, XGBoost, and AdaBoost also perform well with near-perfect performance metrics (EV, R, and CCC at 0.999), demonstrating their effectiveness in wind power forecasting. However, it is difficult for ET, RF, and MLR to retain strong linear correlations, agreement with the real data, and capture a large percentage of the variation in the data.

The BDT stands out as a top performer at the 36-h prediction horizon, attaining the lowest MAPE (0.888%), lowest MSE (1.529), and perfect EV, R, and CCC (1.000). The LGBM, XGBoost, and GB models work well while exhibiting minimal error metrics and remarkable performance metrics (EV, R, and CCC at 1.000). On the other hand, SVM and MLR exhibit poor accurate predictions with larger MAPE and MSE values. Their lower EV, R, and CCC values further illustrate the difficulties in identifying data patterns and maintaining robust linear correlations and agreement with the actual data.

The beeswarm graphic and the scatter matrix are presented in Figs. C2–C3 to visually illustrate the developed models over a range of prediction horizons. The beeswarm plot for 30 min, 6 h, 24 h, and 36 h ahead of WPP is shown in Fig. C2. The first graphic displays the distribution of model predictions for the 30-min prediction horizon. It illustrates how well BDT’s predictions resemble the actual values, proving its outperformance to other models. When the prediction horizon is extended to six hours, the performance of the assessed models is shown in the second plot. This plot graphically displays how BDT has improved performance by producing more precise forecasts. A visual comparison of model performance across long prediction horizons is provided by the third and fourth plots. As seen by the close forecasts to the actual values, LGBM and BDT yield the best outcomes for these timeframes.

Scatter matrices (Fig. C3) are useful for evaluating the relationships between multiple variables. For the 30-min prediction horizon, this plot can be used to support the BDT’s strong linear relationship (R = 1.000) with the actual data. It is therefore expected to see tight clusters along the diagonal line of scatter plots for BDT, indicating strong correlations with the actual data. Similar to the previous scatter matrix, the second matrix can help visualize the relationships between predictions and actual values when the prediction horizon is extended to 6 h. It is aligned with the assertion that the BDT model outperforms others, as evidenced by a strong linear relationship for this model. Finally, the last two scatter matrices reveal strong linear relationships or high R values of 1.000 for predicting 24 h and 36 h ahead using the LGBM and BDT models.

### Computational memory usage of ML models

**Fig. 6:**
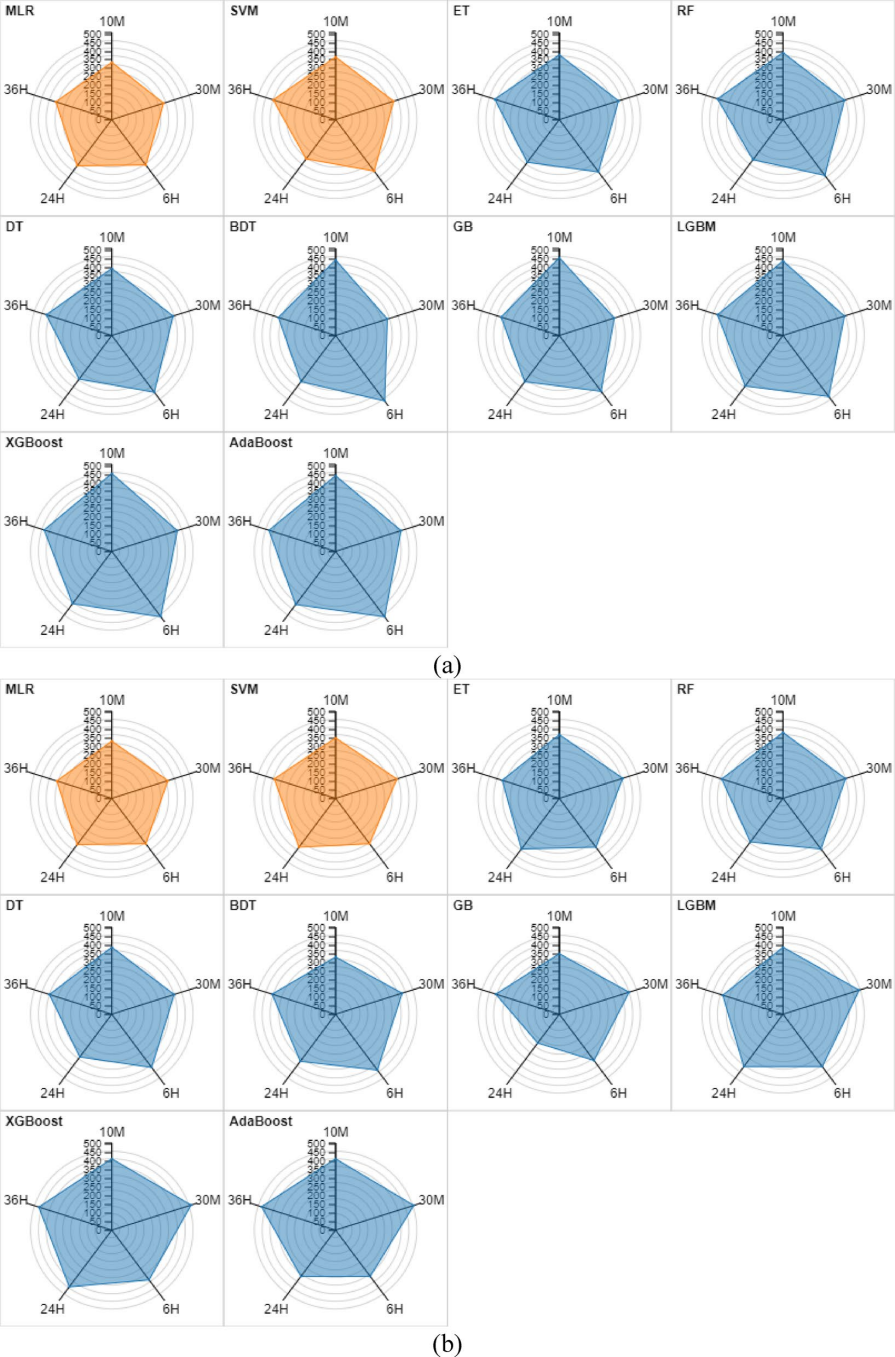
Average computational memory usage for (**a**) WSP and (**b**) WPP.

The memory usage for WSP using ML models across various prediction horizons is shown in Fig. 6a. It is essential to comprehend these memory needs when selecting a suitable model for WSP. This evaluation signifies the trade-offs between memory usage and prediction accuracy, enabling more informed model selection. Notably, MLR uses memory most effectively, using just 361.629 MB for the 10-min prediction horizon. The memory usage of the GB and XGBoost is significantly greater at 489.715 MB and 493.805 MB, respectively. As the forecast horizon gets longer, there exist variations with respect to the memory usage trends. While some models, like MLR, maintain a comparatively consistent memory usage, certain other models including BDT exhibit variances in memory usage.

Memory usage for estimating wind power using several ML models and across prediction horizons is shown in Fig. 6b. MLR and BDT models exhibit more efficient memory usage at the 10-min prediction horizon; MLR uses around 359.055 MB while BDT uses about 362.543 MB. As the prediction horizon is extended to 30 min, memory use for SVM and ET marginally increases with SVM consuming 398.379 MB and ET using 405.930 MB. In the meanwhile, MLR exhibits consistent trends of memory usage and keeps memory consumption fairly constant. GB demonstrates a significant reduction in memory usage (i.e., 364.586 MB) for the 6-h projection horizon. On the other hand, DT exhibits an increase in memory usage to 417.766 MB. XGBoost and AdaBoost show greater memory consumption than the other models with memory usage values of 440.125 MB and 475.840 MB at the 24-h and 36-h prediction horizons, respectively.

### Computational time comparison of ML models

**Fig. 7:**
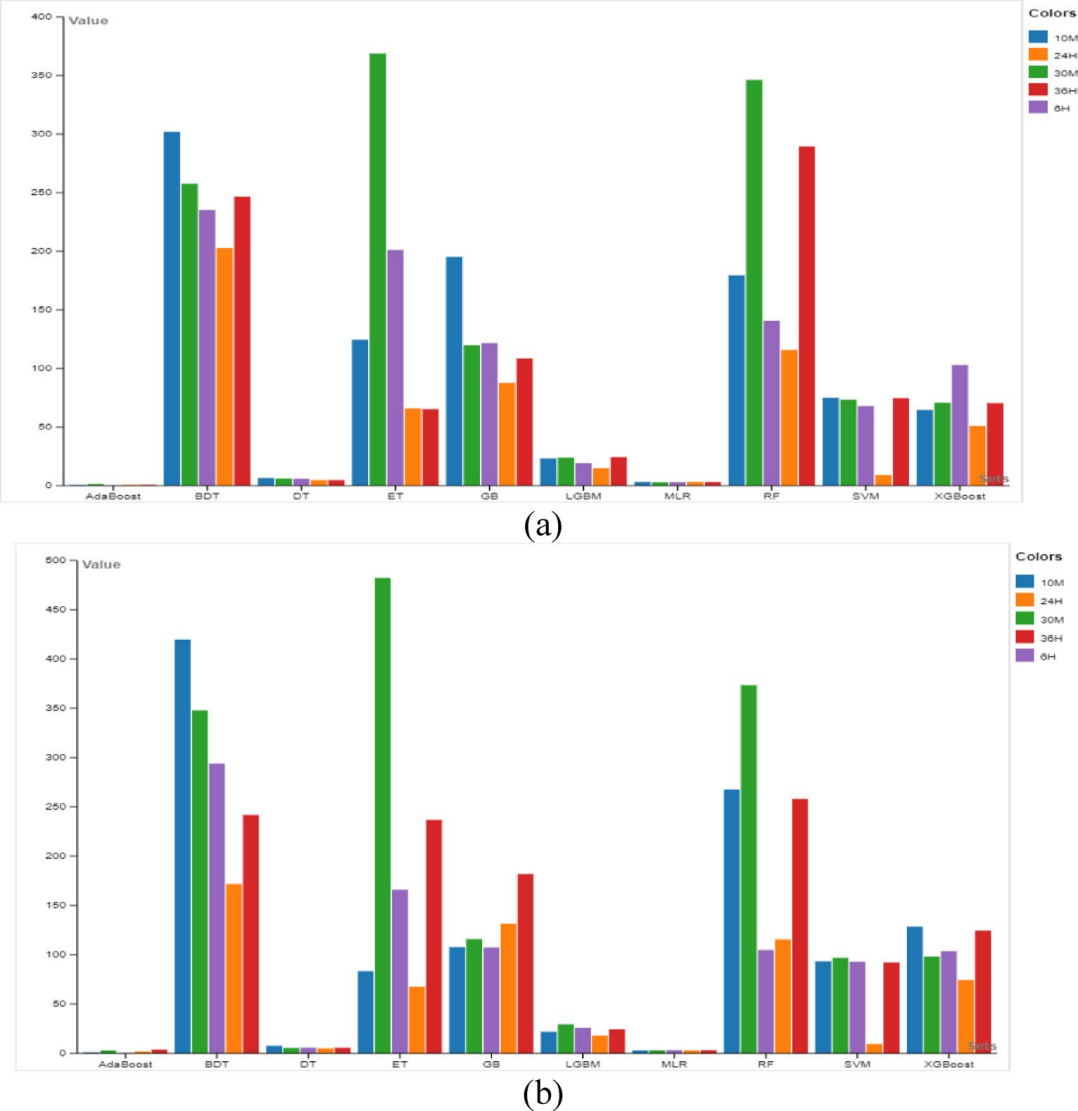
Average computational time for (**a**) WSP and (**b**) WPP.

The execution time for predicting wind speed using different ML models is shown in Fig. 7a. It is crucial to comprehend these variances in execution timeframes when choosing the best ML model and setting up the system for wind speed forecasting tasks. This evaluation allows for choosing a model by highlighting the trade-offs between prediction speed and model accuracy. AdaBoost is the quickest model for the 10-min prediction horizon, completing predictions in just 0.781 s, which makes it stand out with incredibly short execution times. However, SVM exhibits a very long execution time when the prediction horizon increases, particularly at 30 min and 6 h, where it takes 73.439 and 67.98 s, respectively, to complete predictions. Moreover, LGBM is one of the fastest for the 6-h timeframe, finishing predictions in 19.234 s. BDT takes the longest time to finish predictions for the 24-h and 36-h prediction horizons, with execution durations ranging from 202.840 to 246.652 s. Nevertheless, despite a modest increase in execution time, AdaBoost continues to be the quickest model for all prediction horizons.

The execution time for predicting wind power using different ML models across different prediction horizons is shown in Fig. 7b. AdaBoost comes out as the quickest model at the 10-min prediction horizon, with an execution time of just 1.016 s. On the other hand, SVM has one of the slowest execution times (i.e., 93.363 s) for this timeframe. SVM continues to have noticeably long execution times, requiring 96.973 s to finish predictions when the prediction horizon is extended to 30 min. On the other hand, AdaBoost continues to run the quickest model, showcasing the advantages of its effectiveness even when the prediction horizon grows. Execution speeds differ amongst models for the 6-h prediction horizon, with LGBM being one of the quickest, finishing predictions in 25.898 s. On the other hand, BDT and ET have longer execution times, being slow for this timeframe. BDT and RF take the longest to finish predictions at the 24-h and 36-h prediction horizons, with execution durations ranging from 171.825 to 258.135 s. Contrarily, despite a modest increase in execution time, AdaBoost continues to be the quickest model.

### Comparison and discussion

The 10 created models are evaluated using MAPE, MSE, EV, R, and CCC, as shown in Tables 8 and 9. Evaluating the constructed ML models’ efficacy, advantages, and disadvantages is the aim. Table 8 summarizes the efficacy of the established machine learning models for WSP at different time scales. While the ET, RF, and MLR models produce the most erroneous forecasts across all time scales, the XGBoost and BDT models perform exceptionally well for various periods.

**Table 8:** Performance comparison for different ML models for WSP.

ML models	10 M	30 M	6 H	24 H	36 H
MLR	A	A	A	I	I
SVM	G	G	A	A	I
ET	I	I	I	I	G
RF	I	I	E	G	G
DT	A	A	I	A	A
BDT	E	E	E*	E	E
GB	G	G	G	G	G
LGBM	E*	E	G	E	E
XGBoost	E	E*	E	E*	E*
AdaBoost	G	G	G	G	A

E*, Optimal algorithm; E, Excellent; G, Good; A, Acceptable; I, Inaccurate.

**Table 9:** Performance comparison for different ML models for WPP.

ML models	10 M	30 M	6 H	24 H	36 H
MLR	I	A	A	A	I
SVM	I	I	I	I	I
ET	A	A	I	I	A
RF	G	G	G	G	A
DT	A	I	A	A	G
BDT	E	E*	E*	E	E*
GB	E	G	A	G	G
LGBM	E*	E	E	E*	E
XGBoost	E	E	E	E	E
AdaBoost	G	G	G	G	G

E*, Optimal algorithm; E, Excellent; G, Good; A, Acceptable; I, Inaccurate.

Table 9 summarizes how the established ML models performed for predicting wind power at various time frames. BDT, LGBM, and XGBoost models have excellent performance for different time scales, whereas the RF and AdaBoost models have exhibited good performance. Nevertheless, these models’ applicability is contingent upon the particular period of the wind power prediction task. Meanwhile, AdaBoost and RF perform well and consistently as well, making them valuable alternatives for various predicting horizons. It is therefore crucial to select the model that best fits the unique prediction requirements for the specified time scale.

Table 10 summarizes the developed ten ML algorithms. The strengths, weaknesses, interpretability, missing values handling, accuracy, and complexity of each algorithm are mentioned. For example, XGBoost and LGBM performed exceptionally well due to their ability to handle large datasets with complex patterns efficiently. These models leverage gradient boosting, which sequentially corrects prediction errors by optimizing weak learners, thereby improving accuracy. Additionally, their capability to manage missing data, feature interactions, and non-linearity contributed to their robust performance. BDT exhibited superior results, particularly for short-term and long-term predictions. This is likely due to its strong regularization techniques that prevent overfitting while maintaining high interpretability. RF and AdaBoost, although not as optimal as XGBoost and LGBM, performed consistently well across different time scales. These models are effective in reducing variance by aggregating multiple decision trees; however, their performance was slightly limited in cases with high data variability. ET and DT exhibited lower performance due to their tendency to overfit, particularly in long-term forecasts, where generalization becomes crucial. SVM and MLR, while interpretable, showed limitations in handling non-linearity and complex interactions between meteorological variables, leading to reduced accuracy compared to ensemble-based models.

**Table 10:** ML models comparison.

Model	Type	Strengths	Weaknesses	Interpretability	Missing values handling	Accuracy	Complexity
MLR	Single	Simplicity and ease of interpretation	Limited accuracy for complex data	Yes	No (assumes complete data)	Low	Low
SVM	Single	Effective for high-dimensional data	Sensitivity to kernel choice	No	No (requires data preprocessing)	High	High
ET	Ensemble	Robust to overfitting	Less interpretability	No	Yes (can handle missing data)	Medium	Medium
RF	Ensemble	High predictive accuracy and robustness	Reduced interpretability	No	Yes (can handle missing data)	High	Medium
DT	Ensemble	Easy to understand and visualize	Prone to overfitting	Yes	No (assumes complete data)	Medium	Low
BDT	Ensemble	Strong ensemble model with excellent accuracy	Computationally intensive	No	Yes (can handle missing data)	High	High
GB	Ensemble	Excellent predictive performance	Sensitivity to hyperparameters	No	Yes (can handle missing data)	High	High
LGBM	Ensemble	Fast and highly accurate	Less interpretability	No	Yes (can handle missing data)	High	High
XGBoost	Ensemble	Excellent accuracy and optimization	Complexity in fine-tuning	No	Yes (can handle missing data)	High	High
AdaBoost	Ensemble	Robustness to overfitting	Sensitive to noisy data	No	Yes (can handle missing data)	High	Low

### Feature importance analysis

**Fig. 8:**
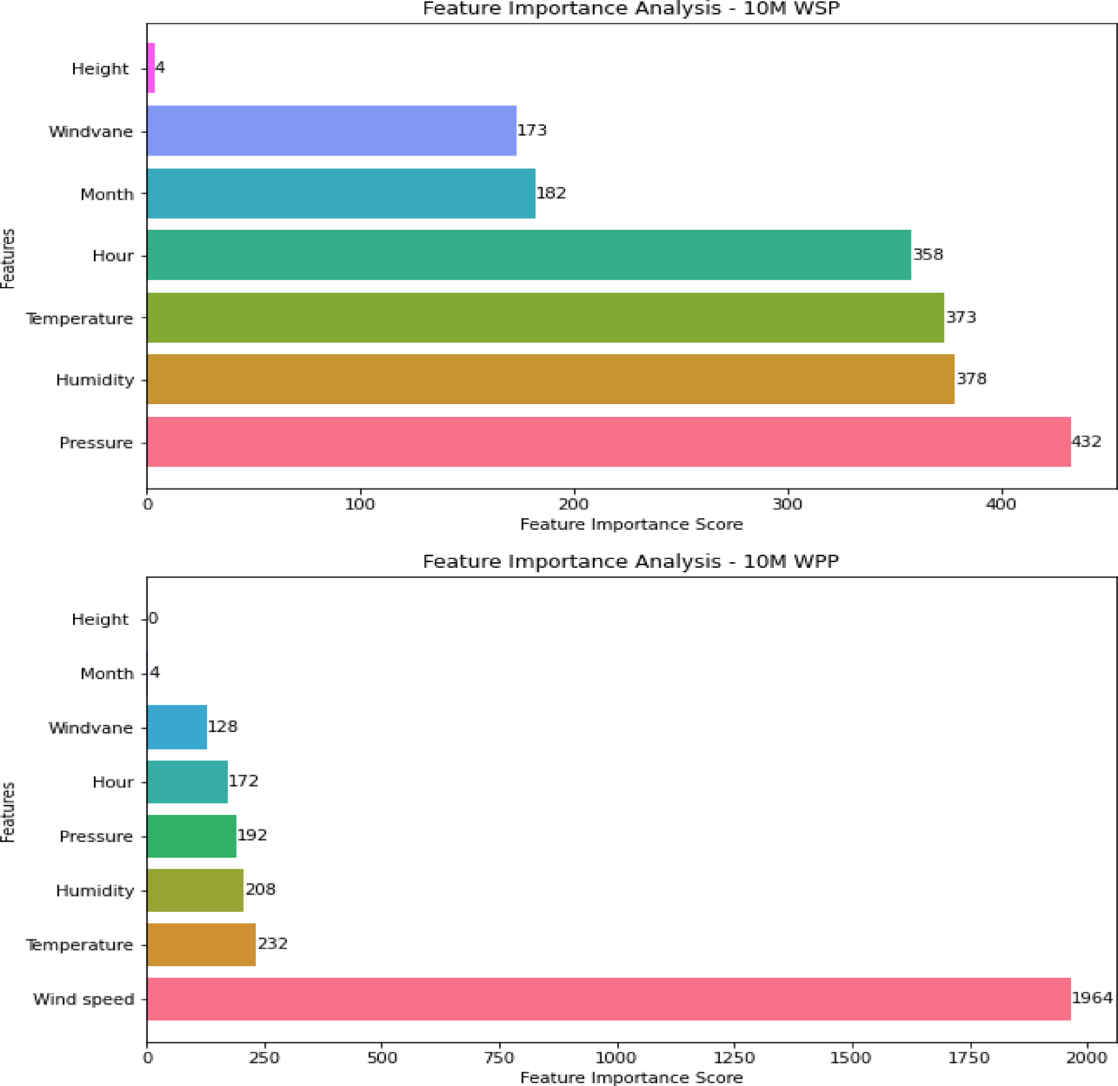
Feature importance analysis for VSTWSP and VSTWPP.

A feature importance analysis is conducted to determine how each feature (i.e., input factor) affects the model’s output (i.e., WSP and WPP). This analysis examines the effect of eliminating each factor on the predicted output to investigate its relative importance. The LGBM model-based feature significance analysis is shown in Fig. 8. According to the plot, on a 10-min time frame, air pressure has the biggest effect on VSTWSP, followed by humidity. The importance scores for humidity, temperature, and hour are nearly equal. Conversely, the height predictor has little effect on VSTWSP. The wind speed predictor has the biggest effect when taking VSTWPP into account, and this outcome upholds the high correlation between wind power and speed. Furthermore, the WPP is moderately impacted by other factors. The influence of height and month predictors on power generation is rather small.

**Fig. 9:**
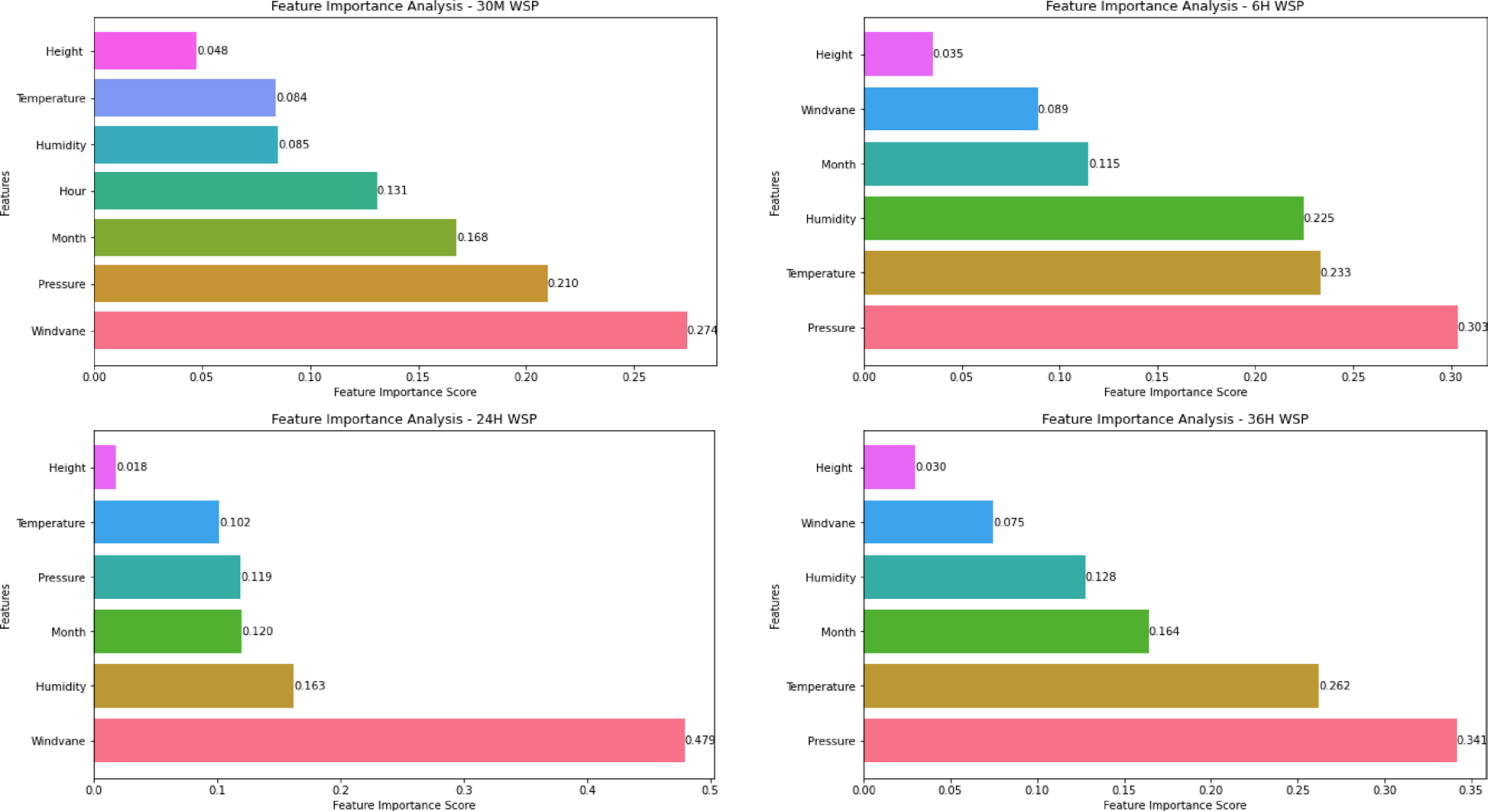
Feature importance analysis for WSP models at different time scales.

**Fig. 10:**
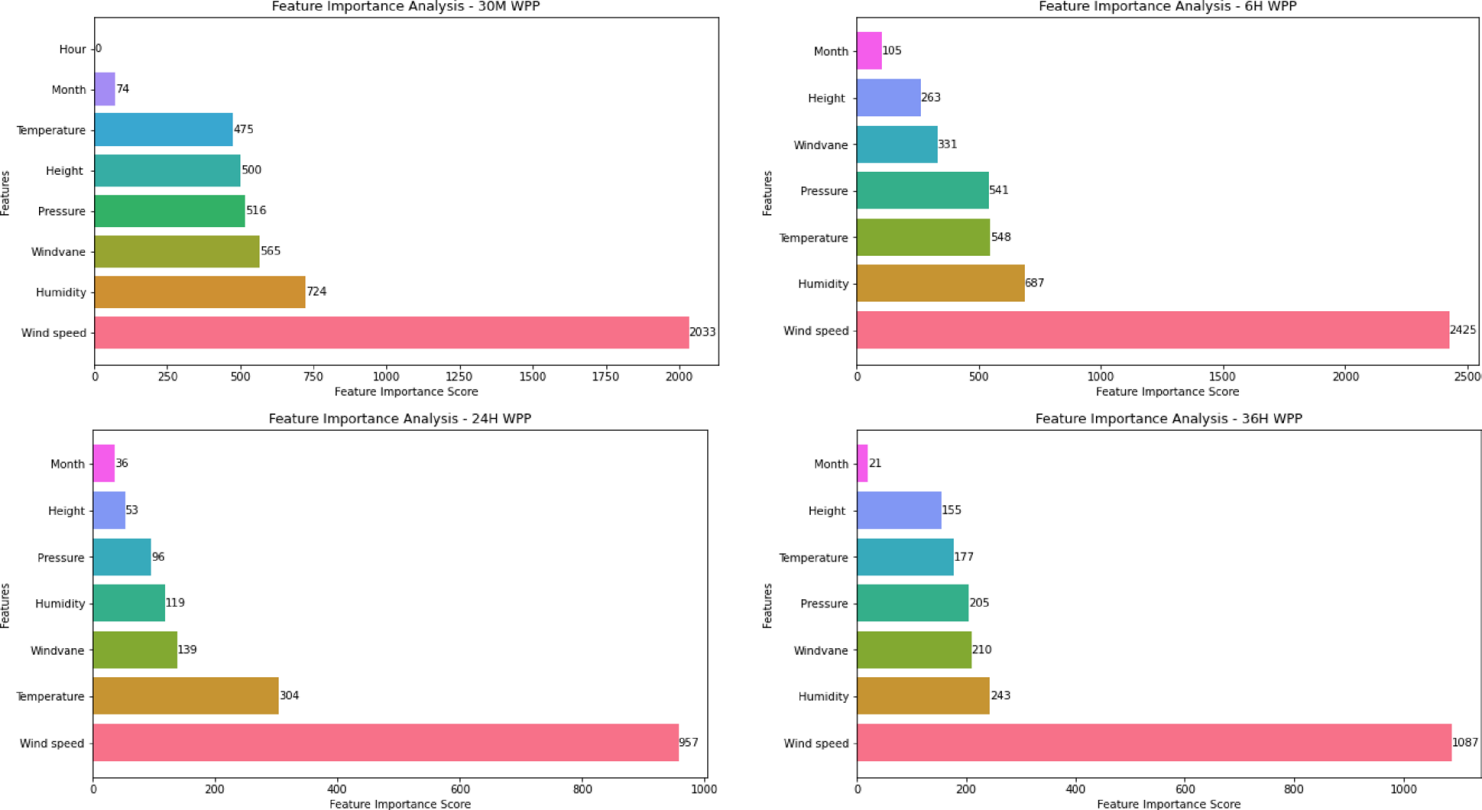
Feature importance analysis for WPP models at different time scales.

Similarly, the feature importance analysis is conducted for WSP and WPP using the optimal model at different time scales (30 M, 6 H, 24 H, and 36 H) as shown in Figs. 9 and 10. The analysis in Fig. 9 indicates that the wind-vane, air pressure, and month have the highest impact on predicting wind speed 30 min ahead. Contrarily, height and temperature predictors have a relatively low impact. Pressure, temperature, and humidity are the main predictors, according to an analysis of their effects six hours before WSP. In the meanwhile, the main determinants of 24-h WSP are month, humidity, and windvane. According to the final graphic, the primary predictor for the 36 h before WSP is air pressure. Furthermore, while height has a negligible effect, temperature, month, and humidity all have a big influence. The feature importance analysis reveals that the wind speed predictor has the most influence on various scales ahead, as illustrated in Fig. 10.

### Key findings and contributions

**Fig. 11:**
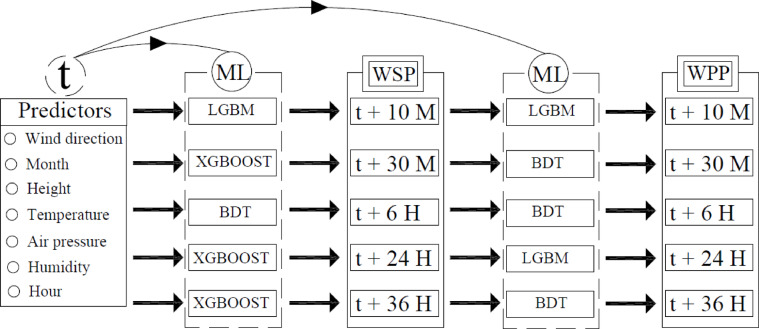
Optimal integrated models for WSP and WPP for every time scale.

1. To compare the outcomes and choose the best algorithms for WSP and WPP, a variety of evaluation methods are used. The best models for WSP and WPP at all time scales are shown in Fig. 11.
2. The study assesses how well ten machine learning models predict wind power and speed over a range of time periods. The most accurate ones are LGBM, XGBOOST, and BDT.
3. Depending on the time scale, ensemble learning models and tree-based models perform better than individual machine learning algorithms. As a result, ensemble learning algorithms may become popular in prediction applications in the future.
4. A new case study is addressed in Gabal El-Zayt wind farm (200 MW), Red Sea governorate, Egypt. The biggest publicly available dataset on wind speed and power over various time ranges is presented in this paper.
5. Feature importance analysis is conducted to evaluate the impact of the ML model’s predictors.

Therefore, this paper uniquely contributes to wind energy forecasting by addressing critical gaps in existing research, particularly the need for optimized ML models that balance accuracy and computational efficiency across multiple time horizons. While previous studies have explored ML models for WSP and WPP, they often overlook the comparative performance of tree-based ensemble methods such as LGBM, XGBoost, and BDT, especially when optimized using Bayesian techniques. By systematically evaluating ten ML models and leveraging Bayesian optimization to fine-tune hyperparameters, this research enhances both precision and computational efficiency. Additionally, the study introduces a large, publicly available dataset from the Gabal El-Zayt wind farm, providing valuable insights into wind energy forecasting in a real-world setting. The inclusion of feature importance analysis further strengthens the findings by identifying key predictors influencing wind behavior. Through these contributions, this research advances the field by developing a robust, integrated forecasting system that improves wind farm operations, supports grid stability, and demonstrates the growing importance of ensemble learning models for renewable energy applications.

## Conclusion

To maximize wind energy generation and guarantee grid stability, precise wind speed and power forecasting are crucial. This study used historical turbine data and actual meteorological data from the Gabal Al-Zayt wind farm in Egypt to forecast wind power and speed over a range of time horizons (10, 30, 6, 24, and 36 h) using Bayesian-optimized machine learning models. The outcomes showed how well ensemble-based machine learning algorithms work to increase predicting accuracy.

Specifically, the LGBM algorithm provided the best performance for very short-term wind speed forecasting with a 10-min lead time, achieving MAPE = 12.274%, MSE = 0.953, EV = 0.888, R = 0.943, and CCC = 0.939. For very short-term wind power forecasting, the same model achieved MAPE = 186.71%, MSE = 9444.576, EV = 0.97, R = 0.985, and CCC = 0.985. Over a longer horizon, the XGBoost ensemble model demonstrated high accuracy in predicting wind speed 36 h in advance (MAPE = 4.943%, MSE = 0.137, EV = 0.985, R = 0.992, CCC = 0.992), while the BDT algorithm achieved superior results for 36-h wind power prediction (MAPE = 0.888%, MSE = 1.529, EV = 1.000, R = 1.000, CCC = 1.000). Additionally, feature importance analysis revealed that wind vane direction and air pressure were the most influential predictors.

By integrating these models into wind power systems, operators can enhance power regulation strategies, improve electricity market participation, and contribute to grid stability. Despite these contributions, a key limitation of this research is the generalizability of the models beyond the Gabal Al-Zayt wind farm. To ensure broader applicability, further validation is required across different geographic regions and datasets spanning diverse climatic conditions and historical timeframes. Addressing this limitation will provide deeper insights into the adaptability and robustness of the proposed ML models in real-world wind energy forecasting applications.

To build upon the findings of this study, we recommend the following research directions:

- Further exploration of ensemble methods by modifying base learners and evaluating their performance at finer (seconds-level) and coarser (hourly or monthly) time scales.
- Given the stochastic nature of wind energy, integrating fuzzy logic and probabilistic models into ML frameworks can improve reliability and robustness.
- The hybridization of ML models with metaheuristic algorithms, such as genetic algorithms or particle swarm optimization, can further enhance predictive accuracy and computational efficiency.

By addressing these challenges, future research can continue advancing ML-driven wind energy forecasting, improving the sustainability and reliability of wind power integration into modern energy systems.

## Supplementary Information

Supplementary Information 1.

## Data Availability

The data is available in the following repository: https://github.com/HaythamElmousalami/Wind-Energy-and-Machine-learning.

## Abbreviations

AdaBoost: Adaptive boosting
ANN: Artificial neural network
BDT: Bagged decision tree
CCC: Concordance correlation coefficient
EV: Explained variance
XGBoost: Extreme gradient boosting
ET: Extremely randomized trees
GW: Gigawatts
MSE: Mean square error
MB: Megabyte
MW: Megawatts
MLP: Multilayer perceptron
MLR: Multiple linear regression
NWP: Numerical weather prediction
R: Pearson’s correlation
RBF: Radial basis function
SVM: Support vector machine
VSTWPP: Very short-term wind power prediction (KW/turbine)
VSTWSP: Very short-term wind speed prediction (m/s)
