# Supplementary material for: Wind speed and power forecasting using Bayesian optimized machine learning models in Gabal Al-Zayt, Egypt
Source: Sci Rep. 2025 Aug 5;15:28500. doi: 10.1038/s41598-025-13140-x (PMC12325623; doi:10.1038/s41598-025-13140-x)
Supplement: Supplementary file 1 — Supplementary Information 1. [file 41598_2025_13140_MOESM1_ESM.docx]

**Appendix A.** Bayesian hyperparameter optimization for the ML models

**Table A1** Descriptions of the optimized hyperparameters

| **ID** | **Model** | **Number of parameters** | **Parameters** |
| --- | --- | --- | --- |
| 1 | MLR | 3 | 1. **fit_intercept:** Boolean parameter to determine whether to calculate the intercept for the model. 2. **copy_X:** Boolean parameter to indicate whether to make a copy of the input data before fitting the model. 3. **positive:** Boolean parameter enforcing non-negative coefficients in certain models. |
| 2 | SVM | 4 | 1. **kernel:** Type of kernel used in the SVR model. 2. **gamma:** Kernel coefficient for 'rbf'. 3. **C:** Regularization parameter (penalty) in the SVR model, controlling the trade-off between maximizing the margin and minimizing the error. 4. **epsilon:** Epsilon-tube around the regression function, specifying the margin within which no penalty is associated with errors in the SVR model. |
| 3 | ET | 8 | 1. **n_estimators:** Number of trees. 2. **criterion:** Function applied to measure the quality of a split. 3. **max_depth:** List of integers or ‘None’, representing the maximum depth of the individual decision trees. If ‘None’, nodes are expanded until all leaves are pure or until the minimum samples required for a split are reached. 4. **min_samples_split:** Minimum number of samples required to split an internal node. 5. **min_samples_leaf:** Minimum number of samples required to be at a leaf node. 6. **min_weight_fraction_leaf:** Minimum weighted fraction of the total weight of the samples required to be at a leaf node. 7. **max_features:** Number of features to consider when looking for the best split. 8. **max_leaf_nodes:** List of integers or ‘None’ representing the maximum number of leaf nodes. If ‘None’, the number of leaf nodes is unlimited. |
| 4 | RF | 8 | - 1. **n_estimators:** Number of trees.   2. **criterion:** Function applied to measure the quality of a split.   3. **max_depth:** List of integers or ‘None’, representing the maximum depth of the individual decision trees. If ‘None’, nodes are expanded until all leaves are pure or until the minimum samples required for a split are reached.   4. **min_samples_split:** Minimum number of samples required to split an internal node.   5. **min_samples_leaf:** Minimum number of samples required to be at a leaf node.   6. **min_weight_fraction_leaf:** Minimum weighted fraction of the total weight of the samples required to be at a leaf node.   7. **max_features:** Number of features to consider when looking for the best split.   8. **max_leaf_nodes:** List of integers or ‘None’ representing the maximum number of leaf nodes. If ‘None’, the number of leaf nodes is unlimited. |
| 5 | DT | 8 | 1. **criterion:** Function applied to measure the quality of a split. 2. **splitter:** Strategy used to choose the split at each node. 3. **max_depth:** List of integers or ‘None’, representing the maximum depth of the tree. If `None`, nodes are expanded until all leaves are pure or until the minimum samples required for a split are reached. 4. **min_samples_split:** Minimum number of samples required to split an internal node. 5. **min_samples_leaf:** Minimum number of samples required to be at a leaf node. 6. **min_weight_fraction_leaf:** Minimum weighted fraction of the total weight of the samples required to be at a leaf node. 7. **max_features:** Number of features to consider when looking for the best split. 8. **max_leaf_nodes:** List of integers or ‘None’ representing the maximum number of leaf nodes. If ‘None’, the number of leaf nodes is unlimited. |
| 6 | BDT | 3 | 1. **n_estimators:** Number of base decision trees. 2. **max_samples:** Fraction of samples to draw from the training dataset for each base estimator. 3. **max_features:** Fraction of features to consider when looking for the best split for each base estimator. |
| 7 | GB | 11 | 1. **loss:** Loss function to be optimized. 2. **learning_rate:** Step size shrinkage to prevent overfitting by scaling the contribution of each base estimator. 3. **n_estimators:** Number of boosting stages (base estimators) to be combined in the ensemble model. 4. **subsample:** Fraction of samples to be used for fitting the base estimators. 5. **criterion:** Function applied to measure the quality of a split. 6. **min_samples_split:** Minimum number of samples required to split an internal node. 7. **min_samples_leaf:** Minimum number of samples required to be at a leaf node. 8. **min_weight_fraction_leaf:** Minimum weighted fraction of the total weight of the samples required to be at a leaf node. 9. **max_depth:** List of integers or ‘None’, representing the maximum depth of the individual base estimators. If ‘None’, nodes are expanded until all leaves are pure or until the minimum samples required for a split are reached. 10. **max_features:** Number of features to consider when looking for the best split for each base estimator. 11. **max_leaf_nodes:** List of integers or `None` representing the maximum number of leaf nodes. If ‘None’, the number of leaf nodes is unlimited. |
| 8 | LGBM | 7 | 1. **boosting_type:** Type of boosting algorithm. 2. **num_leaves:** Maximum number of leaves in one tree. 3. **max_depth:** List of integers or ‘None’, representing the maximum depth of the individual trees. If ‘None’, nodes are expanded until all leaves are pure or until the minimum data required for a split is reached. 4. **learning_rate:** Shrinkage rate of the learning process, using a logarithmic scale. 5. **min_child_samples:** Minimum number of data points required to form a new node. 6. **bagging_freq:** Frequency for performing bagging. 7. **bagging_fraction:** Fraction of data points to be used for bagging. |
| 9 | XGBoost | 7 | 1. **learning_rate:** List of floats or ‘log-uniform’, representing the step size shrinkage during the boosting process. 2. **max_depth:** Maximum depth of the individual trees (base learners). 3. **reg_lambda:** L2 regularization term (lambda) used to prevent overfitting. 4. **n_estimators:** Number of boosting rounds (base learners). 5. **min_child_weight:** Minimum sum of instance weight (hessian) needed in a child node. 6. **subsample:** Fraction of samples to be used for training each base learner. 7. **colsample_bytree:** Fraction of features to be randomly selected for each tree (base learner). |
| 10 | AdaBoost | 3 | 1. **n_estimators:** Number of base estimators. 2. **learning_rate:** Step size shrinkage during the boosting process, controlling the contribution of each base estimator to the final prediction. 3. **loss:** Loss function used to update the weights of the samples. |

**Appendix B.** Visualized plots of the developed models

| 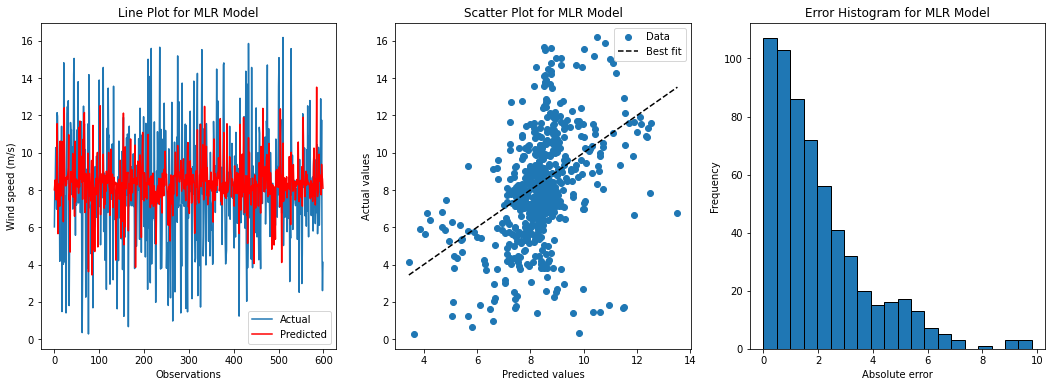 | 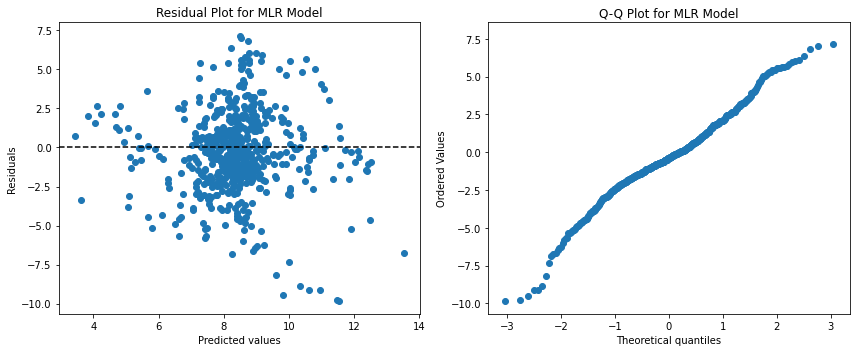 |
| --- | --- |
| 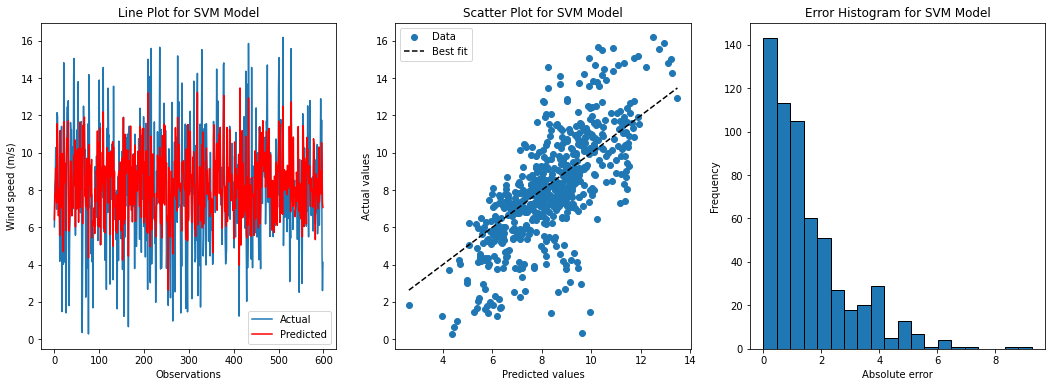 | 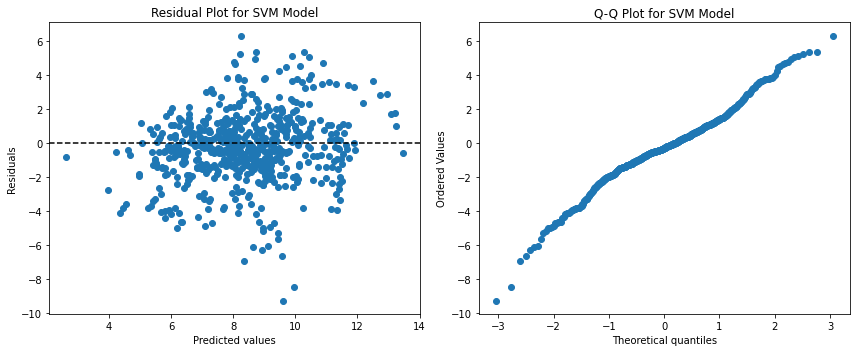 |
| 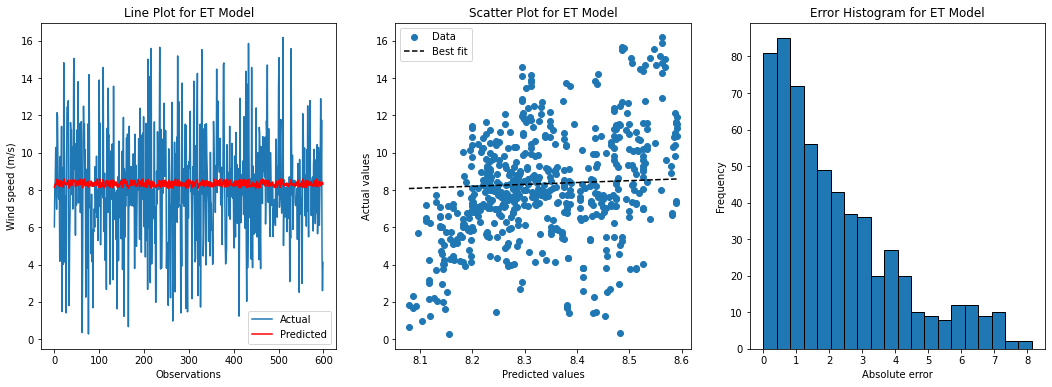 | 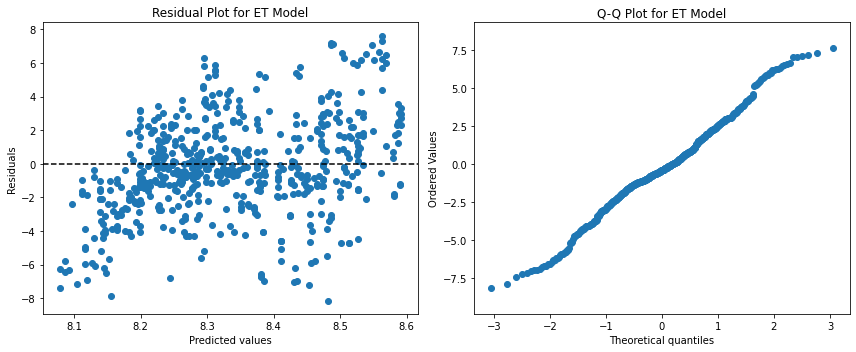 |
| 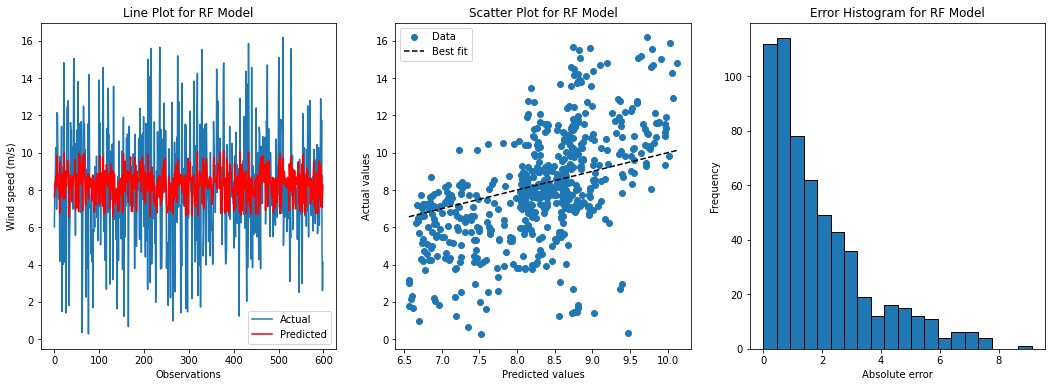 | 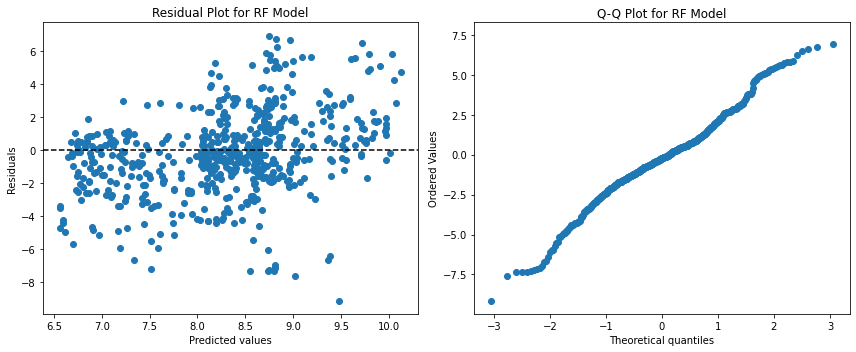 |
| 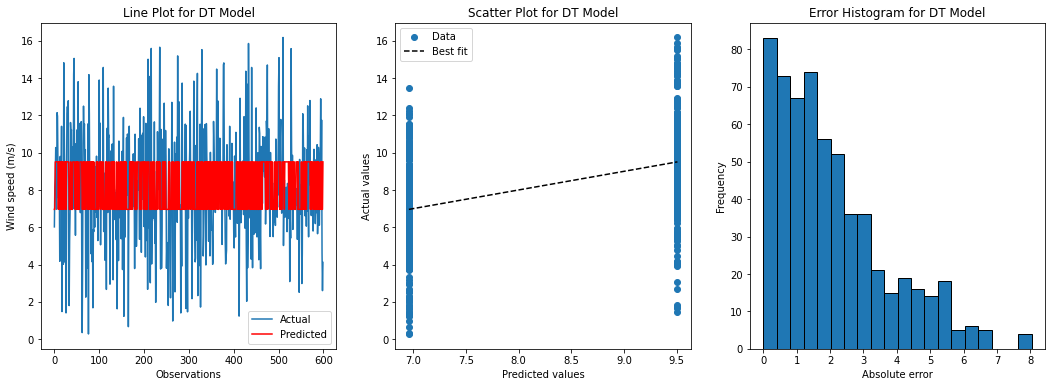 | 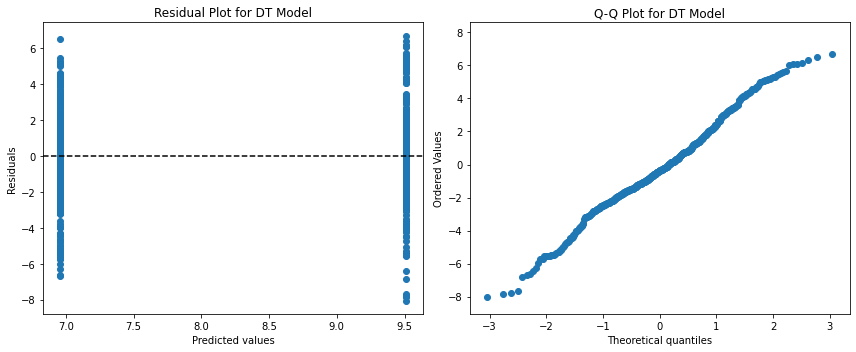 |
| 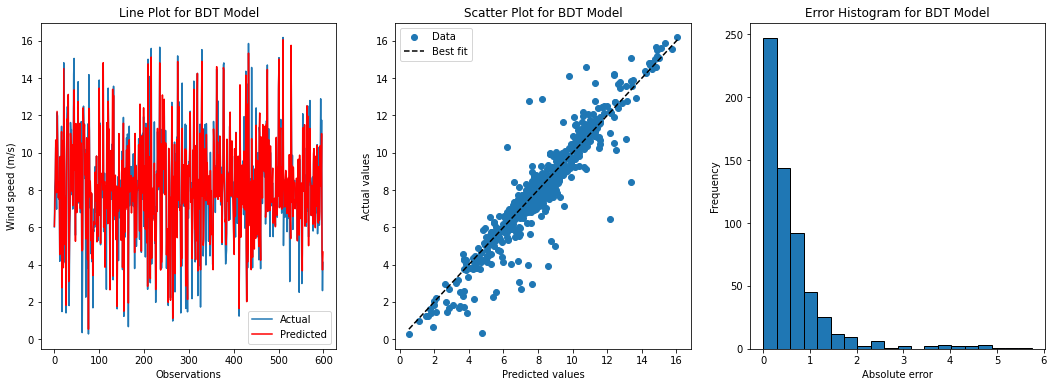 | 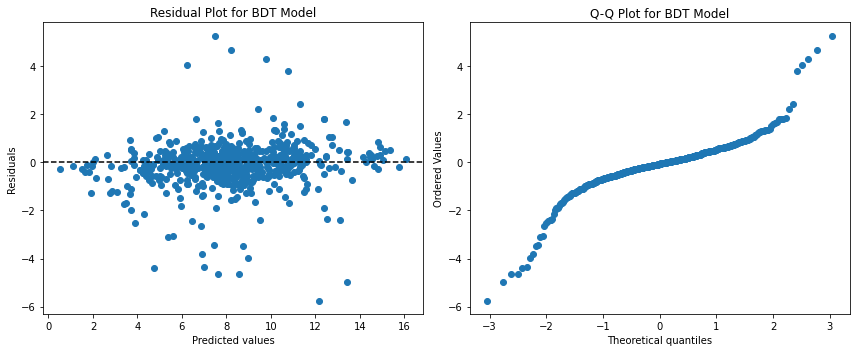 |
| 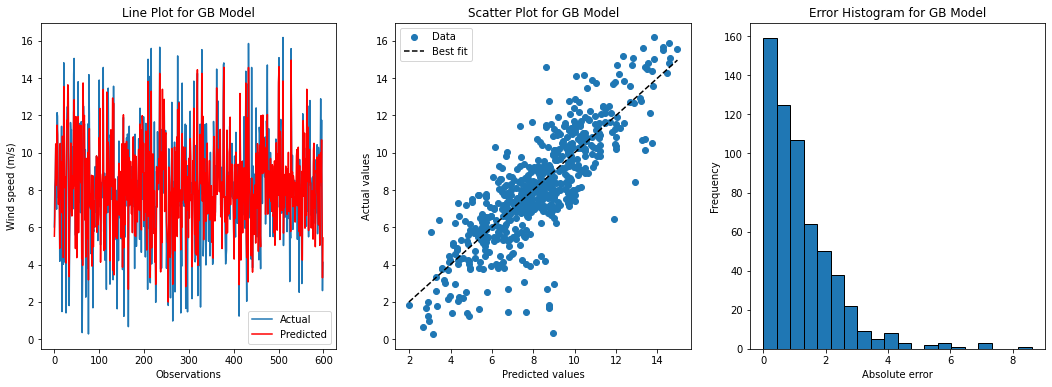 | 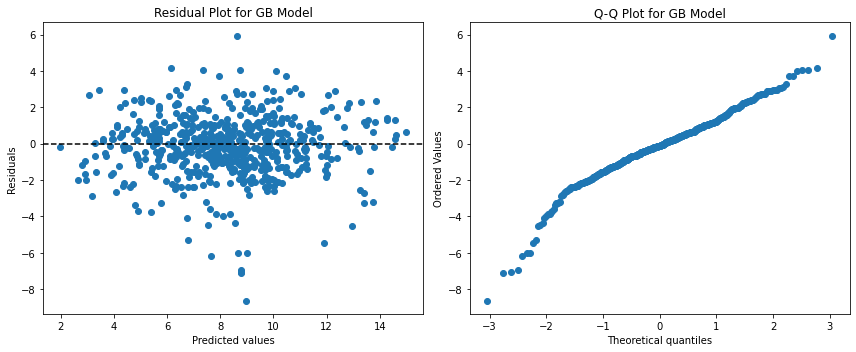 |
| 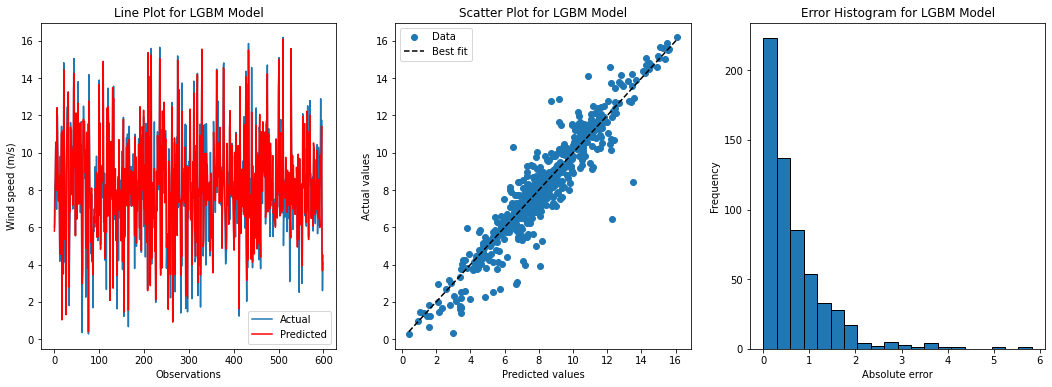 | 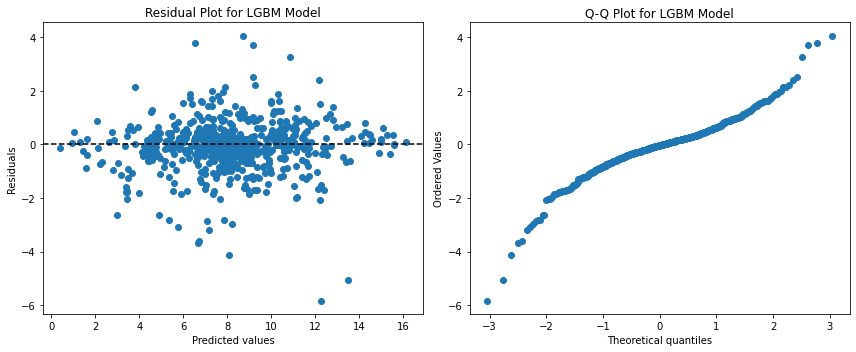 |
| 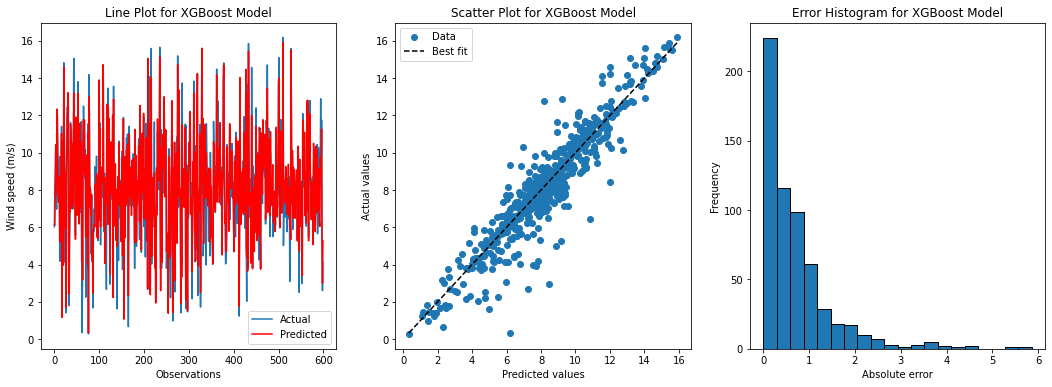 | 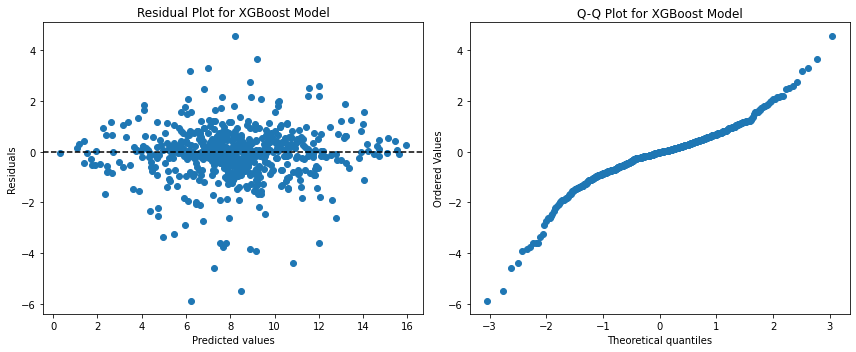 |
| 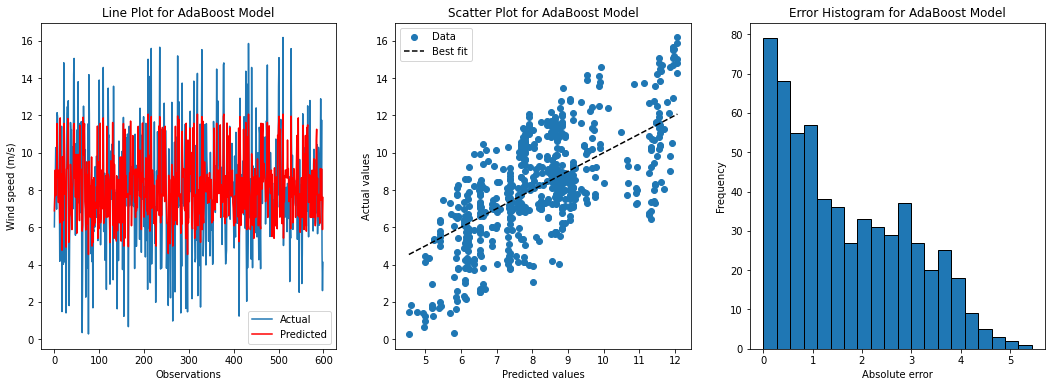 | 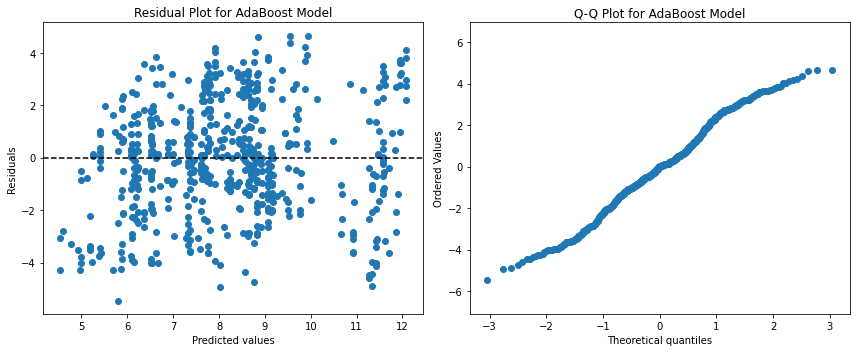 |

**Fig. B1** Visualization of the performance of developed models for predicting 10M ahead wind speed in terms of a) line plot, scatter plot, and error histogram and b) residual plot and q-q plot.

| 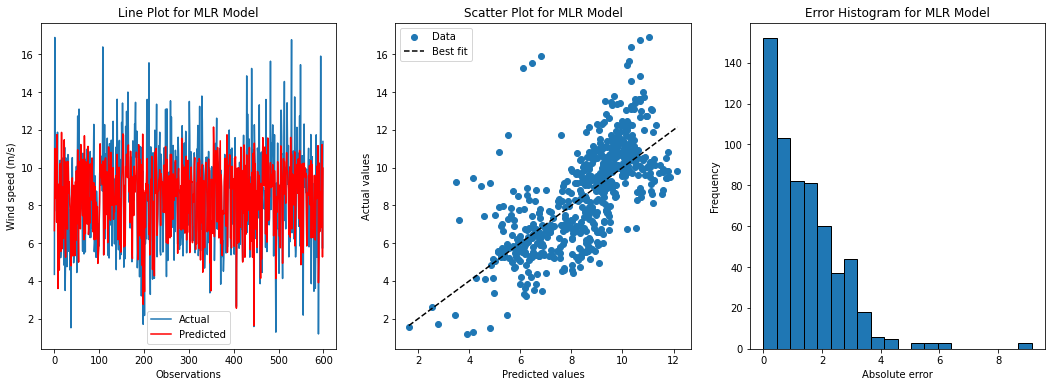 | 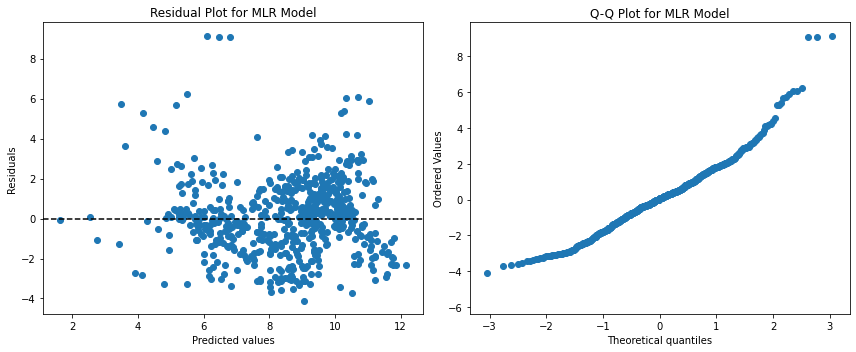 |
| --- | --- |
| 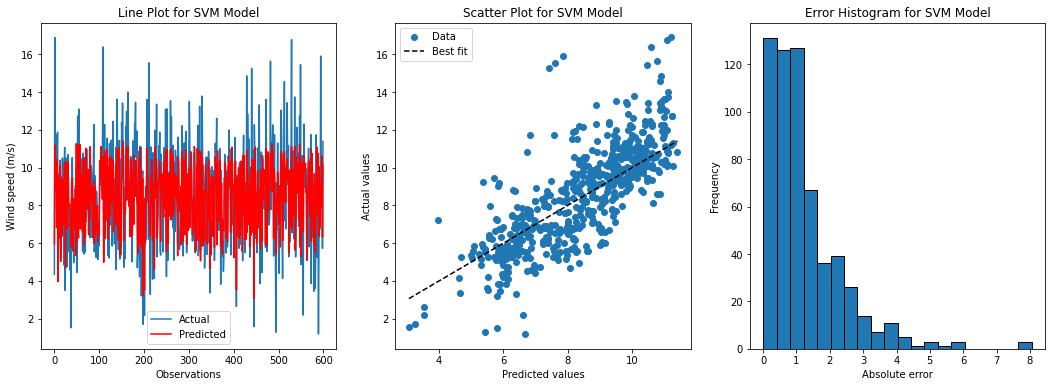 | 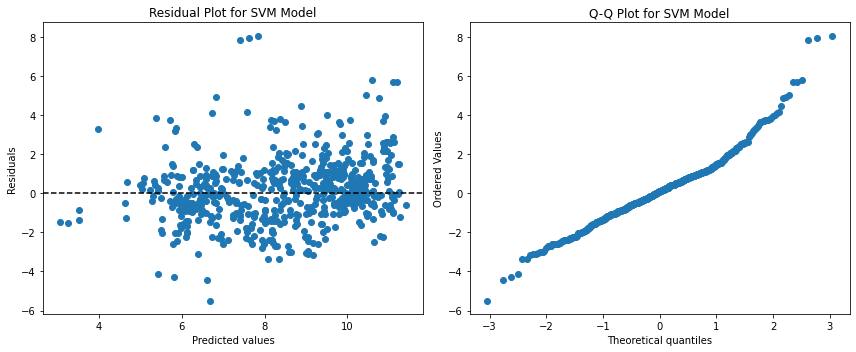 |
| 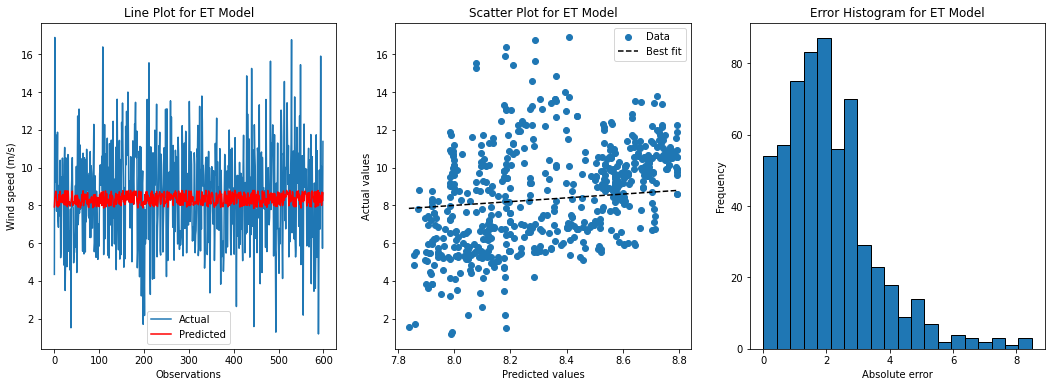 | 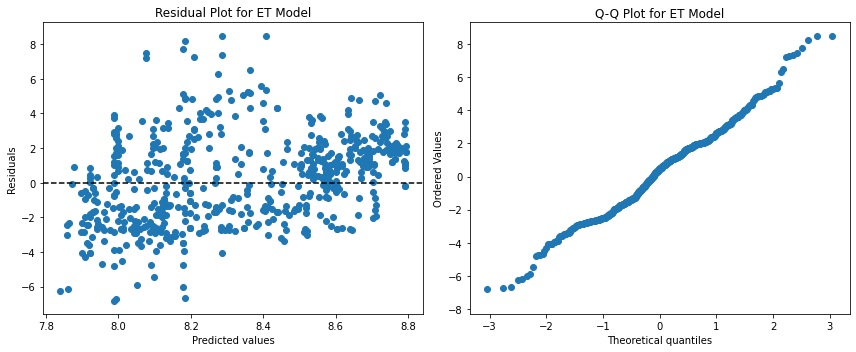 |
| 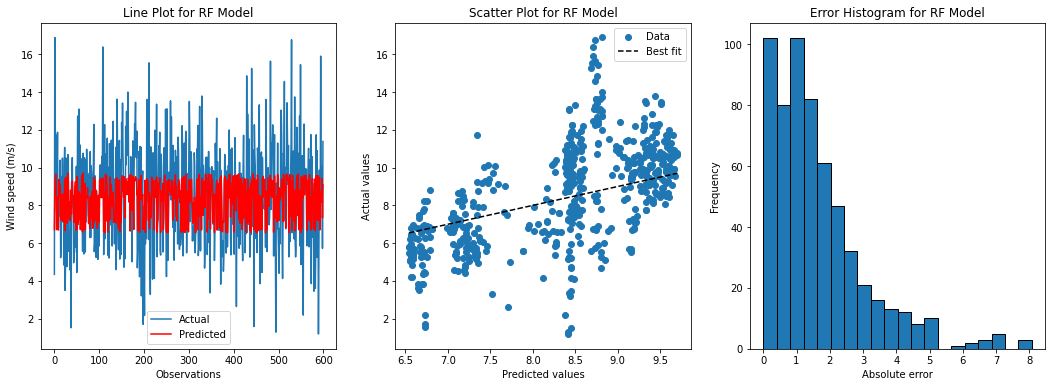 | 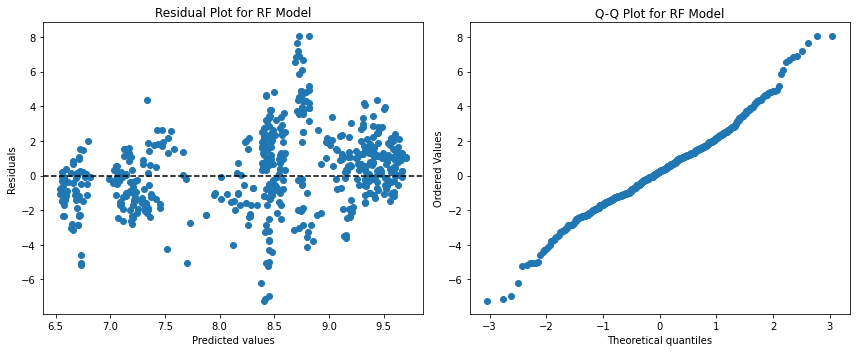 |
| 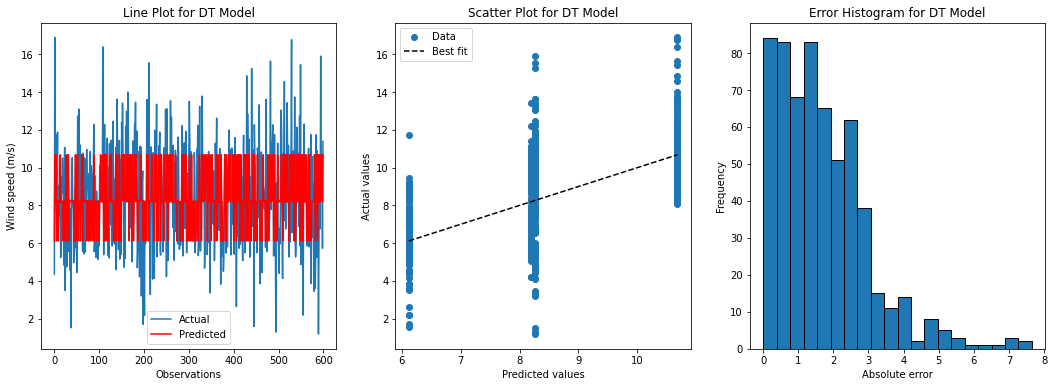 | 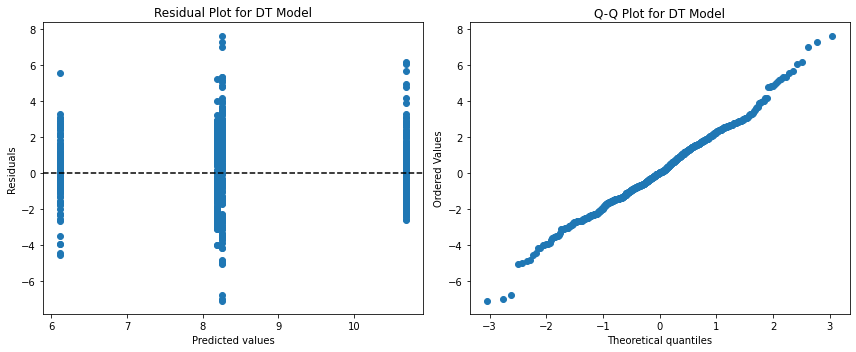 |
| 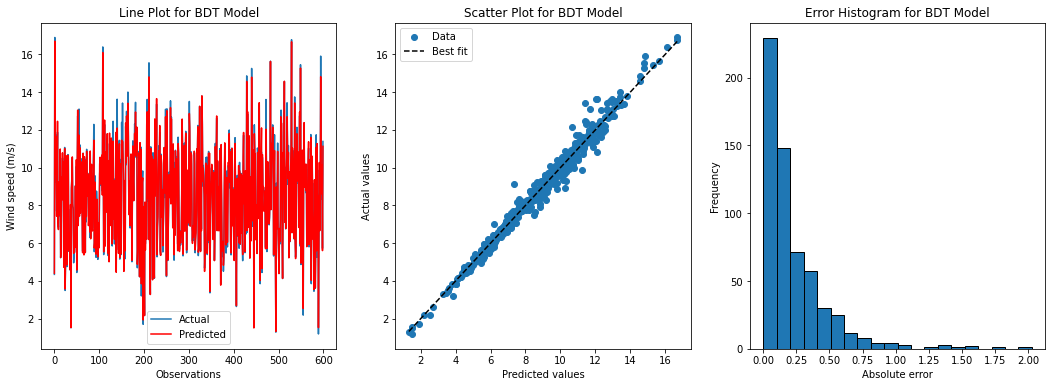 | 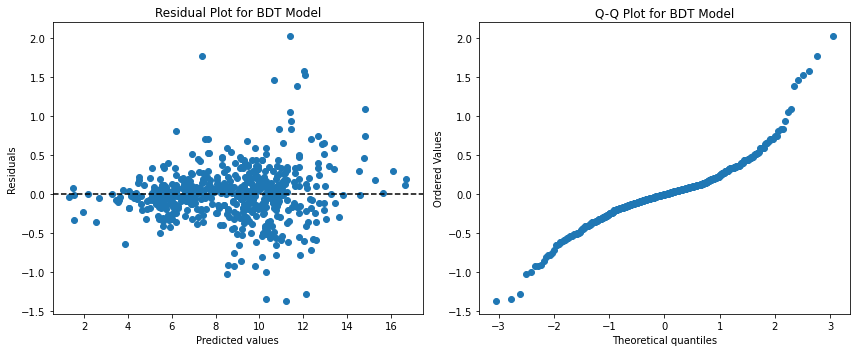 |
| 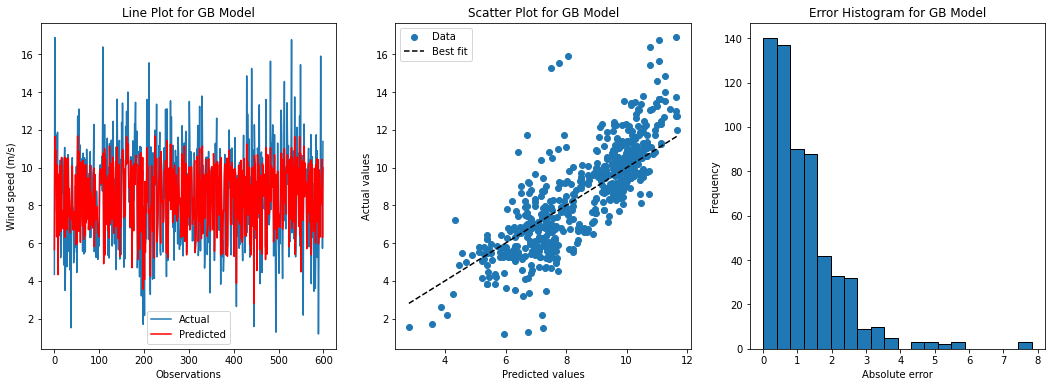 | 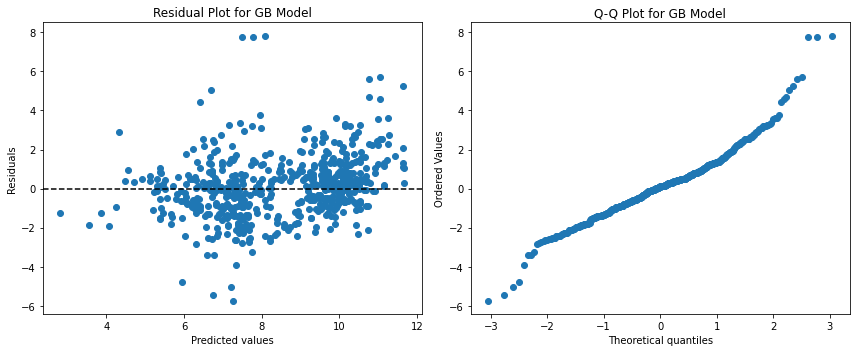 |
| 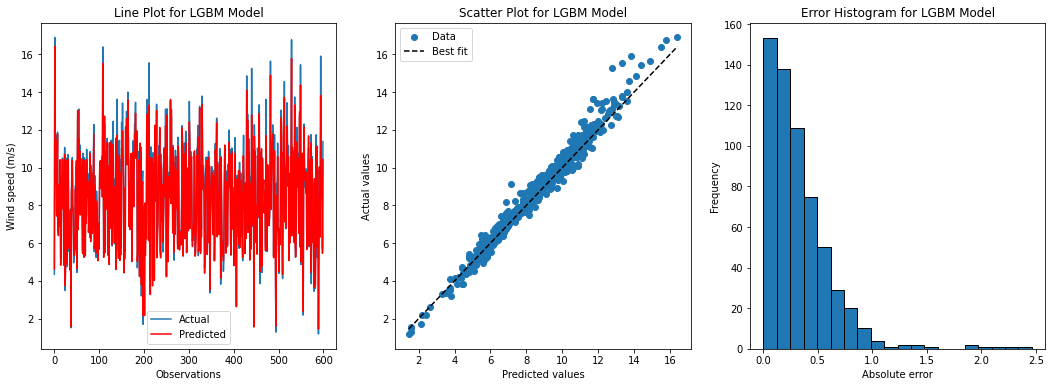 | 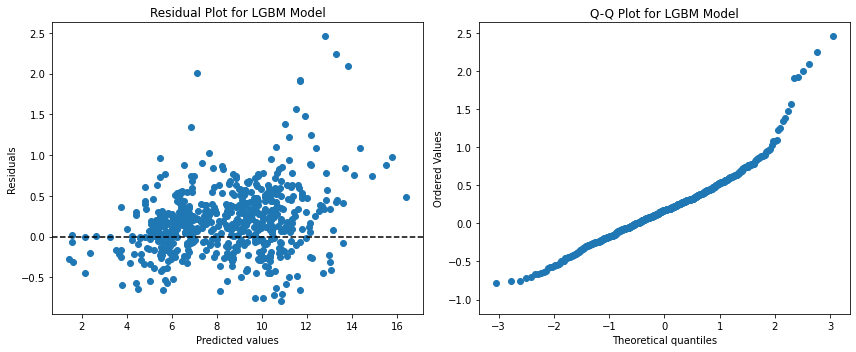 |
| 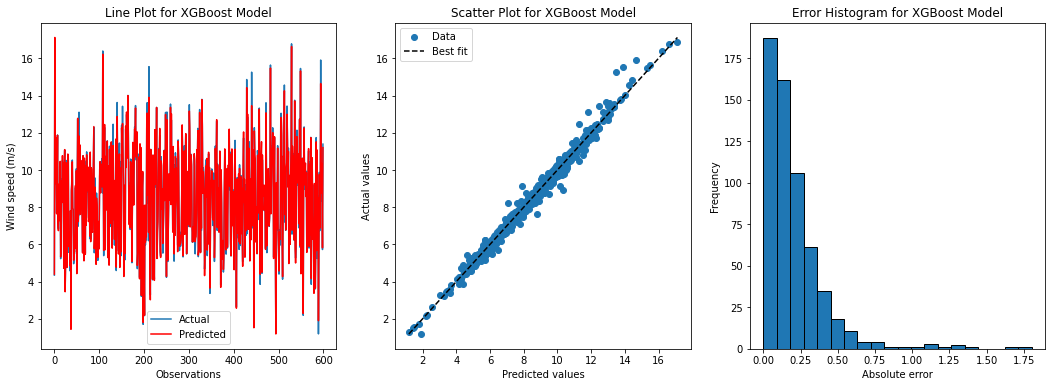 | 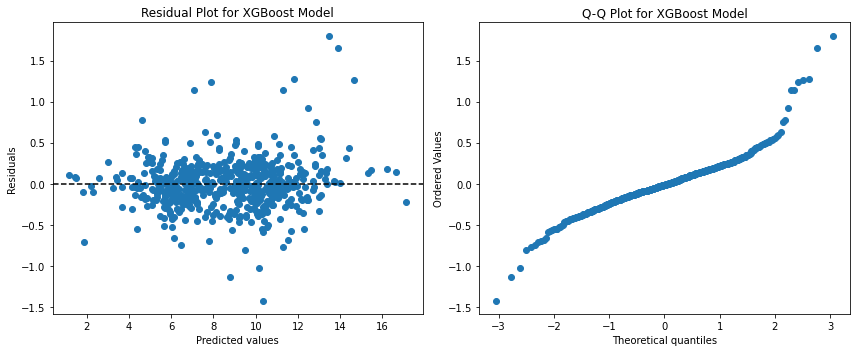 |
| 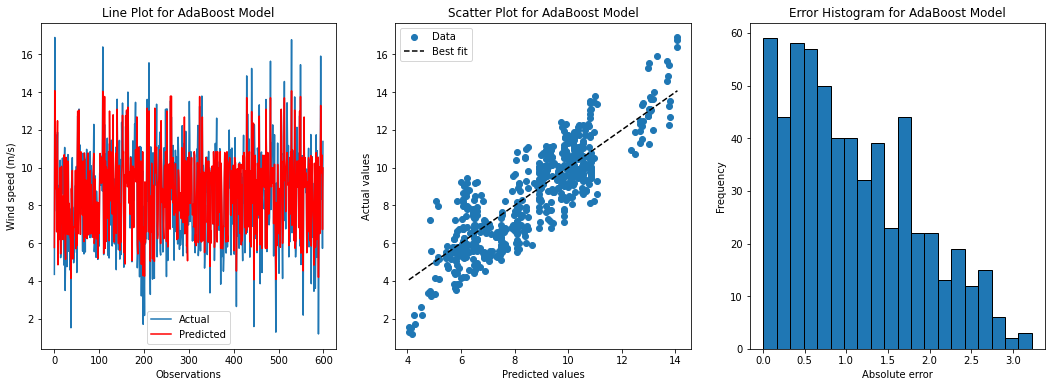 | 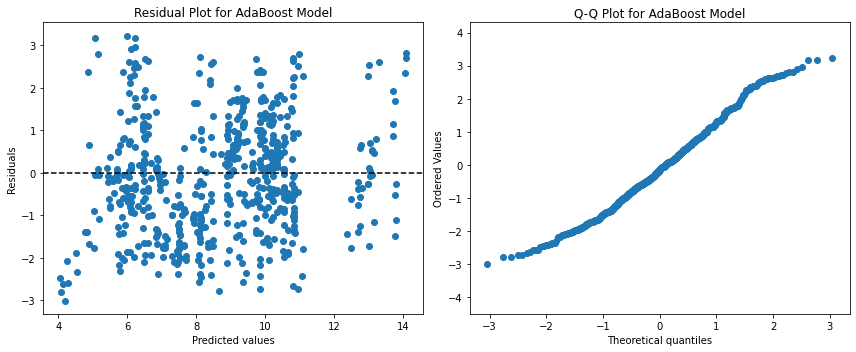 |

**Fig. B2** Visualization of the performance of developed models for predicting 30M ahead wind speed in terms of a) line plot, scatter plot, and error histogram and b) residual plot and q-q plot.

| 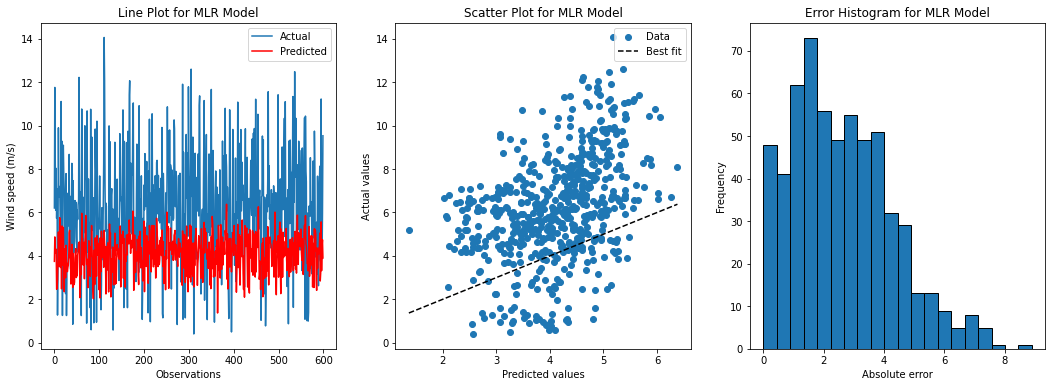 | 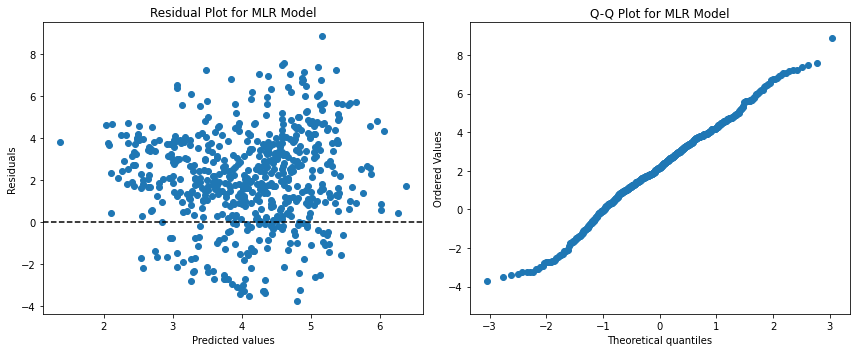 |
| --- | --- |
| 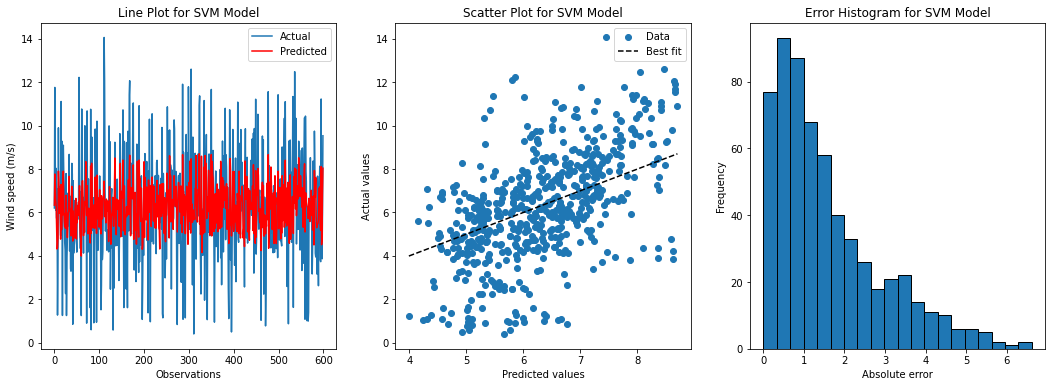 | 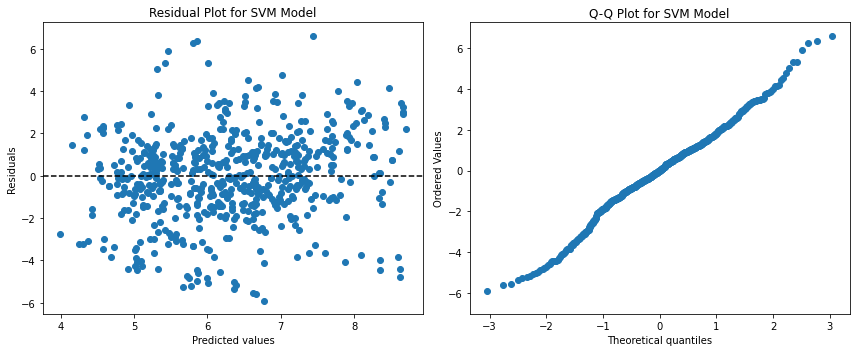 |
| 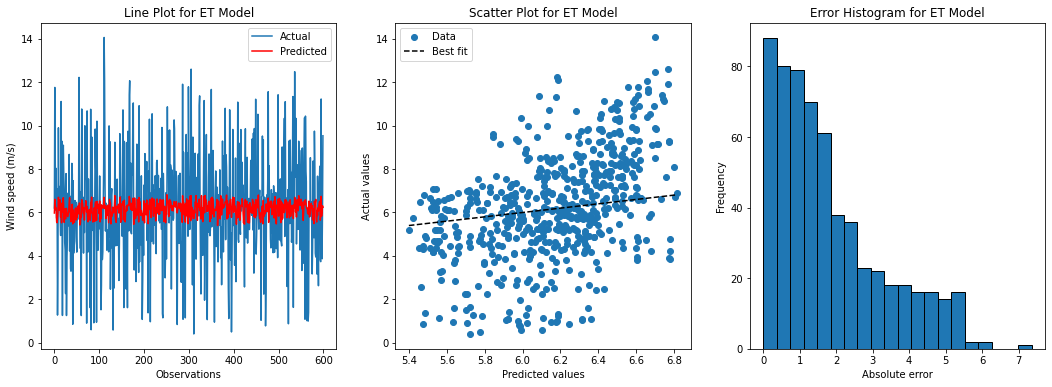 | 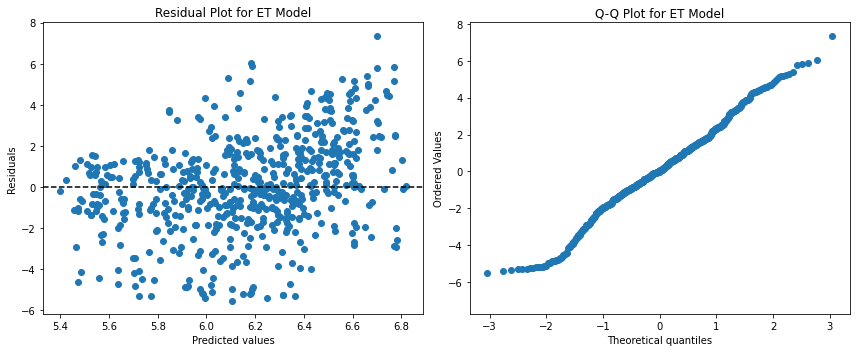 |
| 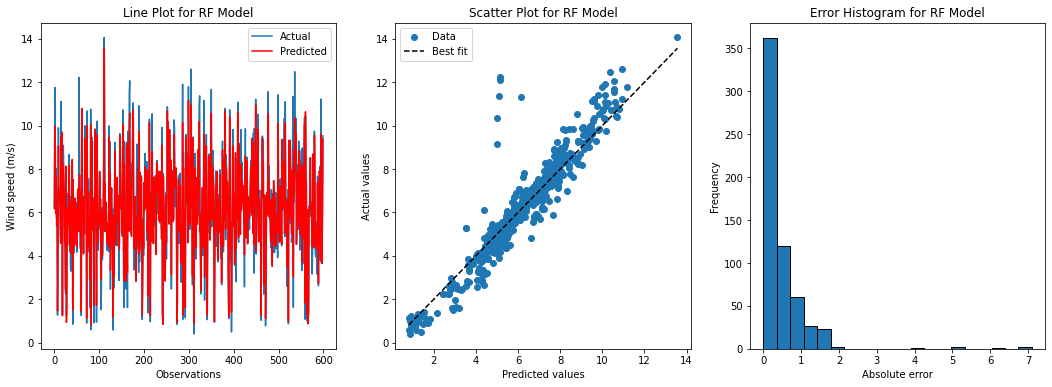 | 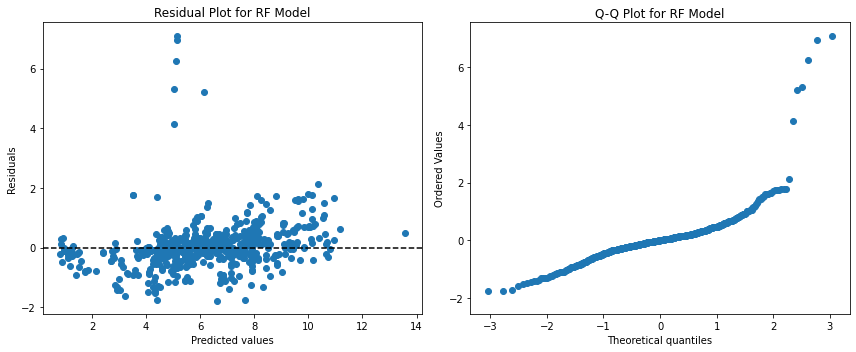 |
| 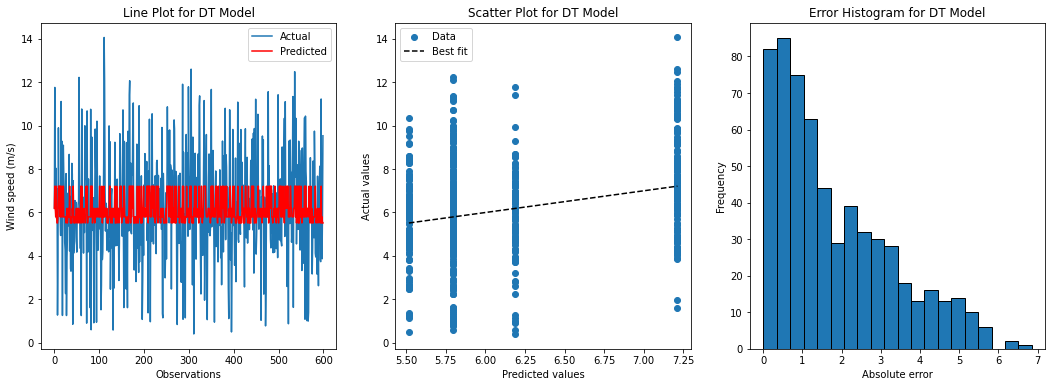 | 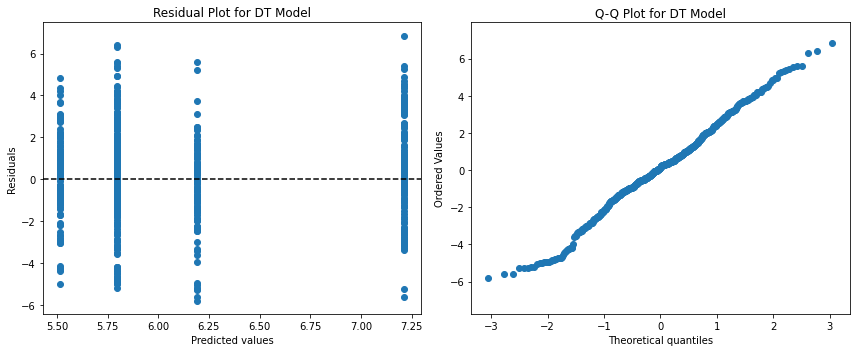 |
| 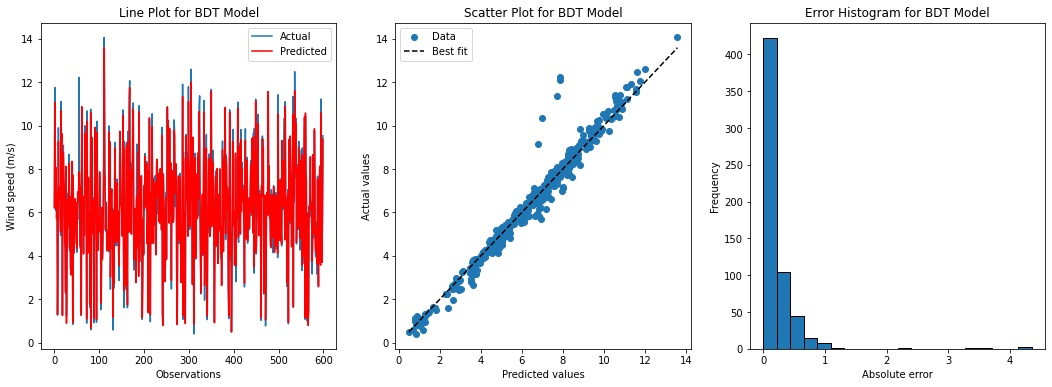 | 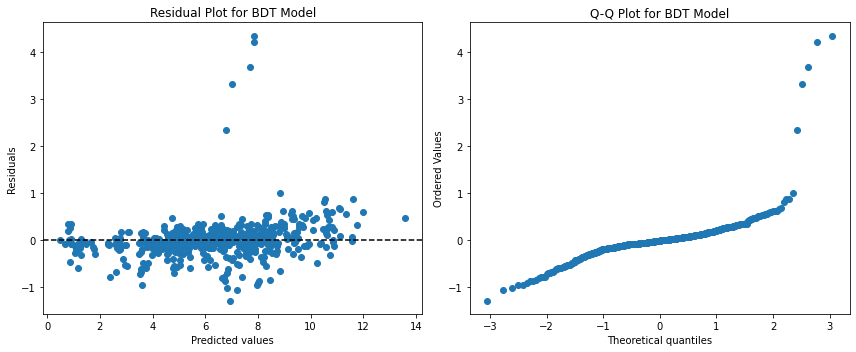 |
| 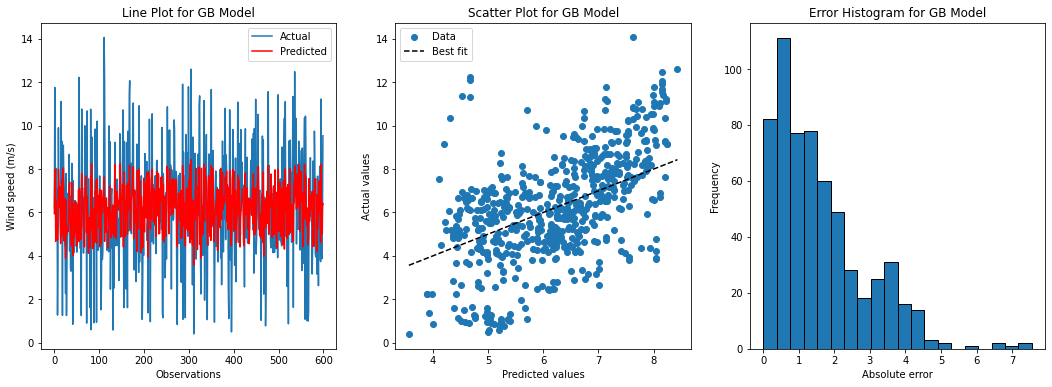 | 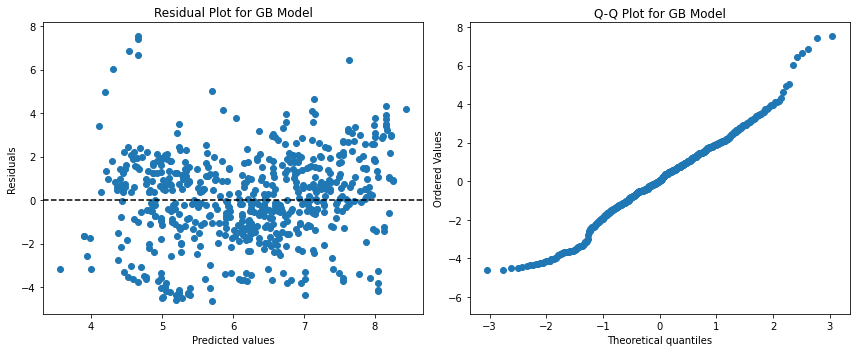 |
| 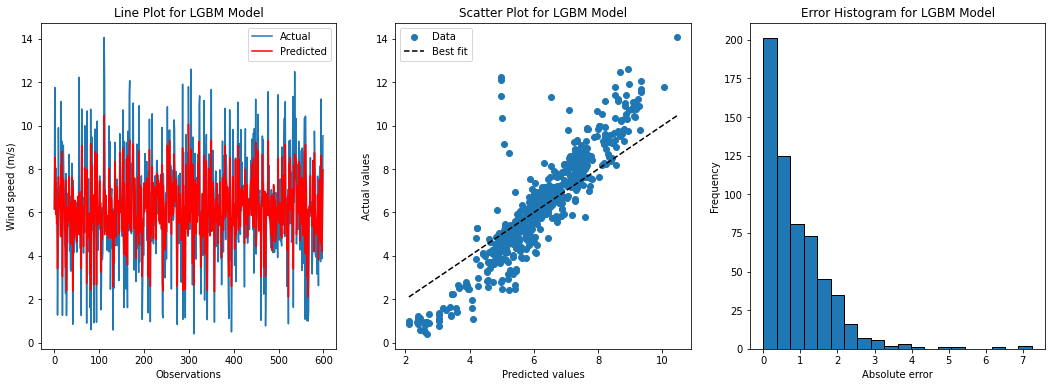 | 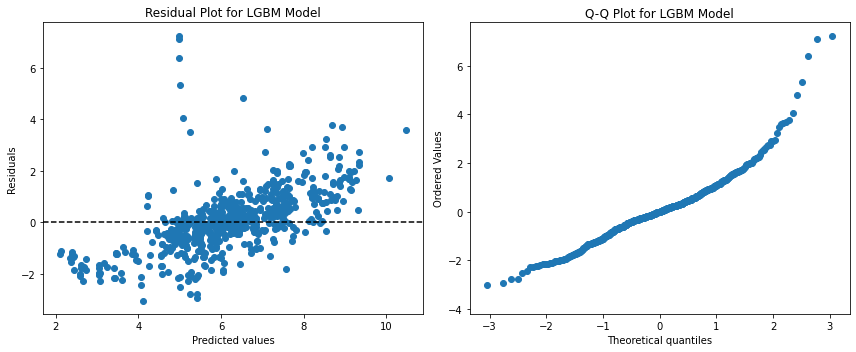 |
| 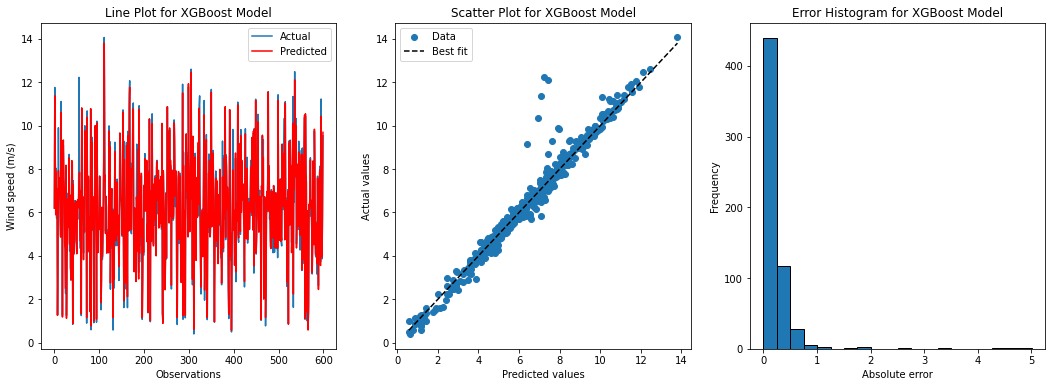 | 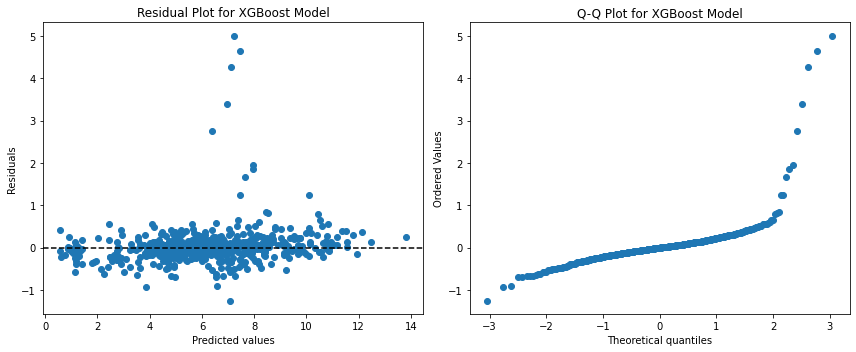 |
| 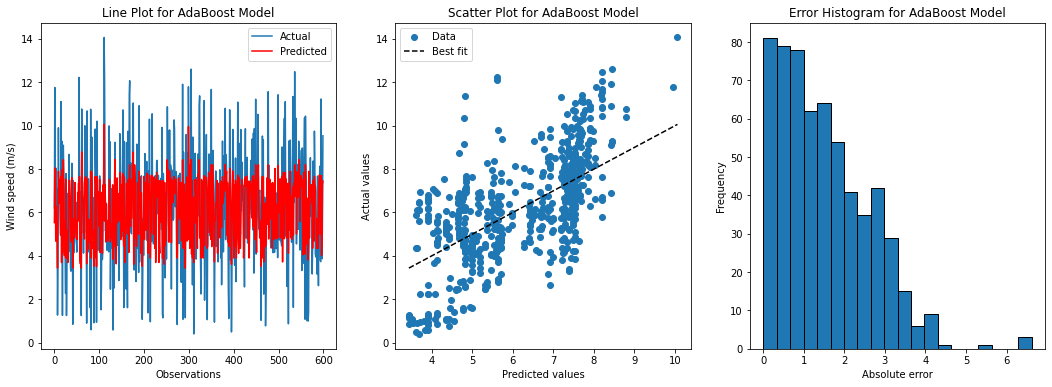 | 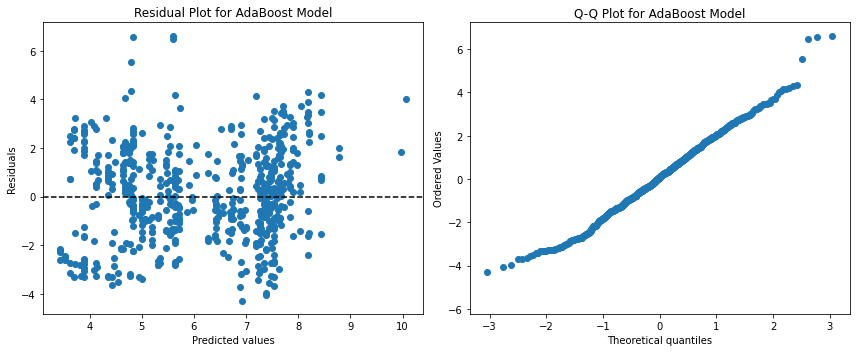 |

**Fig. B3** Visualization of the performance of developed models for predicting 6H ahead wind speed in terms of a) line plot, scatter plot, and error histogram and b) residual plot and q-q plot.

| 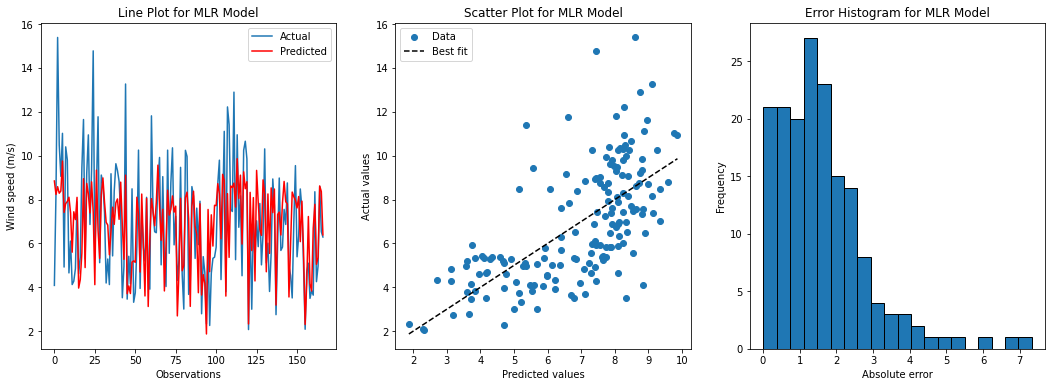 | 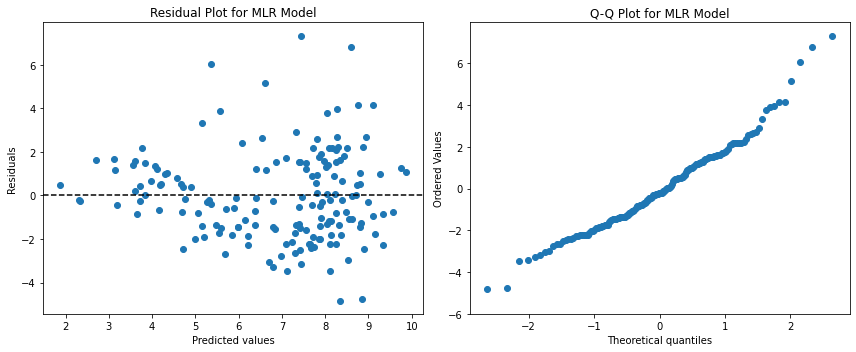 |
| --- | --- |
| 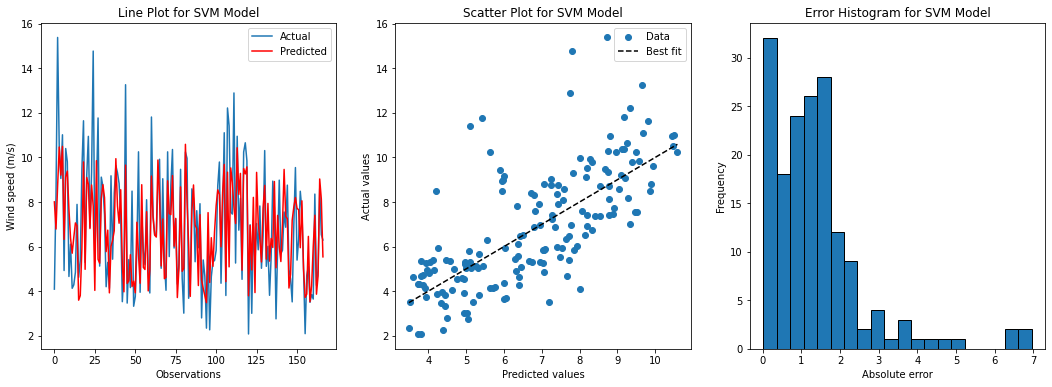 | 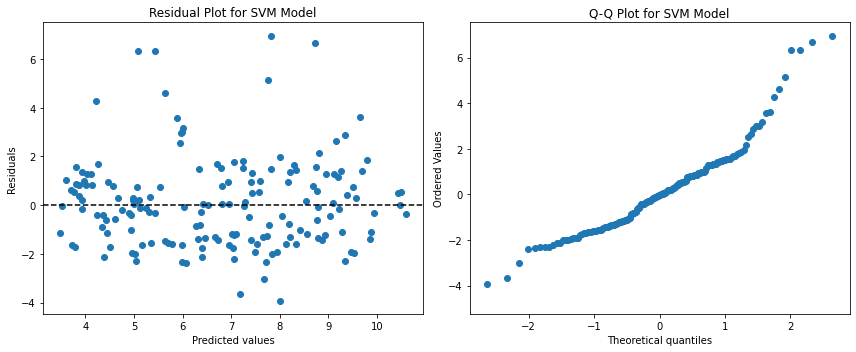 |
| 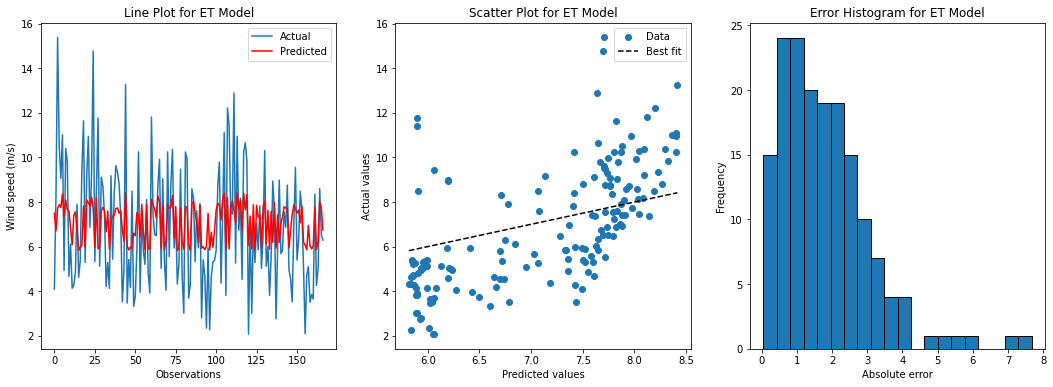 | 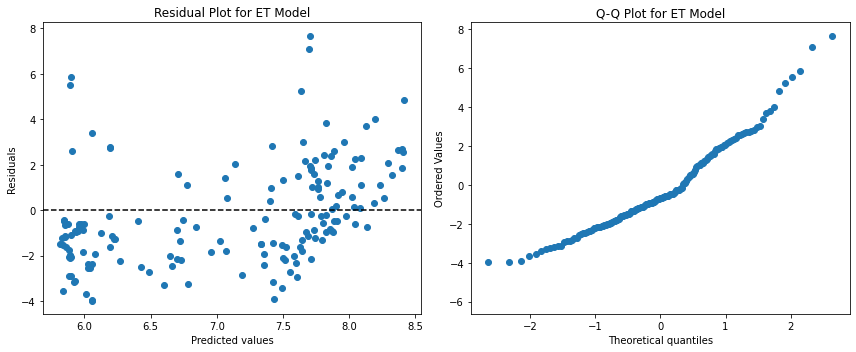 |
| 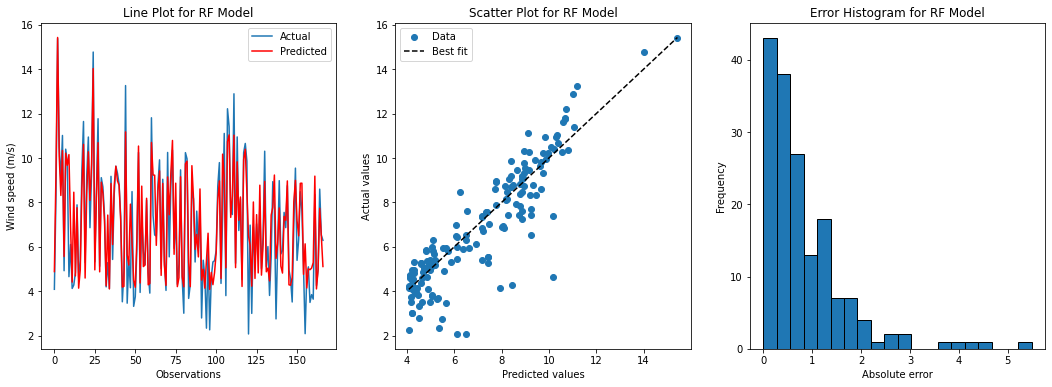 | 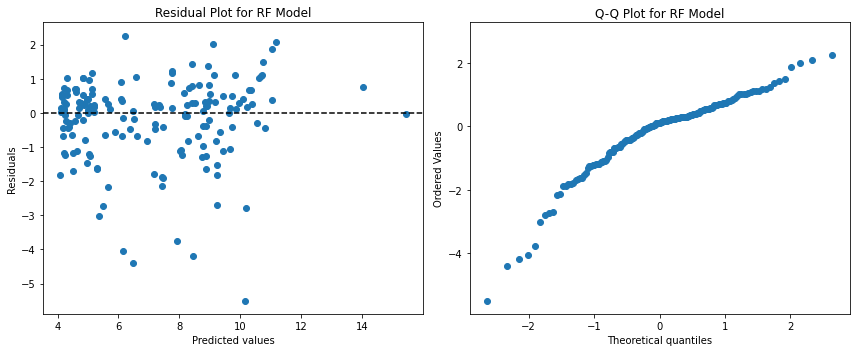 |
| 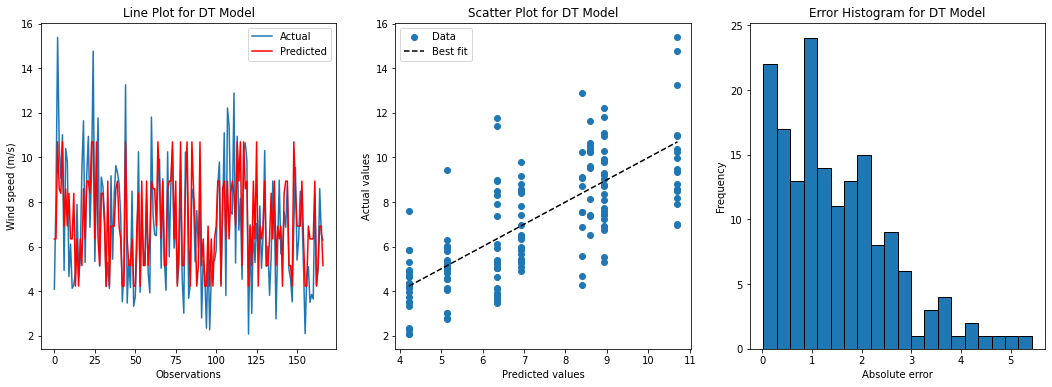 | 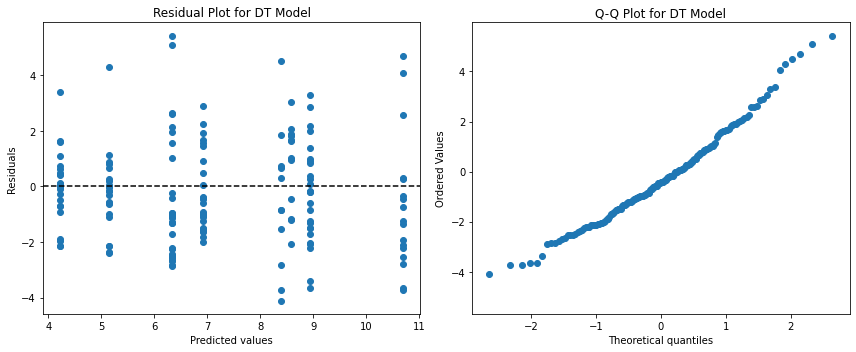 |
| 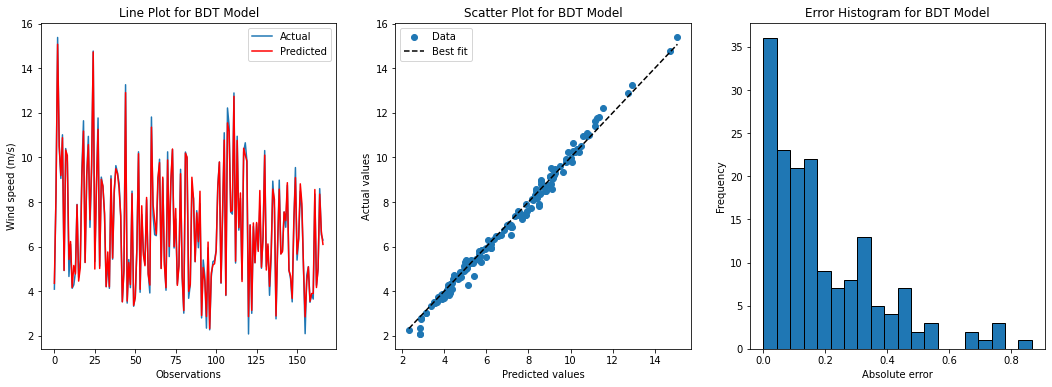 | 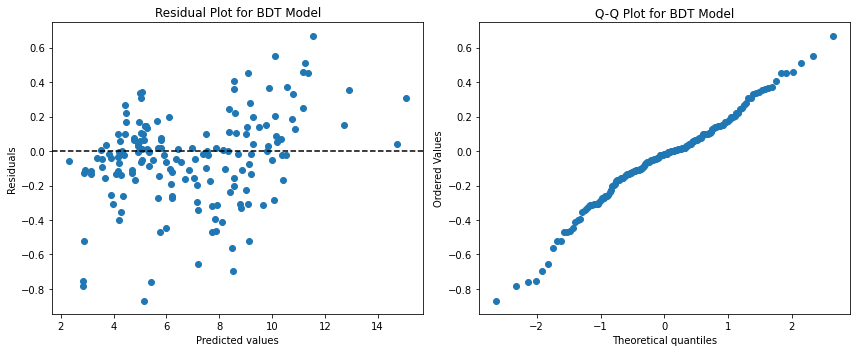 |
| 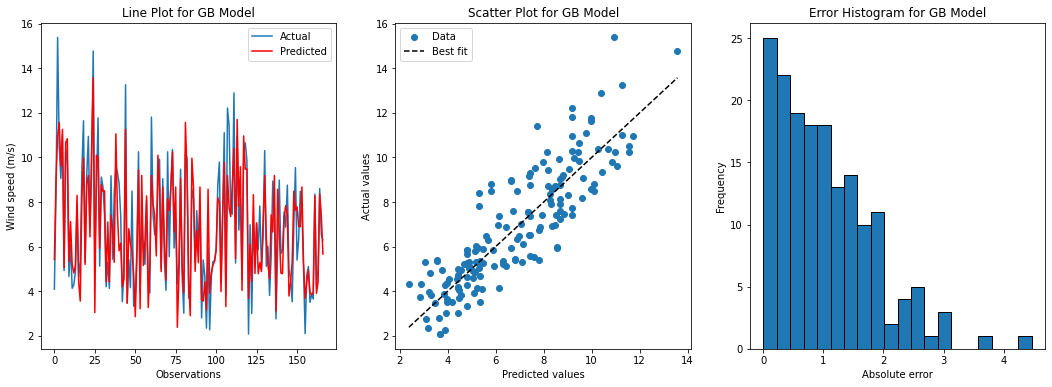 | 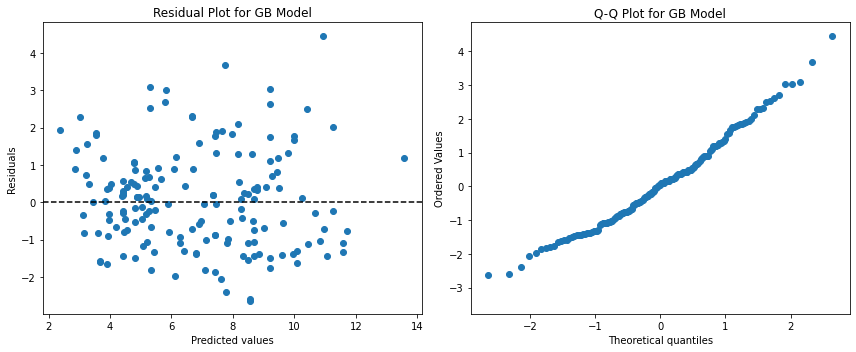 |
| 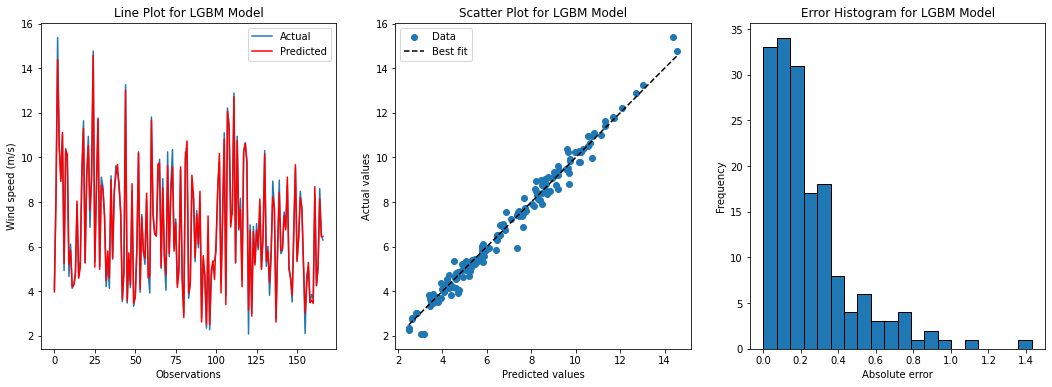 | 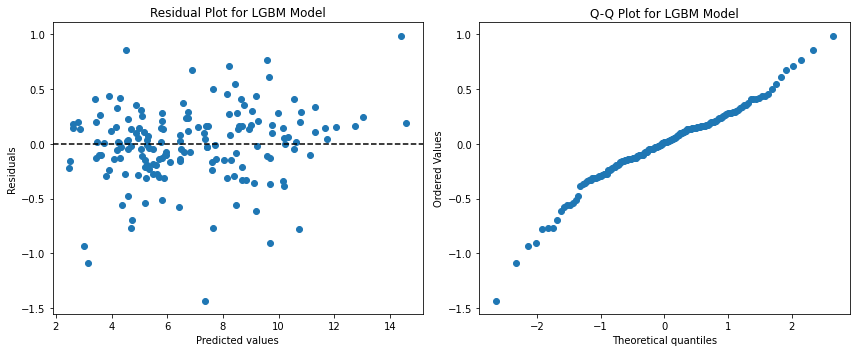 |
| 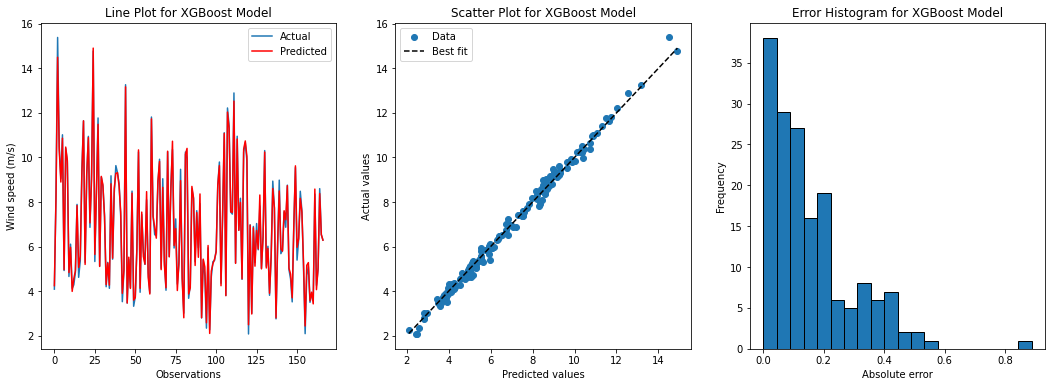 | 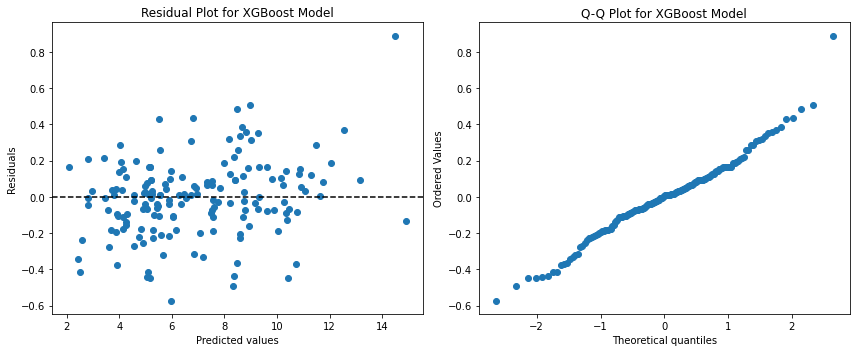 |
| 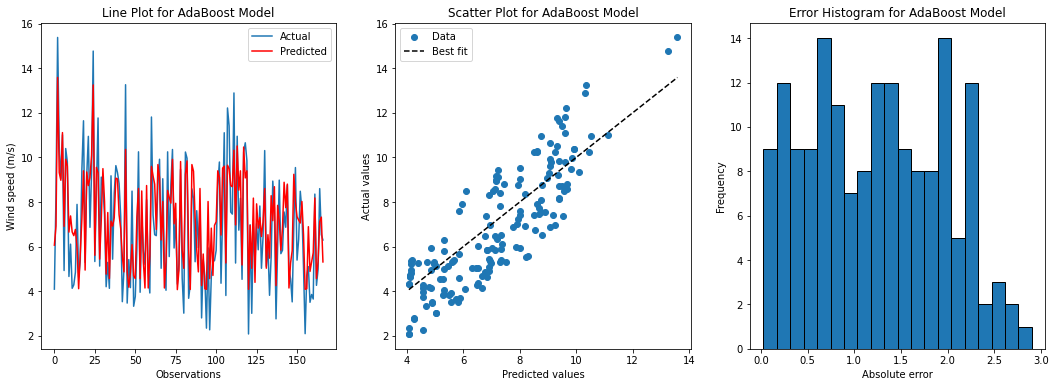 | 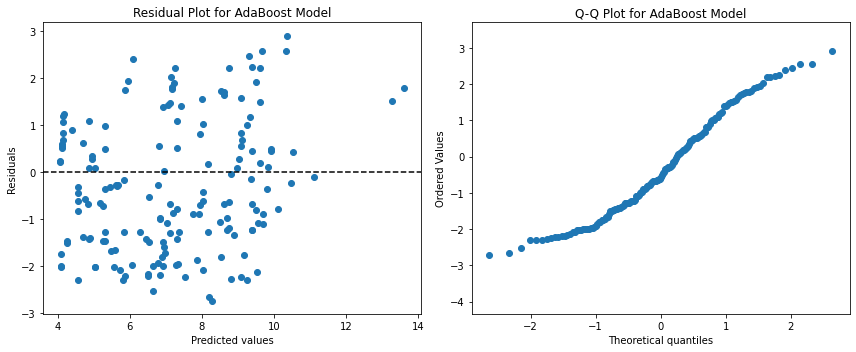 |

**Fig. B4** Visualization of the performance of developed models for predicting 24H ahead wind speed in terms of a) line plot, scatter plot, and error histogram and b) residual plot and q-q plot.

| 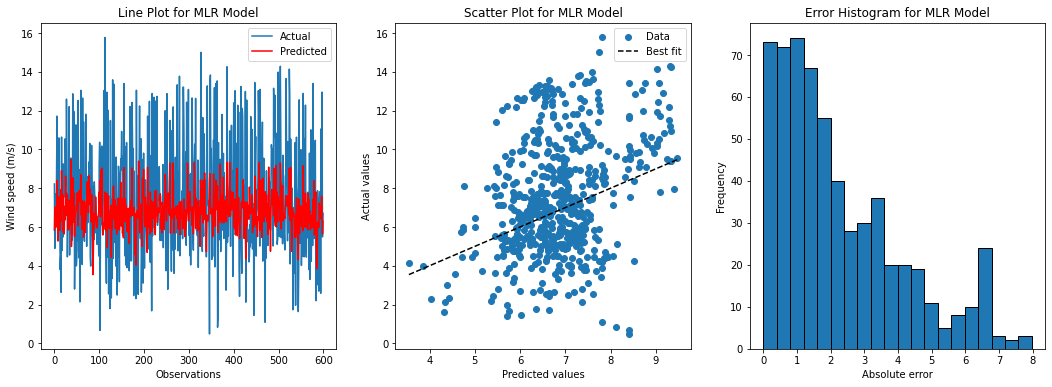 | 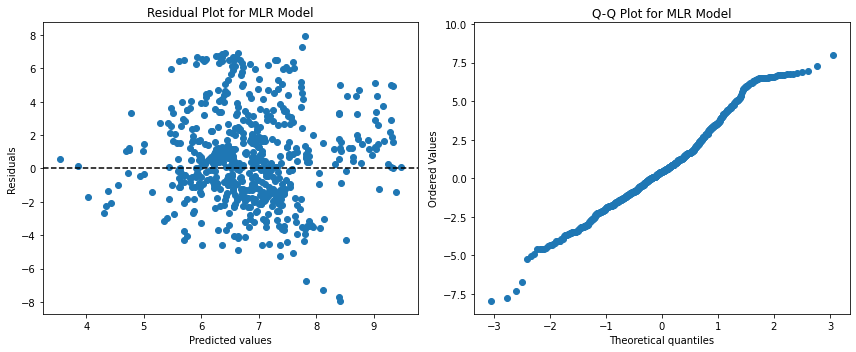 |
| --- | --- |
| 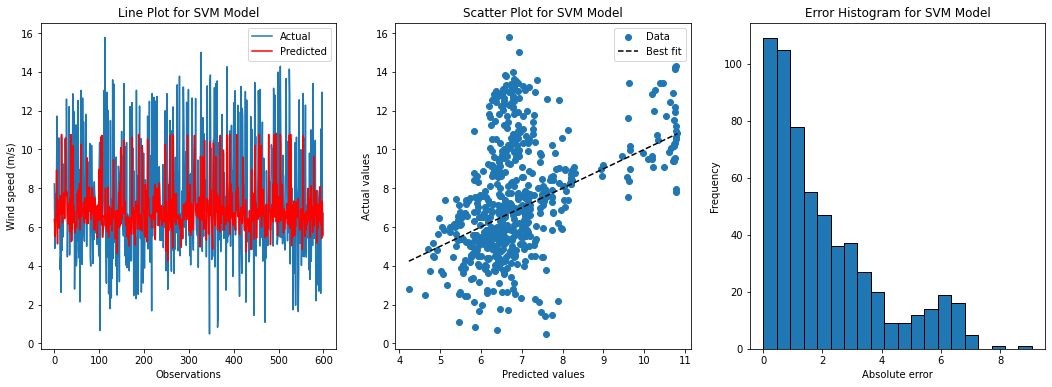 | 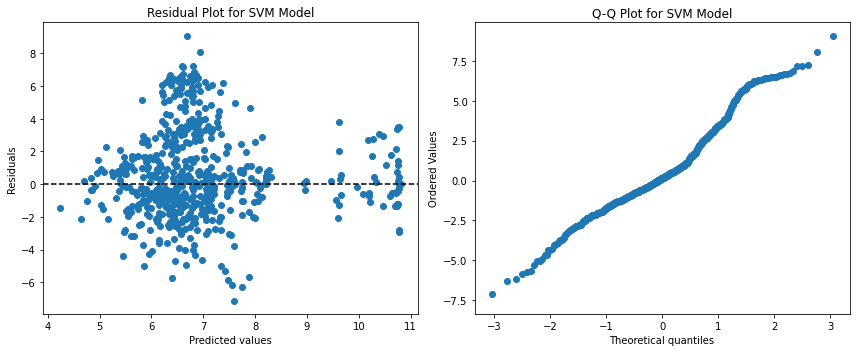 |
| 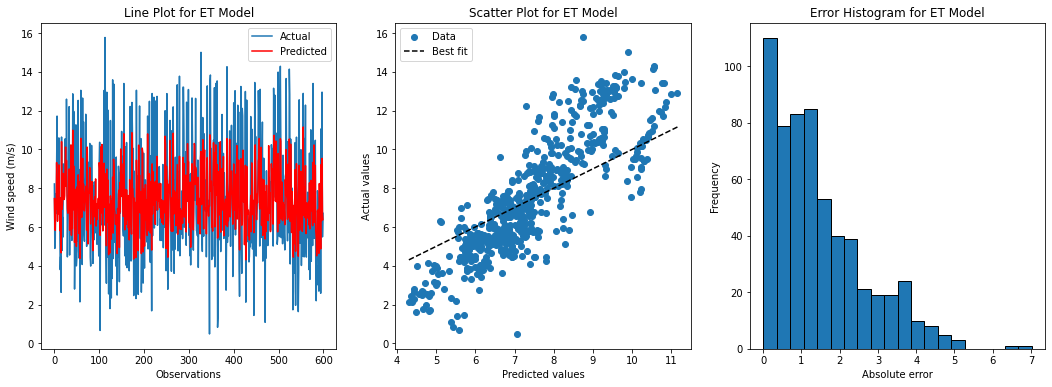 | 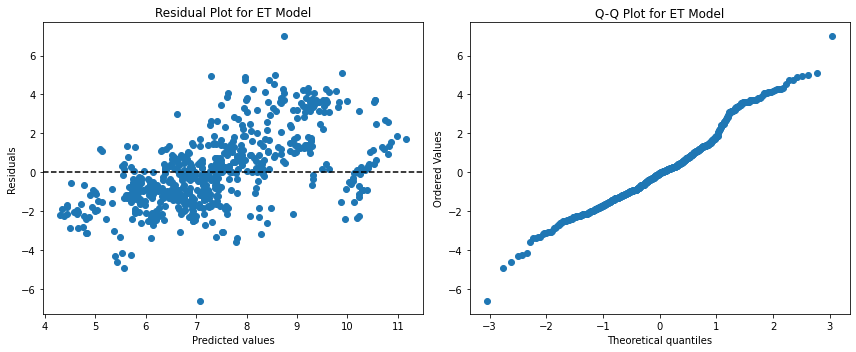 |
| 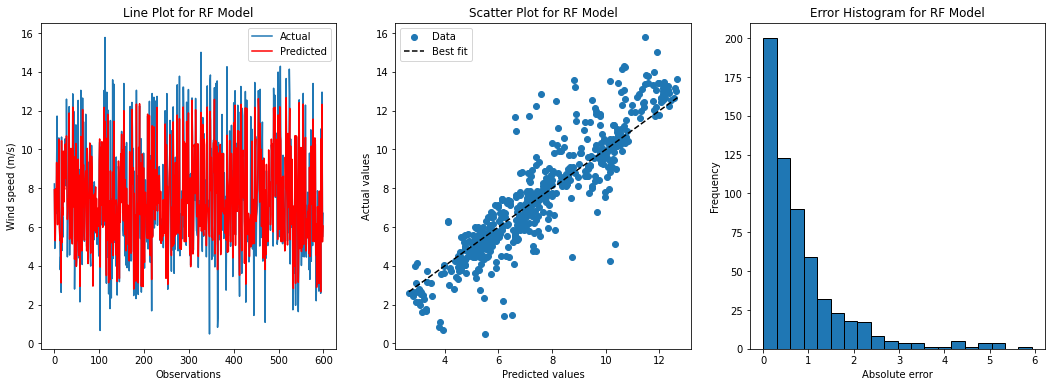 | 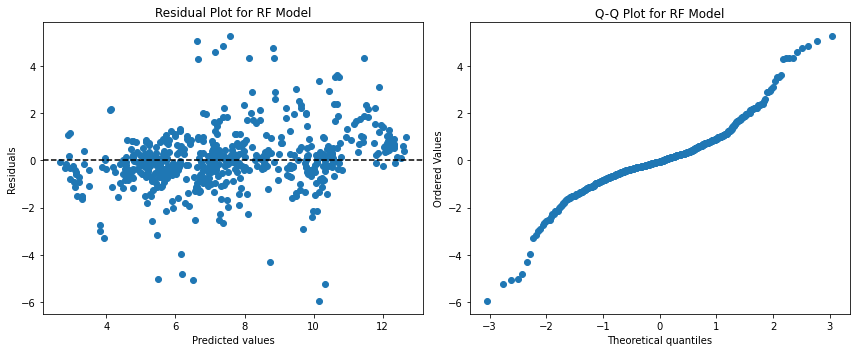 |
| 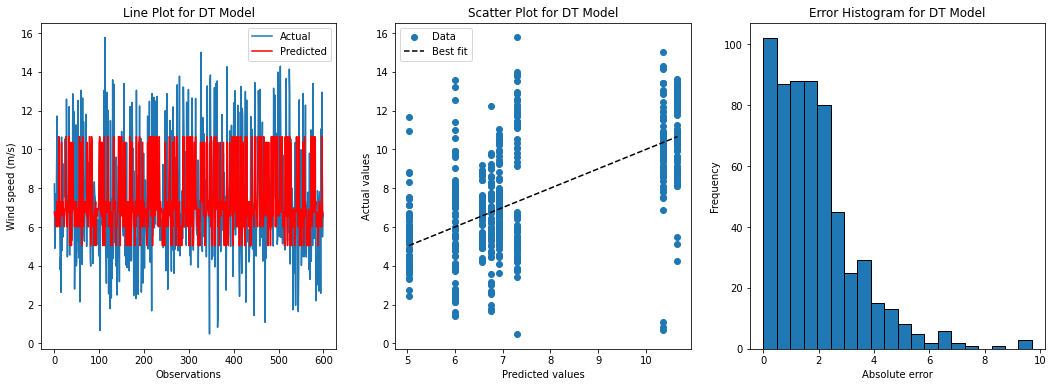 | 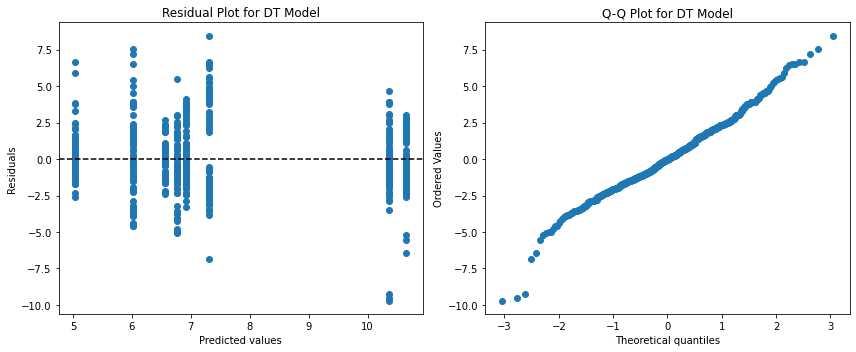 |
| 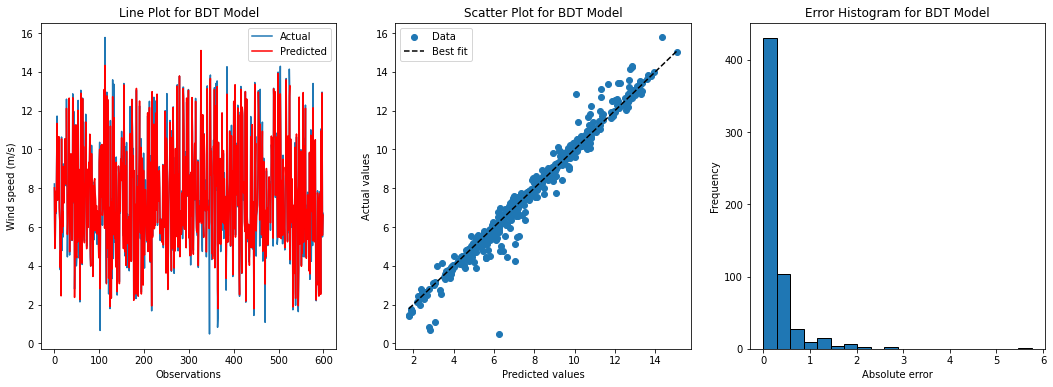 | 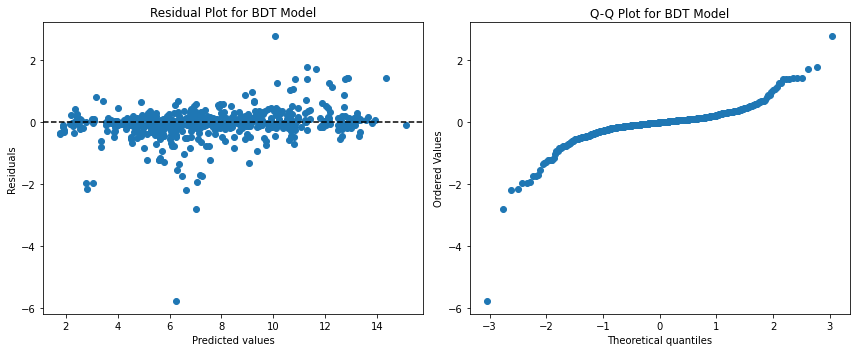 |
| 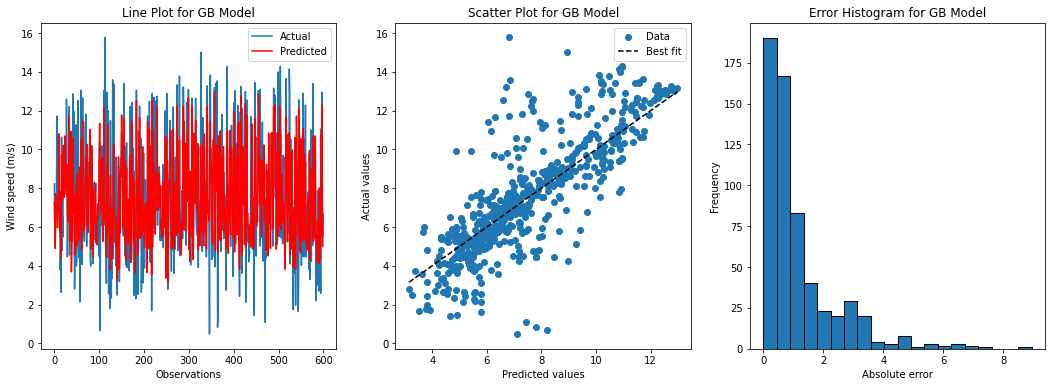 | 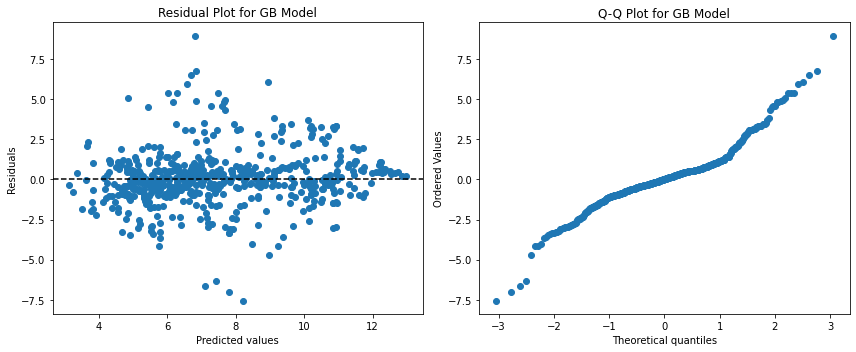 |
| 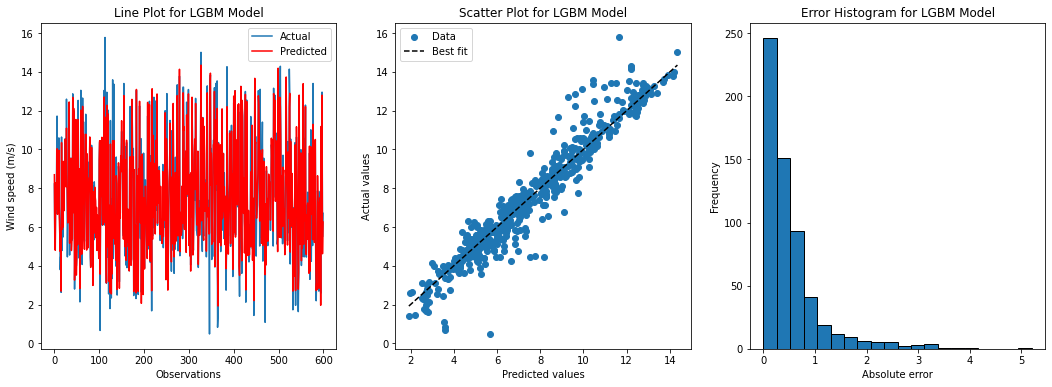 | 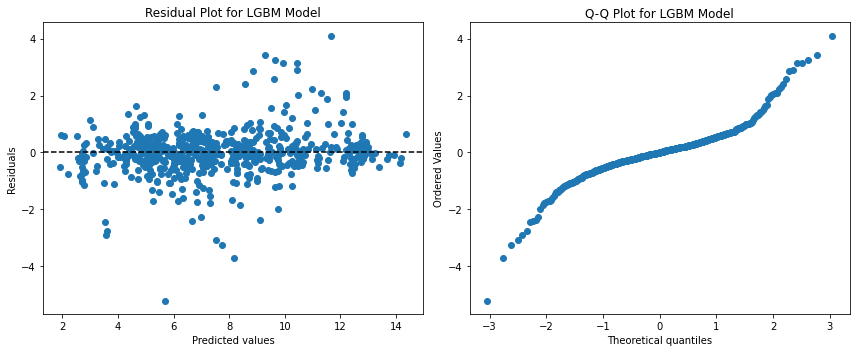 |
| 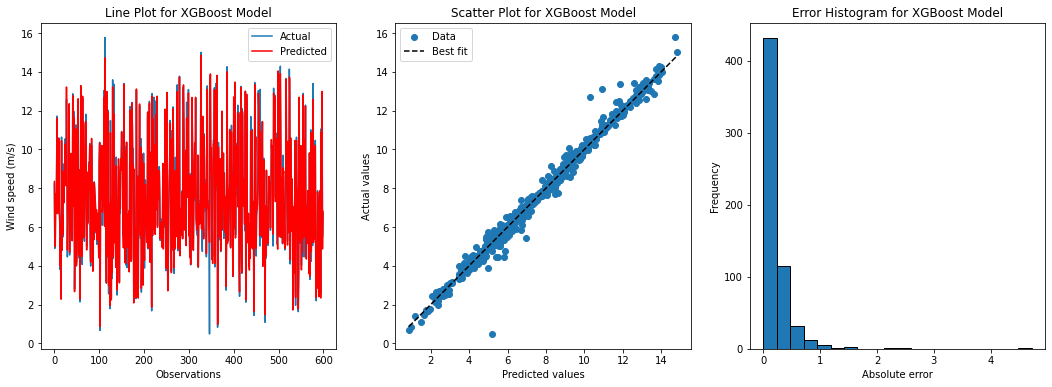 | 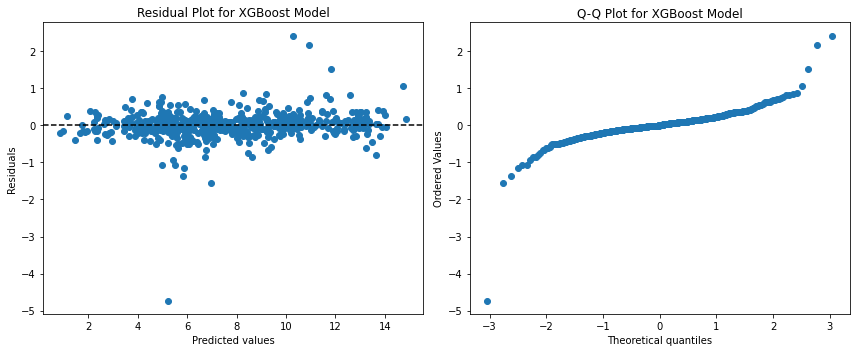 |
| 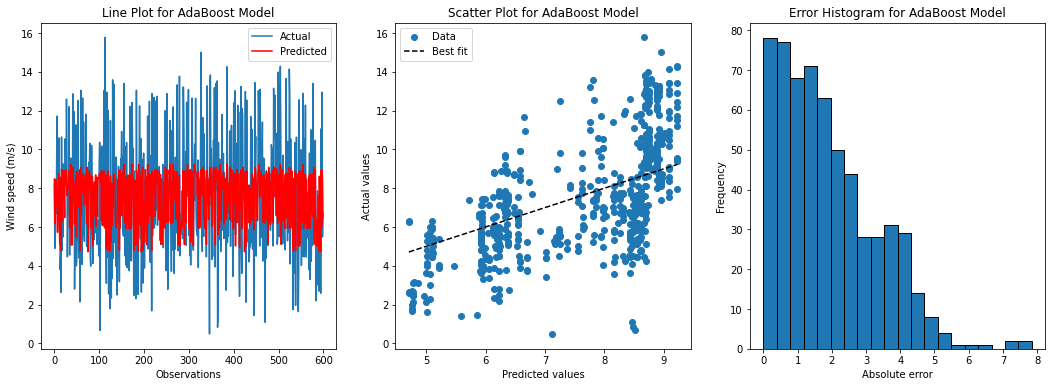 | 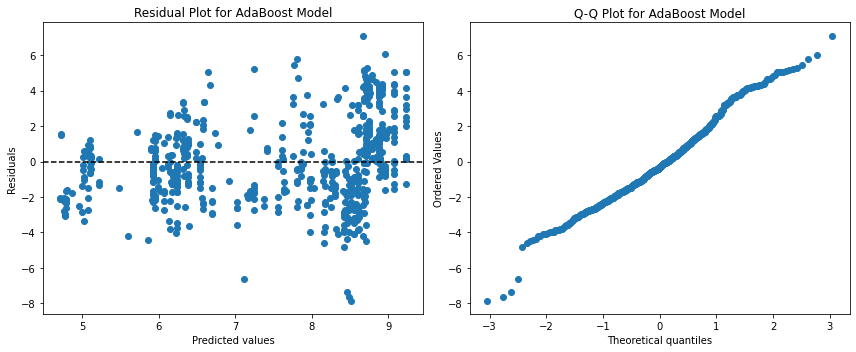 |

**Fig. B5** Visualization of the performance of developed models for predicting 36H ahead wind speed in terms of a) line plot, scatter plot, and error histogram and b) residual plot and q-q plot.

|  |  |
| --- | --- |
|  |  |
|  |  |
|  |  |
|  |  |
|  |  |
|  |  |
|  |  |
|  |  |
|  |  |

**Fig. B6** Visualization of the performance of developed models for predicting 10M ahead wind power in terms of a) line plot, scatter plot, and error histogram and b) residual plot and q-q plot.

|  |  |
| --- | --- |
|  |  |
|  |  |
|  |  |
|  |  |
|  |  |
|  |  |
|  |  |
|  |  |
|  |  |

**Fig. B7** Visualization of the performance of developed models for predicting 30M ahead wind power in terms of a) line plot, scatter plot, and error histogram and b) residual plot and q-q plot.

|  |  |
| --- | --- |
|  |  |
|  |  |
|  |  |
|  |  |
|  |  |
|  |  |
|  |  |
|  |  |
|  |  |

**Fig. B8** Visualization of the performance of developed models for predicting 6H ahead wind power in terms of a) line plot, scatter plot, and error histogram and b) residual plot and q-q plot.

|  |  |
| --- | --- |
|  |  |
|  |  |
|  |  |
|  |  |
|  |  |
|  |  |
|  |  |
|  |  |
|  |  |

**Fig. B9** Visualization of the performance of developed models for predicting 24H ahead wind power in terms of a) line plot, scatter plot, and error histogram and b) residual plot and q-q plot.

|  |  |
| --- | --- |
|  |  |
|  |  |
|  |  |
|  |  |
|  |  |
|  |  |
|  |  |
|  |  |
|  |  |

**Fig. B10** Visualization of the performance of developed models for predicting 36H ahead wind power in terms of a) line plot, scatter plot, and error histogram and b) residual plot and q-q plot.

**Appendix C.** Machine learning performance for wind power prediction

|  |  |
| --- | --- |
| (a) Beewarm plot | (b) Scatter matrix |

**Fig. C1** Beeswarm plot and scatter matrix of ML models for VSTWPP (10M)

|  |  |
| --- | --- |
| (a) 30M | (b) 6H |
|  |  |
| (c) 24H | (d) 36H |

**Fig. C2** Beeswarm plot for 30 minutes, 6 hours, 24 hours, and 36 hours ahead of WPP

|  |  |
| --- | --- |
| (a) 30M | (b) 6H |
|  |  |
| (c) 24H | (d) 36H |

**Fig. C3** Scatter matrix for 30 minutes, 6 hours, 24 hours, and 36 hours ahead of WPP
